# Semaglutide and Nonarteritic Anterior Ischemic Optic Neuropathy

**DOI:** 10.1001/jamaophthalmol.2024.6555

**Published:** 2025-02-20

**Authors:** Cindy X. Cai, Michelle Hribar, Sally Baxter, Kerry Goetz, Swarup S. Swaminathan, Alexis Flowers, Eric N. Brown, Brian Toy, Benjamin Xu, John Chen, Aiyin Chen, Sophia Wang, Cecilia Lee, Theodore Leng, Joshua R. Ehrlich, Andrew Barkmeier, Karen R. Armbrust, Michael V. Boland, David Dorr, Danielle Boyce, Thamir Alshammari, Joel Swerdel, Marc A. Suchard, Martijn Schuemie, Fan Bu, Anthony G. Sena, George Hripcsak, Akihiko Nishimura, Paul Nagy, Thomas Falconer, Scott L. DuVall, Michael Matheny, Benjamin Viernes, William O’Brien, Linying Zhang, Benjamin Martin, Erik Westlund, Nestoras Mathioudakis, Ruochong Fan, Adam Wilcox, Albert Lai, Jacqueline C. Stocking, Sahar Takkouche, Lok Hin Lee, Yangyiran Xie, Izabelle Humes, David B. McCoy, Mohammad Adibuzzaman, Raymond G. Areaux, William Rojas-Carabali, James Brash, David A. Lee, Nicole G. Weiskopf, Louise Mawn, Rupesh Agrawal, Hannah Morgan-Cooper, Priya Desai, Patrick B. Ryan

**Affiliations:** 1 Wilmer Eye Institute, Johns Hopkins School of Medicine, Baltimore, Maryland; 2 Biomedical Informatics and Data Science, Division of General Internal Medicine, Department of Medicine, Johns Hopkins University School of Medicine, Baltimore, Maryland; 3 National Eye Institute, National Institutes of Health, Bethesda, Maryland; 4 Casey Eye Institute, Department of Medical Informatics and Clinical Epidemiology, Oregon Health & Science University, Portland; 5 Viterbi Family Department of Ophthalmology and Shiley Eye Institute, University of California San Diego, La Jolla; 6 Division of Biomedical Informatics, Department of Medicine, University of California San Diego, La Jolla; 7 Department of Ophthalmology, Bascom Palmer Eye Institute, University of Miami Miller School of Medicine, Miami, Florida; 8 Vanderbilt Eye Institute, Department of Neurology, Vanderbilt University Medical Center, Nashville, Tennessee; 9 Vanderbilt Eye Institute, Department of Ophthalmology, Vanderbilt University Medical Center, Nashville, Tennessee; 10 Roski Eye Institute, Keck School of Medicine, University of Southern California, Los Angeles, California; 11 Department of Ophthalmology, Mayo Clinic, Rochester, Minnesota; 12 Department of Neurology, Mayo Clinic, Rochester, Minnesota; 13 Byers Eye Institute, Department of Ophthalmology, Stanford University, Palo Alto, California; 14 Department of Ophthalmology, University of Washington, Seattle; 15 Karalis Johnson Retina Center, Seattle, Washington; 16 Byers Eye Institute at Stanford, Stanford University School of Medicine, Palo Alto, California; 17 Department of Ophthalmology and Visual Sciences, University of Michigan, Ann Arbor; 18 Survey Research Center, Institute for Social Research, University of Michigan, Ann Arbor; 19 Department of Ophthalmology and Visual Neurosciences, University of Minnesota, Minneapolis, Minnesota; 20 Department of Ophthalmology, Mass Eye and Ear and Harvard Medical School, Boston, Massachusetts; 21 Department of Medical Informatics & Clinical Epidemiology, Portland, Oregon; 22 Johns Hopkins University School of Medicine, Baltimore, Maryland; 23 Tufts University School of Medicine, Boston, Massachusetts; 24 Department of Clinical Practice, Faculty of Pharmacy, Jazan University, Jazan, Saudi Arabia; 25 Pharmacy Practice Research Unit, Faculty of Pharmacy, Jazan University, Jazan, Saudi Arabia; 26 Janssen Research and Development, Titusville, New Jersey; 27 Department of Biostatistics, UCLA School of Public Health, University of California, Los Angeles, Los Angeles; 28 VA Informatics and Computing Infrastructure, US Department of Veterans Affairs, Salt Lake City, Utah; 29 Department of Biostatistics, University of Michigan, Ann Arbor; 30 Department of Medical Informatics, Erasmus University Medical Center, Rotterdam, the Netherlands; 31 Department of Biomedical Informatics, Columbia University, New York, New York; 32 Department of Biostatistics, Johns Hopkins Bloomberg School of Public Health, Baltimore, Maryland; 33 Department of Biomedical Informatics and Data Science, Johns Hopkins School of Medicine, Johns Hopkins University, Baltimore, Maryland; 34 Department of Internal Medicine, University of Utah School of Medicine, Salt Lake City; 35 VA Informatics and Computing Infrastructure, US Department of Veterans Affairs, Nashville, Tennessee; 36 Institute for Informatics, Data Science and Biostatistics, Department of Medicine, Washington University in St Louis, St Louis, Missouri; 37 Department of Medicine, Johns Hopkins University School of Medicine, Baltimore, Maryland; 38 Department of Medicine, Washington University in St Louis, St Louis, Missouri; 39 Department of Internal Medicine, University of California Davis, Sacramento; 40 Department of Medicine, Division of Diabetes and Endocrinology, Vanderbilt University, Nashville, Tennessee; 41 Vanderbilt University School of Medicine, Nashville, Tennessee; 42 Oregon Clinical and Translational Research Institute, Oregon Health & Science University, Portland; 43 Lee Kong Chian School of Medicine, Nanyang Technological University, Singapore; 44 IQVIA, Real World Solutions, Brighton, United Kingdom; 45 Ruiz Department of Ophthalmology and Visual Science, The University of Texas Health Science Center at Houston, McGovern Medical School, Houston; 46 Department of Medical Informatics and Clinical Epidemiology, Oregon Health & Science University, Portland; 47 Vanderbilt University Medical Center, Nashville, Tennessee; 48 Tan Tock Seng Hospital, Singapore; 49 Stanford School of Medicine and Stanford Health Care, Palo Alto, California; 50 Columbia University Irving Medical Center, New York, New York; 51 Johnson & Johnson, Horsham, Pennsylvania

## Abstract

**Importance:**

Semaglutide, a glucagonlike peptide-1 receptor agonist (GLP-1RA), has recently been implicated in cases of nonarteritic anterior ischemic optic neuropathy (NAION), raising safety concerns in the treatment of type 2 diabetes (T2D).

**Objective:**

To investigate the potential association between semaglutide and NAION in the Observational Health Data Sciences and Informatics (OHDSI) network.

**Design, Setting, and Participants:**

This was a retrospective study across 14 databases (6 administrative claims and 8 electronic health records). Included were adults with T2D taking semaglutide, other GLP-1RA (dulaglutide, exenatide), or non–GLP-1RA medications (empagliflozin, sitagliptin, glipizide) from December 1, 2017, to December 31, 2023. The incidence proportion and rate of NAION were calculated. Association between semaglutide and NAION was assessed using 2 approaches: an active-comparator cohort design comparing new users of semaglutide with those taking other GLP-1RAs and non–GLP-1RA drugs, and a self-controlled case-series (SCCS) analysis to compare individuals’ risks during exposure and nonexposure periods for each drug. The cohort design used propensity score–adjusted Cox proportional hazards models to estimate hazard ratios (HRs). The SCCS used conditional Poisson regression models to estimate incidence rate ratios (IRRs). Network-wide HR and IRR estimates were generated using a random-effects meta-analysis model.

**Exposures:**

GLP-1RA and non–GLP-1RAs.

**Main Outcomes and Measures:**

NAION under 2 alternative definitions based on diagnosis codes: one more inclusive and sensitive, the other more restrictive and specific.

**Results:**

The study included 37.1 million individuals with T2D, including 810 390 new semaglutide users. Of the 43 620 new users of semaglutide in the Optum’s deidentified Clinformatics Data Mart Database, 24 473 (56%) were aged 50 to 69 years, and 26 699 (61%) were female. The incidence rate of NAION was 14.5 per 100 000 person-years among semaglutide users. The HR for NAION among new users of semaglutide was not different compared with that of the non–GLP-1RAs using the sensitive NAION definition—empagliflozin (HR, 1.44; 95% CI, 0.78-2.68; *P* = .12), sitagliptin (HR, 1.30; 95% CI, 0.56-3.01; *P* = .27), and glipizide (HR, 1.23; 95% CI, 0.66-2.28; *P* = .25). The risk was higher only compared with patients taking empagliflozin (HR, 2.27; 95% CI, 1.16-4.46; *P* = .02) using the specific definition. SCCS analysis of semaglutide exposure showed an increased risk of NAION (meta-analysis IRR, 1.32; 95% CI, 1.14-1.54; *P* < .001).

**Conclusions and Relevance:**

Results of this study suggest a modest increase in the risk of NAION among individuals with T2D associated with semaglutide use, smaller than that previously reported, and warranting further investigation into the clinical implications of this association.

## Key Points

**Question:**

Is semaglutide use associated with the nonarteritic anterior ischemic optic neuropathy (NAION)?

**Findings:**

In this large multicenter study of 37.1 million adults with type 2 diabetes across 14 databases in the collaborative Observational Health Data Sciences and Informatics (OHDSI) network, a small increase was identified in the relative incidence of NAION from exposure to semaglutide compared with nonexposure.

**Meaning:**

These findings provide further evidence of an association between semaglutide and NAION but show a smaller risk than that previously reported; additional studies are necessary to identify potential mechanisms and causality.

## Teaser

This cohort study investigates the association between semaglutide use and nonarteritic anterior ischemic optic neuropathy.

## Introduction

Nonarteritic anterior ischemic optic neuropathy (NAION) is the leading cause of acute optic neuropathy in older adults.^1,2,3^ A recent single-center study demonstrated a 4.28-fold (95% CI, 1.62-11.29) increased risk of NAION with semaglutide, a glucagonlike peptide-1 receptor agonist (GLP-1RA), relative to non–GLP-1RAs.^4,5^ GLP-1RAs, approved by the US Food and Drug Administration (FDA) for type 2 diabetes (T2D) treatment in 2017, offer well-documented benefits in reducing cardiovascular and kidney complications.^6,7,8,9,10,11^ Given these advantages, the American Diabetes Association recommends GLP-1RAs as a preferred therapy for patients with T2D with atherosclerotic cardiovascular disease, chronic kidney disease, or obesity.^6^

There is a need to clarify the potential risk of ophthalmic complications from semaglutide exposure using multicenter data. This study leveraged the Observational Health Data Sciences and Informatics (OHDSI) network to investigate the association between semaglutide exposure and NAION. The OHDSI network is an international open-science collaborative centered around the Observational Medical Outcomes Partnership (OMOP) Common Data Model (CDM).^12^ The OHDSI network and its open-source analytic tools have been previously used to generate reliable evidence in medicine, including ophthalmology.^7,8,11,12,13,14^ We aimed to (1) characterize NAION incidence, (2) compare the risk of NAION associated with semaglutide use against other GLP-1RAs and non–GLP-1RA drugs, and (3) investigate NAION incidence rate during semaglutide exposure compared with nonexposure.

## Methods

### Study Design

We retrospectively analyzed 14 OHDSI databases (6 administrative claims and 8 electronic health records) using 2 approaches: (1) an active-comparator new-user cohort design to test whether NAION risk is higher with semaglutide compared with other GLP-1RA and non–GLP-1RAs and (2) a self-controlled case-series method to test whether NAION risk was higher with semaglutide exposure compared with nonexposure.^15,16^ Database details are in eTable 1 in Supplement 1. All participating sites had local institutional review board approvals where informed consent was waived because this was considered secondary research or exemptions. The study adhered to the tenets of the Declaration of Helsinki. This study followed the Strengthening the Reporting of Observational Studies in Epidemiology (STROBE) reporting guidelines.^17^

### Participants and Exposure

Adults 18 years and older with T2D taking semaglutide (GLP-1RA), dulaglutide (GLP-1RA), exenatide (GLP-1RA), empagliflozin (sodium-glucose cotransporter 2 [SGLT2] inhibitor), sitagliptin (dipeptidyl peptidase 4 [DPP4] inhibitor), or glipizide (sulfonylurea) during the study period (December 1, 2017-December 31, 2023) were included. For the non–GLP-1RA comparator medications, the most commonly used within each drug class was chosen based on the utilization results from the Large-Scale Evidence Generation and Evaluation Across a Network of Databases for Type 2 Diabetes Mellitus (LEGEND-T2DM) study, an OHDSI network study examining second-line antihyperglycemic agents and cardiovascular outcomes.^7,8,11^ Participants self-identified races and ethnicities were categorized according to local database guidelines and could include Asian, Black or African American, Hispanic or Latino, not Hispanic or Latino, and White.

### Time at Risk

The time-at-risk period began with medication initiation until the end of continuous drug exposure, defined as a gap in exposure of more than 30 days or the end of the continuous observation period.

### Outcome

The outcome of interest was NAION, determined by diagnosis codes. A consortium of board-certified ophthalmologists including fellowship-trained neuro-ophthalmologists developed 2 definitions: the sensitive definition required 1 ischemic optic neuropathy diagnosis code, whereas the specific definition required a second confirmatory ischemic optic neuropathy diagnosis code within 90 days. For both definitions, patients with traumatic optic neuropathy or 2 diagnosis codes of giant cell arteritis were excluded. If patients had diagnosis codes within 60 days before the NAION diagnosis related to optic disc disorders, optic neuritis, or optic disc edema, the date of the NAION outcome was shifted to the earlier diagnosis because it was likely related to the NAION event.^18,19,20^ Details are in eTables 2 and 3 in Supplement 1 and eTable 4 in Supplement 2.

The sensitivity, specificity, positive predictive value, and negative predictive value of both NAION definitions were evaluated on a subset of databases using PheValuator.^21^ PheValuator is an OHDSI tool that uses machine learning to compute a patient’s probability of having a disease of interest to identify a set of probabilistic gold standards, or patients with high likelihood of having the disease. The probabilistic gold standard dataset was used to determine the performance metrics of the consortium-developed rule-based definitions. Additional details are on the study protocol page.^22^

### Negative Control Outcomes

In addition to the primary outcome, NAION, we included 97 outcomes believed to be causally unrelated to the exposures under investigation.^22^ To detect and adjust for systematic errors from residual confounding, both the cohort analysis and self-controlled case-series analysis were performed using these negative control outcomes.^23,24^

### Statistical Analysis

All analyses were performed in R, version 4.2.3 (R Project for Statistical Computing).^25,26^ Analysis codes are available on Github.^16^ All *P* values were 2-sided, and *P* <.05 was considered statistically significant. Results can also be found on an interactive website.^27^

#### Characterization

We summarized the baseline characteristics of patients within each T2DM medication exposure cohort by database.^28^ These characteristics included demographics (age, sex, race, and ethnicity), Diabetes Comorbidity Severity Index score, Charlson Comorbidity Index-Romano adaptation, prior medical diagnoses (essential hypertension, hyperlipidemia, obstructive sleep apnea, chronic kidney disease, and anemia), and prior medication use (interferon, amiodarone, and phosphodiesterase inhibitor).^29,30^ NAION incidence proportion (number of patients with NAION divided by persons at risk) and incidence rate (number of patients with NAION during time-at-risk divided by person-days) were calculated.^28,31^

#### Active-Comparator New-User Cohort Analysis

An active-comparator new-user cohort design was used to estimate propensity score–adjusted hazard ratios (HRs) for NAION comparing new semaglutide users with users of comparator medications: GLP-1RAs (dulaglutide and exenatide) and non–GLP-1RA drugs (empagliflozin, sitagliptin, and glipizide).^32^ New users were defined as previously described.^7,8,11^ In brief, adults with T2D taking metformin monotherapy were included if they had at least 1 year of prior observation, initiated treatment with one of the medications of interest, had no prior exposure to a comparator diabetes medication, and had at most 30 days of prior insulin use.

Propensity scores were estimated using a large-scale propensity score approach, applying regularized regression over all baseline characteristics (eg, demographic characteristics, preexisting conditions, medications, and procedures).^33,34^ Patients in each target and comparator exposure comparison (eg, semaglutide vs dulaglutide) were matched 1:1 using propensity scores. Cox proportional hazards models estimated the HR of NAION from cohort entry to the outcome while taking treatment with each target and comparator T2D medication.

#### Sensitivity Analyses

We used 2 sensitivity analyses to test robustness. To assess sensitivity to the cohort definition, we created a second definition to include new users of each T2D medication regardless of prior exposure to a comparator drug. Details can be found in eTables 2 and 3 in Supplement 1 and eTable 4 in Supplement 2.^22^ We also stratified our propensity score–matched Cox proportional hazards models by calendar time (December 2017-January 2020, February 2020-June 2021, and July 2021-December 2023). This addressed potential biases due to decreased health care utilization from the COVID-19 pandemic, and the 60% increased prescription of semaglutide from 2021 to 2023 after FDA approval for obesity.^22,35^

#### Self-Controlled Case-Series Analysis

A self-controlled case series, where patients served as their own controls, was used to estimate the NAION incidence rate ratio (IRR) during each T2D medication exposure compared with nonexposure control time.^36,37,38^ Real-world data evaluation of study designs have found that this approach can accurately identify causal associations between drug exposures and health outcomes.^36,38,39^ We restricted the observation period to when patients had T2D and excluded the patients’ first 365 days in the database from the analysis to improve the identification of incident NAION. We used conditional Poisson regression models to compare the NAION IRRs.^40^ We defined a separate preexposure time window, during the control time, as the period of 30 days before treatment initiation and adjusted for it.^40,41^ The models also adjusted for potential effects of seasonality by including spline functions of calendar months.

#### Study Diagnostics and Meta-Analysis

For both cohort and self-controlled case-series analyses, we used a comprehensive set of study diagnostics, eg, preexposure patient characteristic balance, empirical clinical equipoise, minimum power requirement, and negative control experiments, to evaluate systematic error, residual bias, reliability, and the generalizability of our comparisons.^23,24,36,42,43^ Only databases that passed prespecified study diagnostics reported HR or IRR estimates.^22^ A bayesian random-effects meta-analysis combined each database’s estimates into a network-wide estimate.^44,45^

## Results

In total, 14 OHDSI network databases were included in the analysis; all 14 were included in the NAION incidence analysis, but only 8 in the active-comparator new-user cohort analysis and 10 in the self-controlled case-series analysis passed study diagnostics (eTable 1 in Supplement 1). Results from the diagnostics are shown in eTables 5 to 8 in Supplement 1.

PheValuator confirmed that the sensitive NAION definition has higher sensitivities (mean 73% vs 33%), whereas the specific NAION definition has higher positive predictive values (88% vs 71%) (eTable 9 in Supplement 1).

### NAION Incidence

Across all databases, there were 37.1 million patients with T2D, among whom 810 390 were new semaglutide users, 326 282 dulaglutide, 25 936 exenatide, 715 802 empagliflozin, 493 563 sitagliptin, and 832 295 glipizide. Baseline patient characteristics in the Optum’s deidentified Clinformatics Data Mart Database are presented in the Table, as a representative example. There were a total of 166 932 patients in all T2D drug exposure cohorts, with 43 620 new users of semaglutide, of whom 24 473 (56%) were aged 50 to 69 years, 26 699 (61%) were female, and 16 921 (39%) were male. Patients self-identified with the following race ethnicities: 1179 Asian (3%), 5748 Black or African American (13%), 4920 Hispanic (11%), 35 562 not Hispanic or Latino (82%), and 28 635 White (66%). The baseline patient characteristics of all other databases are in eTable 10 in Supplement 1.

**Table.** Baseline Characteristics of Patients in Each Type 2 Diabetes Drug Exposure Cohort (Semaglutide, Dulaglutide, Exenatide, Empagliflozin, Sitagliptin, Glipizide) During the Study Period in the Optum’s Deidentified Clinformatics Data Mart Database (Clinformatics)

Variable	Patients, No. (%)					
	GLP-1 RA			Empagliflozin (SGLT2 inhibitor)	Sitagliptin (DPP4 inhibitor)	Glipizide (sulfonylurea)
	Semaglutide	Dulaglutide	Exenatide			
No.	43 620	14 923	1414	43 302	19 581	44 092
Age, y^a^						
≤29	453 (1)	168 (1)	23 (1)	165 (0)	29 (0)	174 (0)
30-49	8110 (19)	3095 (21)	315 (22)	5122 (12)	875 (4)	4478 (10)
50-69	24 473 (56)	8238 (55)	832 (59)	21 614 (50)	7292 (37)	20 330 (46)
≥70	10 586 (24)	3426 (23)	248 (18)	16 401 (38)	11 389 (58)	19 111 (43)
Race						
Asian	1179 (3)	332 (2)	37 (3)	2333 (5)	961 (5)	2008 (5)
Black or African American	5748 (13)	1939 (13)	166 (12)	5228 (12)	3020 (15)	5458 (12)
White	28 635 (66)	9976 (67)	900 (64)	27 337 (63)	11 283 (58)	26 464 (60)
Ethnicity						
Hispanic or Latino	4920 (11)	1703 (11)	228 (16)	5329 (12)	2892 (15)	6836 (16)
Not Hispanic or Latino	35 562 (82)	12 247 (82)	1103 (78)	34 898 (81)	15 264 (78)	33 930 (77)
Sex						
Female	26 699 (61)	8343 (56)	797 (56)	17 872 (41)	10 683 (55)	20 587 (47)
Male	16 921 (39)	6580 (44)	617 (44)	25 430 (59)	8898 (45)	23 505 (53)
DCSI, mean (SD)	2.57 (1.97)	2.64 (1.97)	2.72 (2.08)	3.25 (2.25)	3.57 (2.39)	3.26 (2.31)
CCI, mean (SD)	4.04 (2.37)	4.05 (2.33)	3.92 (2.23)	4.69 (2.67)	5.03 (2.71)	4.64 (2.68)
Essential hypertension	33 537 (77)	11 420 (77)	1087 (77)	34 789 (80)	16 441 (84)	35 070 (80)
Hyperlipidemia	34 056 (78)	11 393 (76)	1079 (76)	35 942 (83)	16 342 (83)	34 918 (79)
Obstructive sleep apnea	12 079 (28)	3729 (25)	348 (25)	8654 (20)	2905 (15)	6142 (14)
Chronic kidney disease	4776 (11)	1670 (11)	134 (9)	7358 (17)	4142 (21)	8067 (18)
Anemia	6373 (15)	1859 (12)	172 (12)	7048 (16)	3888 (20)	7178 (16)
Interferon use	6 (0)	5 (0)	0	7 (0)	<5 (0)	9 (0)
Amiodarone use	218 (0)	89 (1)	10 (1)	840 (2)	248 (1)	473 (1)
Phosphodiesterase inhibitor use	0	0	0	6 (0)	<5 (0)	<5 (0)

Abbreviations: CCI, Charlson Comorbidity Index-Romano adaptation; DCSI, Diabetes Complications Severity Index; DPP4, dipeptidyl peptidase-4; GLP-1 RA, glucagonlike peptide 1 receptor agonist; SGLT2, sodium-glucose cotransporter 2.

^a^ In each database, age groups span 5 years, and counts fewer than 5 are reported as <5. When combining age groups for reporting, all such categories were treated as having 5 patients.

**Figure 1.**
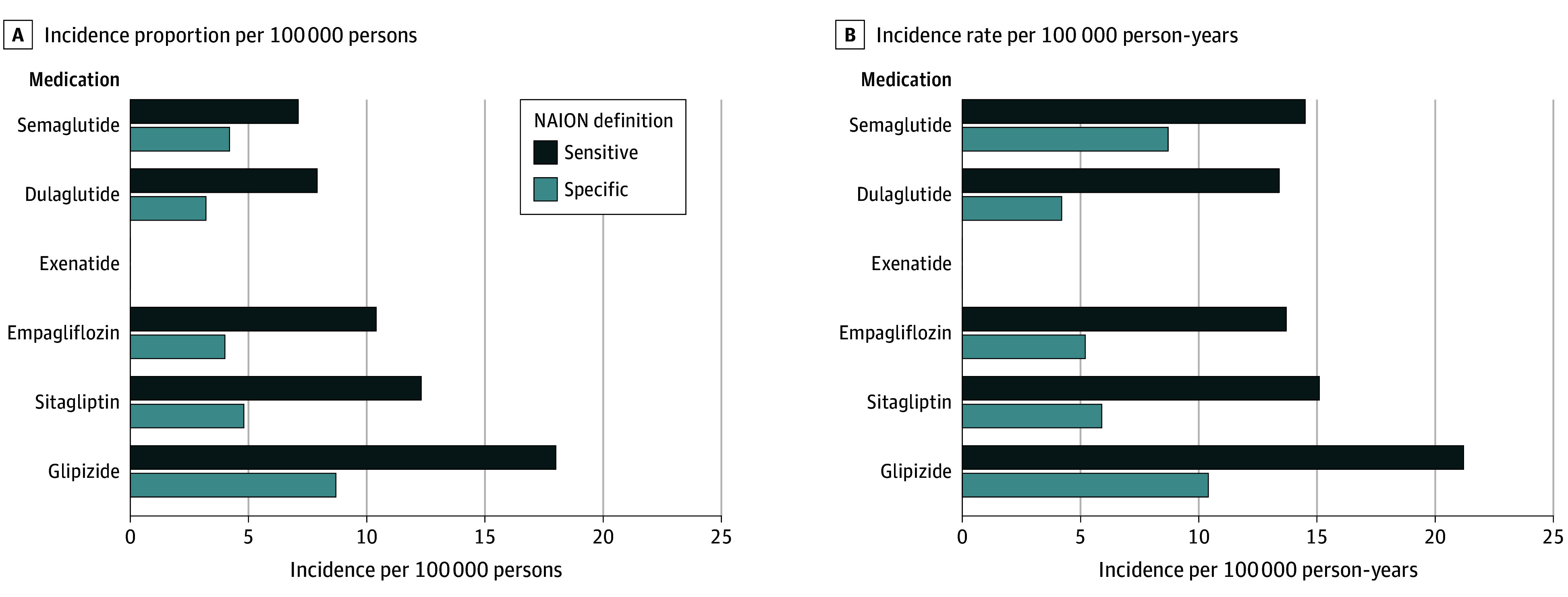
Incidence of Nonarteritic Anterior Ischemic Optic Neuropathy (NAION) Among Patients With Type 2 Diabetes

Among new semaglutide users, the NAION incidence proportions were 7.1 and 4.2 per 100 000 persons, and the incidence rates were 14.5 and 8.7 per 100 000 person-years, for the sensitive and specific definitions, respectively (Figure 1 and eTable 11 in Supplement 1).

### Active-Comparator New-User Cohort Analysis

**Figure 2.**
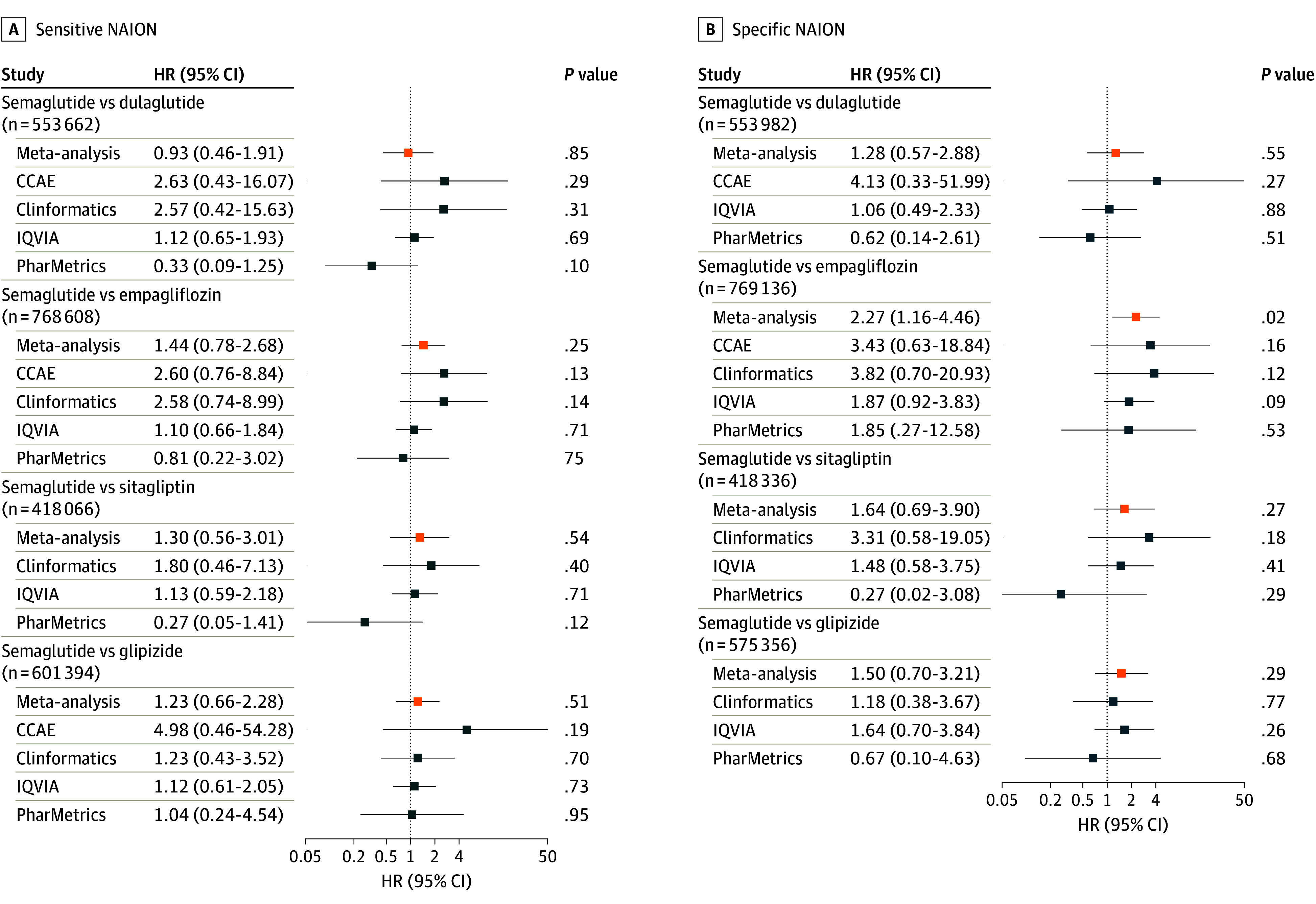
Forest Plot for the Active-Comparator New-User Cohort Analysis Hazard ratio (HR) and 95% CI estimates for the risk of nonarteritic anterior ischemic optic neuropathy (NAION) while receiving treatment with a second-line medication for type 2 diabetes comparing between semaglutide and other glucagonlike peptide-1 receptor agonists (GLP-1RAs), ie, dulaglutide and exenatide, and non–GLP-1RA medications, ie, empagliflozin, sitagliptin, and glipizide. Only results from databases and comparisons that passed study diagnostics are provided, as well as the meta-analytic estimates.^a^ The total number of patients in each comparison is shown. A, Results using the sensitive NAION definition. B, Results using the specific NAION definition.^b ^CCAE indicates Merative MarketScan Commercial Claims and Encounters Database; Clinformatics, Optum’s deidentified Clinformatics Data Mart Database; IQVIA, IQVIA Open Claims; PharMetrics, PharMetrics Plus. ^a^Databases that have 0 cases of NAION are unable to produce HR estimates but still contribute to the meta-analytic estimate. ^b^The sensitive NAION definition only required 1 diagnosis of ischemic optic neuropathy, and the specific NAION definition required 2 diagnoses. All other criteria were the same between the 2 definitions.

The risk for NAION using the sensitive definition was not different among new semaglutide users compared with dulaglutide (meta-analysis HR, 0.93; 95% CI, 0.46-1.91; *P* = .57) or with the non–GLP-1RA medications, empagliflozin (HR, 1.44; 95% CI, 0.78-2.68; *P* = .12), sitagliptin (HR, 1.30; 95% CI, 0.56-3.01; *P* = .27), and glipizide (HR, 1.23; 95% CI, 0.66-2.28; *P* = .25) (Figure 2 and eFigures 1 and 2 in Supplement 1). Similarly, using the specific definition, the risk was not different among new users of semaglutide compared with dulaglutide (HR, 1.28; 95% CI, 0.57-2.88; *P* = .55), sitagliptin (HR, 1.64; 95% CI, 0.69-3.90; *P* = .27), and glipizide (HR, 1.50; 95% CI, 0.70-3.21; *P* = .29). There was, however, increased NAION risk among new semaglutide users compared with empagliflozin (HR, 2.27; 95% CI, 1.59-4.46; *P* = .02) using the specific definition (Figure 2).

Results were similar in the 2 sensitivity analyses (eFigures 3 and 4 in Supplement 1). The risk of NAION was higher among semaglutide users regardless of prior medication exposure compared with empagliflozin (both definitions) and with sitagliptin (specific definition). NAION risk was also higher among semaglutide users from 2021 to 2023 compared with empagliflozin using both NAION definitions.

### Self-Controlled Case-Series Analysis

**Figure 3.**
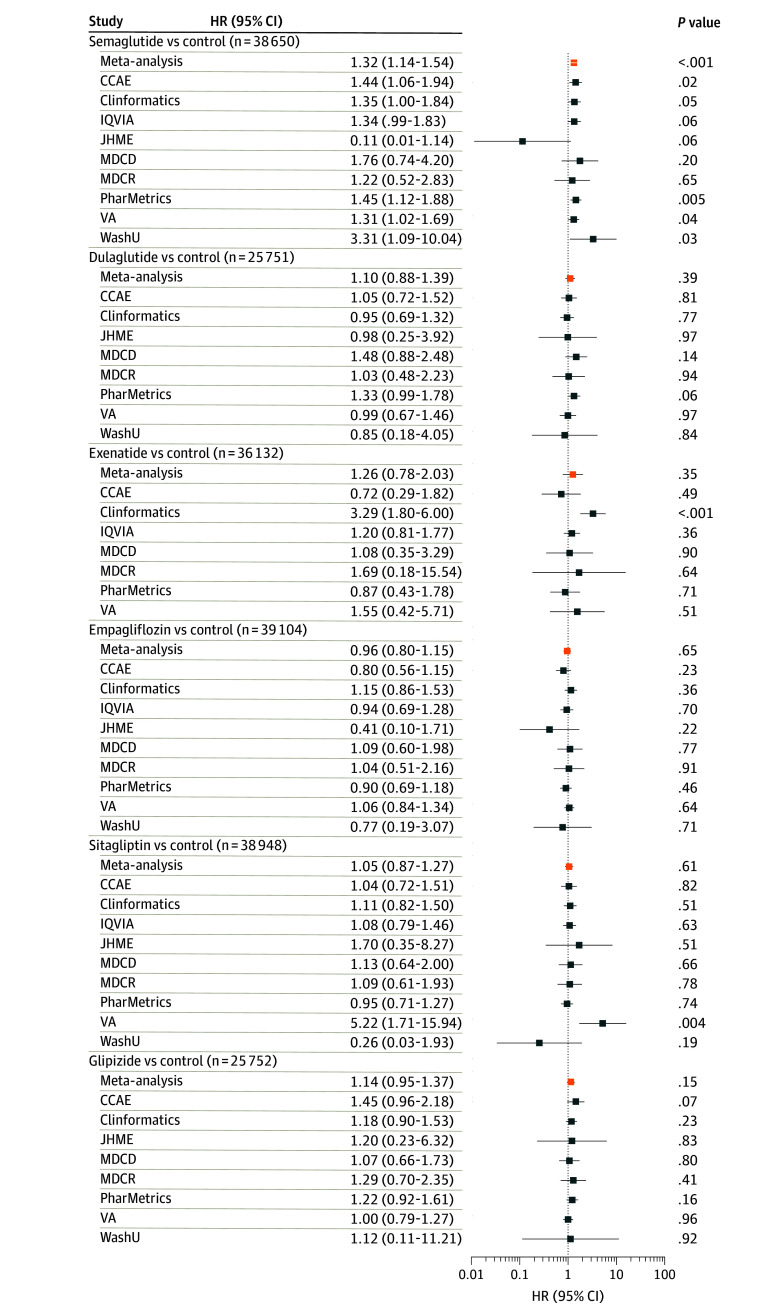
Forest Plot for the Self-Controlled Case Series Analysis, Sensitive Nonarteritic Anterior Ischemic Optic Neuropathy (NAION) Definition^a^ Incidence rate ratio (IRR) and 95% CI estimate for the risk of NAION (using the sensitive definition)^a^ while receiving treatment with one of the medications for type 2 diabetes compared with control time, not taking treatment with the medication of interest. Results are shown for semaglutide, other glucagonlike peptide-1 receptor agonists (GLP-1 RAs), ie, dulaglutide and exenatide, and non–GLP-1 RA medications, ie, empagliflozin, sitagliptin, and glipizide. Results from databases that passed study diagnostics are provided, as well as the meta-analytic estimates.^b^ The total number of patients included is shown. CCAE indicates Merative MarketScan Commercial Claims and Encounters Database; Clinformatics, Optum’s deidentified Clinformatics Data Mart; IQVIA, IQVIA Open Claims; JHME, Johns Hopkins Medical Enterprise; MDCD, Merative MarketScan Multi-State Medicaid Database; MDCR, Merative MarketScan Medicare Supplemental and Coordination of Benefits Database; PharMetrics, PharMetrics Plus; VA, Department of Veterans Affairs; WashU, Washington University in St Louis. ^a^The sensitive NAION definition only required 1 diagnosis of ischemic optic neuropathy. All other criteria were the same between the 2 definitions. ^b^Due to data privacy issues, databases that have counts less than 5 are unable to produce IRR estimates but still contribute to the meta-analytic estimate.

**Figure 4.**
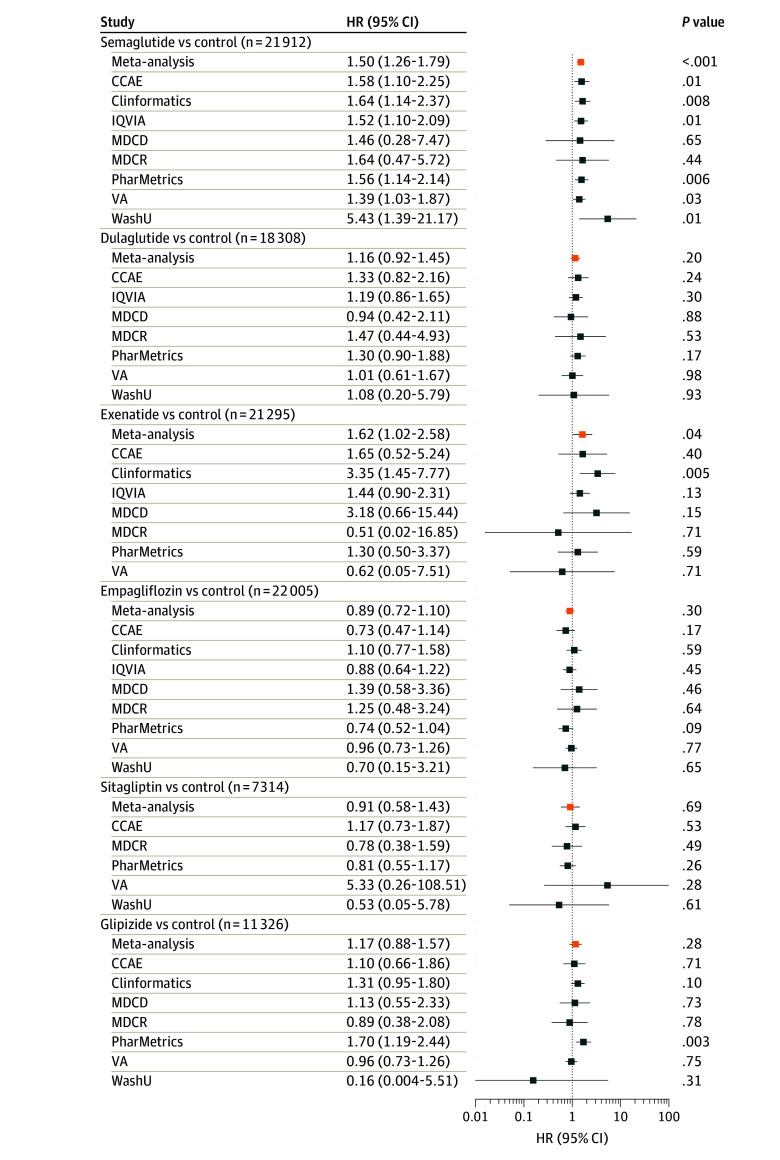
Forest Plot for the Self-Controlled Case Series Analysis, Specific Nonarteritic Anterior Ischemic Optic Neuropathy (NAION) Definition^a^ Incidence rate ratio (IRR) and 95% CI estimate for the risk of NAION (using the specific definition)^a^ while receiving treatment with one of the medications for type 2 diabetes compared with control time, not taking treatment with the medication of interest. Results are shown for semaglutide, other glucagonlike peptide-1 receptor agonists (GLP-1 RAs), ie, dulaglutide and exenatide, and non–GLP-1 RA medications, ie, empagliflozin, sitagliptin, and glipizide. Results from databases that passed study diagnostics are provided, as well as the meta-analytic estimates.^b^ The total number of patients included is shown. CCAE indicates Merative MarketScan Commercial Claims and Encounters Database; Clinformatics, Optum’s deidentified Clinformatics Data Mart; IQVIA, IQVIA Open Claims; JHME, Johns Hopkins Medical Enterprise; MDCD, Merative MarketScan Multi-State Medicaid Database; MDCR, Merative MarketScan Medicare Supplemental and Coordination of Benefits Database; PharMetrics, PharMetrics Plus; VA, Department of Veterans Affairs; WashU, Washington University in St Louis. ^a^The specific NAION definition required 2 diagnoses. All other criteria were the same between the 2 definitions. ^b^Due to data privacy issues, databases that have counts less than 5 are unable to produce IRR estimates but still contribute to the meta-analytic estimate.

Up to 39 104 patients with NAION for the sensitive and 22 005 for the specific definitions contributed to this analysis. There was an increased NAION incidence rate associated with semaglutide exposure using both the sensitive (meta-analysis IRR, 1.32; 95% CI, 1.14-1.54; *P* < .001) (Figure 3) and the specific definitions (IRR, 1.50; 95% CI, 1.26-1.79; *P* < .001) (Figure 4). For exenatide, there was an increased incidence rate using the specific definition (IRR, 1.62; 95% CI, 1.02-2.58; *P* = .04) (Figure 4) but not the sensitive definition (IRR, 1.26; 95% CI, 0.78-2.03; *P* = .35) (Figure 3). There was no increased NAION incidence rate associated with dulaglutide or any non–GLP-1RA (Figure 3 and Figure 4).

## Discussion

In this retrospective study across 14 OHDSI network databases evaluating 37.1 million patients with T2D, the NAION incidence rate among new semaglutide users was 14.5 and 8.7 per 100 000 person-years (sensitive and specific NAION definitions, respectively). In the cohort analysis, we observed increased NAION risk comparing propensity score–matched new semaglutide users to users of non–GLP-1RA drugs empagliflozin and sitagliptin, depending on the NAION definition. In the self-control case-series analysis, we observed an increased NAION risk with semaglutide exposure relative to nonexposure; this finding was consistent across both NAION definitions. We also observed an increased NAION risk among patients exposed to exenatide, another GLP-1RA but only using the specific NAION definition. No increased risk was observed for the other GLP-1RA (dulaglutide), or non–GLP-1RA medications (empagliflozin, sitagliptin, glipizide).

Historically, the annual incidence of NAION has been reported as 2.3 to 11.4 per 100 000 persons among all patients depending on age group.^46,47,48,49,50,51^ However, some estimates have been as high as 82 per 100 000 persons, among individuals older than 68 years.^52^ The NAION incidence among patients with T2D in our study is within this range. Among new users of second-line T2D medications, our estimates for the NAION incidence proportion are much lower than estimates of 8.9% for semaglutide and 1.8% for non–GLP-1RAs from Hathaway et al.^4^ This is likely due to study population differences; Hathaway et al^4^ focused on neuro-ophthalmology patients at a major NAION referral center, whereas our population was from national databases across the US.

We created 2 definitions to address the lack of structured diagnostic codes specific to NAION. Manual records review suggests that 40% of cases coded as ischemic optic neuropathy are not actually NAION.^4^ Because manual review is not feasible for large database studies, we used 2 definitions of NAION developed through expert consensus to test the robustness of our findings against case misspecification. Because a definitive diagnosis often requires multiple visits, we improved specificity by requiring a second ischemic optic neuropathy diagnosis code in one of the definitions.^19,20,53,54^ Although the positive predictive value of the diagnosis codes can be enhanced with inclusion of the practitioner specialty, we were unable to include practitioner specialty due to limitations of the databases.^53^ The difference in the specificity of the 2 NAION definitions explains the variability in our estimated incidences.

Our study findings are largely consistent across the 2 analyses and NAION definitions. In the active-comparator new-user cohort analysis, the meta-analysis estimates of HRs ranged from 1.23 to 2.27 for new semaglutide users compared with non–GLP-1RA medications. The findings, however, were only in the comparisons with empagliflozin in the primary analysis and sitagliptin in a sensitivity analysis, both using the specific NAION definition. Case misspecification could be playing a role in the lack of statistical significance using the sensitive NAION definition. Although we did not adjust for multiple comparisons in the cohort analysis, findings were consistent in the self-controlled case series. We found an increase in NAION risks from semaglutide exposure compared with nonexposure, with an IRR of 1.32 and 1.50 using both specific and sensitive definitions. There is more statistical power in the self-controlled case series analysis since more databases passed study diagnostics and thus more patients were included.

Two prior studies^4,5^ have investigated NAION and semaglutide and have found contradictory findings. We do not know if the differences were due to variations in study design, patient population, statistical power, or some combination. The ability to assess how results vary across study designs and databases is an important feature and advantage of OHDSI network studies.^7,8,11,12,13,55^ Our self-controlled case-series findings for semaglutide also varied among databases—one-half the contributing databases did not demonstrate an increased IRR of NAION with semaglutide exposure. If our study had been performed on any of those databases alone, we would not have detected an increased IRR of NAION.

It is unclear if the increased risk of NAION is specific to semaglutide, or also true of other GLP-1RAs. In our study, we did not see an increased risk of NAION associated with dulaglutide. However, we did find a small increased risk of NAION with exenatide use when using the specific NAION definition. There are no known mechanistic links between GLP-1RA and NAION.^4^ On the contrary, GLP-1RAs have been associated with neuroprotective properties and reduction of ischemic risk.^56,57,58^ It is possible that GLP-1RAs affect vascular dynamics via the autonomic nervous system and lower systemic blood pressure, which could affect optic nerve head perfusion and increase NAION risk.^4,9,59,60^

### Strengths and Limitations

This study has some strengths. This was, to our knowledge, the largest study to investigate the association of NAION with semaglutide and other GLP-1RA and non–GLP-1RA T2D medications. Our sample size allowed us to identify many more NAION cases than prior studies. The diversity of databases allowed us to estimate incidence rates nationally. The rigorous study diagnostics addressed many biases and is another strength.

There were also some limitations with our study. Given lack of access to source records, we were unable to definitively distinguish incident cases of NAION from prevalent cases. However, the prior observation period in the cohort design and exclusion of the first year of observation in the self-controlled case series analysis increased the likelihood that the cases represented incident NAION. Some databases did not have patients older than 65 years; therefore, we could be missing cases of NAION as it often affects an older population. We were also unable to include cup-disc ratio data. Crowded optic nerves (ie, a small cup-disc ratio) is the strongest risk factor for NAION, outweighing systemic risk factors like diabetes.^61^ Incorporating ophthalmic examination data into the OMOP CDM is an active pursuit by the OHDSI Eye Care and Vision Research Workgroup.^62,63^ We were also unable to evaluate any heterogeneity of effects, such as whether semaglutide presents a greater risk for NAION among patients with anatomic predisposition or more severe diabetes (eg, by duration or hemoglobin A_1c_ level) or whether risk differs for patients who take semaglutide for obesity. In the OMOP CDM, drugs are identified by their active ingredients; the semaglutide group, therefore, included the subcutaneous injection (Ozempic [Novo Nordisk]) and by-mouth formulation (Rybelsus [Novo Nordisk]). The subcutaneous form of semaglutide has greater bioavailability, shows greater efficacy in glucose lowering, and is more commonly prescribed.^35,64^ Even with the inclusion of a less effective semaglutide formulation we were able to identify an increased risk of NAION. We were also unable to include drug dose in the analysis. Time-varying confounding in the self-controlled case series is a possible concern. Diabetes worsens over time with aging, increasing likelihood of both being prescribed T2D medications and of developing NAION. However, this should affect the analysis of all T2D medications; the higher estimates of NAION risk under semaglutide relative to other medications suggests our finding not to be an artifact of such time-varying confounding. Patients with T2D who develop coronary artery disease are more likely to be prescribed semaglutide, contributing to potential indication bias because coronary artery disease is also an NAION risk factor.^65^ To alleviate this concern, we excluded databases where there was a higher NAION incidence during the 30-day preexposure period, which would suggest such indication bias. Databases showing substantial systematic error on the negative control outcomes, expressed as an absolute expected systematic error greater than 0.25, were also excluded.

## Conclusions

In conclusion, in this retrospective study, we identified a small increased risk of NAION among patients with T2D who were exposed to semaglutide, smaller than that previously reported.^4^ Additional studies that incorporate ophthalmic risk factors or examine dose-dependent effects, eg, are needed to investigate a potential causal relationship between semaglutide and NAION. In the absence of a known mechanism for this association, we urge clinicians to weigh the concern for an increased risk of a rare but potentially blinding eye condition with the many therapeutic benefits of semaglutide.

## Notes

Supplement 1. **eTable 1.** Description of the Databases (Administrative Claims and Electronic Health Records) Included in the Study **eTable 2.** Details of the Indication Cohort (Patients With T2DM), Exposure Cohorts (Semaglutide, Dulaglutide, Exenatide, Empagliflozin, Sitagliptin, Glipizide), and Outcome Cohorts (Sensitive and Specific Definitions of NAION) **eTable 3.** Codes From Standardized Vocabularies Used to Define Clinical Concepts **eTable 5.** Results From the Study Diagnostics for the New-User Active-Comparator Cohort Design Analysis for the “Sensitive” NAION Definition **eTable 6.** Results From the Study Diagnostics for the New-User Active-Comparator Cohort Design Analysis for the “Specific” NAION Definition **eTable 7.** Results from the Study Diagnostics for the Self-Controlled Case-Series Analysis for the “Sensitive” NAION Definition **eTable 8.** Results From the Study Diagnostics for the Self-Controlled Case-Series Analysis for the “Specific” NAION Definition **eTable 9.** Performance Characteristics of Definition Algorithms for the “Sensitive” and “Specific” Definitions of NAION **eTable 10.** Baseline Characteristics of Patients in Each Exposure Cohort Semaglutide, Dulaglutide, Exenatide, Empagliflozin, Sitagliptin, Glipizide During the Study Period in All Databases **eTable 11.** Incidence Proportion and Incidence Rate of NAION Among Adults With T2DM and in Each T2DM Drug Exposure Cohort (Semaglutide, Dulaglutide, Exenatide, Empagliflozin, Sitagliptin, Glipizide) Across All Databases **eFigure 1.** Kaplan-Meier Plot Showing Survival as a Function of Time Comparing New Users of Semaglutide and Dulaglutide Using the “Sensitive” NAION Definition **eFigure 2.** Kaplan-Meier Plot Showing Survival as a Function of Time Comparing New Users of Semaglutide and Empagliflozin Using the “Sensitive” NAION Definition **eFigure 3.** Forest Plot for Active-Comparator Cohort Design, Results From the First Sensitivity Analysis (Not Requiring Second-Line Treatment) **eFigure 4.** Forest Plot for Active-Comparator Cohort Design, Results From the Second Sensitivity Analysis (Restriction by Calendar Time)

Supplement 2. **eTable 4.** Nonstandard OMOP to Standard OMOP Concept Mapping

Supplement 3.
Data Sharing Statement.

## Notes

Journal Club Slides
