# Supplementary material for: Semaglutide and Nonarteritic Anterior Ischemic Optic Neuropathy
Source: JAMA Ophthalmol. 2025 Feb 20;143(4):304–14. doi: 10.1001/jamaophthalmol.2024.6555 (PMC11843465; doi:10.1001/jamaophthalmol.2024.6555)
Supplement: Supplement 1. — eTable 1. Description of the Databases (Administrative Claims and Electronic Health Records) Included in the Study eTable 2. Details of the Indication Cohort (Patients With T2DM), Exposure Cohorts (Semaglutide, Dulaglutide, Exenatide, Empagliflozin, Sitagliptin, Glipizide), and Outcome Cohorts (Sensitive and Specific Definitions of NAION) eTable 3. Codes From Standardized Vocabularies Used to Define Clinical Concepts eTable 5. Results From the Study Diagnostics for the New-User Active-Comparator Cohort Design Analysis for the “Sensitive” NAION Definition eTable 6. Results From the Study Diagnostics for the New-User Active-Comparator Cohort Design Analysis for the “Specific” NAION Definition eTable 7. Results from the Study Diagnostics for the Self-Controlled Case-Series Analysis for the “Sensitive” NAION Definition eTable 8. Results From the Study Diagnostics for the Self-Controlled Case-Series Analysis for the “Specific” NAION Definition eTable 9. Performance Characteristics of Definition Algorithms for the “Sensitive” and “Specific” Definitions of NAION eTable 10. Baseline Characteristics of Patients in Each Exposure Cohort Semaglutide, Dulaglutide, Exenatide, Empagliflozin, Sitagliptin, Glipizide During the Study Period in All Databases eTable 11. Incidence Proportion and Incidence Rate of NAION Among Adults With T2DM and in Each T2DM Drug Exposure Cohort (Semaglutide, Dulaglutide, Exenatide, Empagliflozin, Sitagliptin, Glipizide) Across All Databases eFigure 1. Kaplan-Meier Plot Showing Survival as a Function of Time Comparing New Users of Semaglutide and Dulaglutide Using the “Sensitive” NAION Definition eFigure 2. Kaplan-Meier Plot Showing Survival as a Function of Time Comparing New Users of Semaglutide and Empagliflozin Using the “Sensitive” NAION Definition eFigure 3. Forest Plot for Active-Comparator Cohort Design, Results From the First Sensitivity Analysis (Not Requiring Second-Line Treatment) eFigure 4. Forest Plot for Active-Comparator Cohort Design, R [file jamaophthalmol-e246555-s001.pdf]

Supplementary Online Content

Cai CX, Hribar M, Baxter S, et al. Semaglutide and nonarteritic anterior ischemic optic neuropathy. *JAMA Ophthalmol.* Published online February 20, 2025. doi:10.1001/jamaophthalmol.2024.6555

eTable 1. Description of the Databases (Administrative Claims and Electronic Health Records) Included in the Study
eTable 2. Details of the Indication Cohort (Patients With T2DM), Exposure Cohorts (Semaglutide, Dulaglutide, Exenatide, Empagliflozin, Sitagliptin, Glipizide), and Outcome Cohorts (Sensitive and Specific Definitions of NAION)
eTable 3. Codes From Standardized Vocabularies Used to Define Clinical Concepts
eTable 5. Results From the Study Diagnostics for the New-User Active-Comparator Cohort Design Analysis for the “Sensitive” NAION Definition
eTable 6. Results From the Study Diagnostics for the New-User Active-Comparator Cohort Design Analysis for the “Specific” NAION Definition
eTable 7. Results from the Study Diagnostics for the Self-Controlled Case-Series Analysis for the “Sensitive” NAION Definition
eTable 8. Results From the Study Diagnostics for the Self-Controlled Case-Series Analysis for the “Specific” NAION Definition
eTable 9. Performance Characteristics of Definition Algorithms for the “Sensitive” and “Specific” Definitions of NAION
eTable 10. Baseline Characteristics of Patients in Each Exposure Cohort Semaglutide, Dulaglutide, Exenatide, Empagliflozin, Sitagliptin, Glipizide During the Study Period in All Databases
eTable 11. Incidence Proportion and Incidence Rate of NAION Among Adults With T2DM and in Each T2DM Drug Exposure Cohort (Semaglutide, Dulaglutide, Exenatide, Empagliflozin, Sitagliptin, Glipizide) Across All Databases
eFigure 1. Kaplan-Meier Plot Showing Survival as a Function of Time Comparing New Users of Semaglutide and Dulaglutide Using the “Sensitive” NAION Definition
eFigure 2. Kaplan-Meier Plot Showing Survival as a Function of Time Comparing New Users of Semaglutide and Empagliflozin Using the “Sensitive” NAION Definition
eFigure 3. Forest Plot for Active-Comparator Cohort Design, Results From the First Sensitivity Analysis (Not Requiring Second-Line Treatment)
eFigure 4. Forest Plot for Active-Comparator Cohort Design, Results From the Second Sensitivity Analysis (Restriction by Calendar Time)

This supplementary material has been provided by the authors to give readers additional information about their work.

© 2025 Cai CX et al. *JAMA Ophthalmology.*

eTable 1: Description of the databases (administrative claims and electronic health records) included in the study.

Data Source	Population	Patients (millions)	History	Data Capture Process and Short Description	Included in Which Component of Analysis*
Merative MarketScan Commercial Claims and Encounters Database (CCAE)	USA, commercially insured, <65 years	172	2000-2024	The Merative(R) MarketScan(R) Commercial Database (CCAE) includes health insurance claims across the continuum of care (e.g. inpatient, outpatient, outpatient pharmacy, carve-out behavioral healthcare) as well as enrollment data from large employers and health plans across the United States who provide private healthcare coverage for employees, their spouses, and dependents. This administrative claims database includes a variety of fee- for-service, preferred provider organizations, and capitated health plans.	Characterization, cohort method, SCCS
Optum's de-identified Clinformatics Data Mart Database (Clinformatics)	USA, general	99	2000-2023	Optum's Clinformatics Data Mart (CDM) is derived from a database of administrative health claims for members of large commercial and Medicare Advantage health plans. Clinformatics Data Mart is statistically de-identified under the Expert Determination method consistent with HIPAA and managed according to Optum customer data use agreements. CDM administrative claims submitted for payment by providers and pharmacies are verified, adjudicated and de-identified prior to inclusion. This data,	Characterization, cohort method, SCCS

© 2025 Cai CX et al. JAMA Ophthalmology.

				including patient-level enrollment information, is derived from claims submitted for all medical and pharmacy health care services with information related to health care costs and resource utilization. The population is geographically diverse, spanning all 50 states.	
Columbia University Medical Center (CUMC)	USA, general	6	1985-2024	Non-profit academic medical center	Characterization
IQVIA Open Claims (IQVIA)	USA, general	325.5	2000-2024	A United States database of open, pre-adjudicated claims. Data are reported at anonymized patient level collected from office-based physicians and specialists via office management software and clearinghouse switch resources for the purpose of reimbursement. A subset of medical claims data have adjudicated claims. Number list represents active patients.	Characterization, cohort method, SCCS
Johns Hopkins Medical Enterprise (JHME)	USA, general	2.15	2016-2024	Non-profit academic medical center covering 6 hospitals and numerous outpatient facilities.	Characterization, SCCS
Merative MarketScan Multi-State Medicaid Database (MDCD)	USA, Medicaid enrollees, racially diverse	36	2006- June 2023	The Merative(R) MarketScan(R) Multi-State Medicaid Database (MDCD) reflects the healthcare service use of individuals covered by Medicaid programs in numerous geographically dispersed states. The database contains the pooled healthcare experience of Medicaid enrollees, covered under fee-for-service and managed care plans. It includes records of	Characterization, cohort method, SCCS

© 2025 Cai CX et al. JAMA Ophthalmology.

				inpatient services, inpatient admissions, outpatient services, and prescription drug claims, as well as information on long-term care. Data on eligibility and service and provider type are also included. In addition to standard demographic variables such as age and gender, the database includes variables such as federal aid category (income based, disability, Temporary Assistance for Needy Families) and race.	
Merative MarketScan Medicare Supplemental and Coordination of Benefits Database (MDCR)	USA, commercially insured, 65+ years	11	2000- June 2023	The Merative(R) MarketScan(R) Medicare Supplemental Database (MDCR) represents the health services of retirees in the United States with Medicare supplemental coverage through employer-sponsored plans. This database contains primarily fee-for-service plans and includes health insurance claims across the continuum of care (e.g. inpatient, outpatient and outpatient pharmacy).	Characterization, cohort method, SCCS
Oregon Health & Science University (OHSU)	USA, general	4.68	2006-2024	Non-profit academic medical center	Characterization
Optum de-identified Electronic Health Record data set (Optum EHR)	USA, general	114	2007-2024	Optum’s longitudinal EHR repository is derived from dozens of healthcare provider organizations in the United States. The data is certified as de-identified by an independent statistical expert following HIPAA statistical de-identification rules and managed according to Optum customer data use agreements . Clinical,	Characterization, cohort method

© 2025 Cai CX et al. JAMA Ophthalmology.

				claims and other medical administrative data is obtained from both Inpatient and Ambulatory electronic health records (EHRs), practice management systems and numerous other internal systems. Information is processed, normalized, and standardized across the continuum of care from both acute inpatient stays and outpatient visits. Optum data elements include demographics, medications prescribed and administered, immunizations, allergies, lab results (including microbiology), vital signs and other observable measurements, clinical and inpatient stay administrative data and coded diagnoses and procedures. In addition, Optum uses natural language processing (NLP) computing technology to transform critical facts from physician notes into usable datasets. The NLP data provides detailed information regarding signs and symptoms, family history, disease related scores (i.e. RAPID3 for RA, or CHADS2 for stroke risk), genetic testing, medication changes, and physician rationale behind prescribing decisions that might never be recorded in the EHR.	
PharMetrics Plus (PharMetrics)	USA, commercially insured, <65 years	156	2017-October 2023	The IQVIA™ Adjudicated Health Plan Claims Data (formerly PharMetrics Plus) - US database is comprised of fully adjudicated health plan	Characterization, cohort method, SCCS

© 2025 Cai CX et al. JAMA Ophthalmology.

				claims data and enrollment information for commercial individuals. The information is comprised of over 70 contributing health plans and self-insured employer groups throughout the United States over the last 5 years. This anonymous, patient-centric database includes all medical and pharmacy claims data (costs and descriptive services). Claims represent payments to providers for services rendered to covered health plan individuals. The data also includes patient-level enrollment which is a record of demographic variables including eligibility status (YOB, gender, US Census region, eligibility by month). The enrollee population in the database is generally representative of the <65 years of age, commercially insured population with a subset of Commercial Medicare and Medicaid in the US with respect to both age and gender. The average length of enrollment is ≥ 39 months and ≥ 47 million patients have 3 or more years of continuous enrollment (medical and pharmacy coverage). Each contributing plan's data undergoes rigorous data quality review by IQVIA™ prior to its addition to the IQVIA™ Adjudicated Health Plan Claims - US	

© 2025 Cai CX et al. JAMA Ophthalmology.

				database.	
Stanford University (STARR)	USA, general	4	2008-2024	The **STA**nford Medicine **R**esearch Data **R**epository OMOP (STARR-OMOP) is Stanford's second-generation clinical data warehouse dataset designed to enhance access to healthcare data for research purposes. Launched in 2019, STARR-OMOP contains electronic health records data from Stanford Health Care and Stanford Children's Hospital, the TriValley Hospital, and associated clinics for around 4 million patients from 2008. The dataset is refreshed monthly and contains demographics, labs, diagnoses, drugs, and procedure information, as well as clinical notes. Several flowsheet fields are also mapped to the OMOP measurements table, including vitals such as blood pressure, oxygen level, heart rate, respiratory rate, measurements from the Sequential Organ Failure Assessment (SOFA) score, etc. The structured and unstructured data is anonymized using a combination of Safe Harbor and other techniques. Based on guidelines from our University Privacy Office (UPO), our location data contains zip5 data for	Characterization, SCCS

© 2025 Cai CX et al. JAMA Ophthalmology.

				over 77% of the population. Our data and ETL processes are entirely hosted on the cloud.	
Keck Medical Center of University of Southern California (USC)	USA, general	1	2013-2024	Non-profit academic medical center	Characterization
Department of Veterans Affairs (VA)	USA, veterans, older, racially diverse	18.2	2000-2024	National VA healthcare system, the largest integrated provider of medical services in the USA, providing care at 170 VA medical centers and 1063 outpatients facilities	Characterization, cohort method, SCCS
Washington University (WashU)	USA, general	2	2000-2024	Non-profit academic medical center	Characterization, SCCS

* Characterization (calculation of incidence proportion and incidence rate of NAION), cohort method = active-comparator new-user cohort design, SCCS = self-controlled case-series method

© 2025 Cai CX et al. JAMA Ophthalmology.

eTable 2: Details of the indication cohort (patients with T2DM), exposure cohorts (semaglutide, dulaglutide, exenatide, empagliflozin, sitagliptin, glipizide), and outcome cohorts (sensitive and specific definitions of NAION). The codes used to define the items appearing in square brackets can be found in eTable 3.

Cohort	Cohort Entry Event	Additional Inclusion Criteria	Cohort Exit
T2DM	People may enter the cohort when observing any of the following:<br>1. condition occurrences of [Type 2 diabetes mellitus].<br>Limit cohort entry events to the earliest event per person.<br>Restrict entry events to with all of the following criteria:<br>1. having no condition occurrences of [Type 1 diabetes mellitus], starting anytime on or before cohort entry start date; allow events outside observation period.<br>2. having no condition occurrences of [Secondary diabetes mellitus], starting anytime on or before cohort entry start date; allow events outside observation period.		The person also exits the cohort at the end of continuous observation.
New user of [GLP-1 receptor agonists drug of interest]* as 2nd-line treatment with prior T2DM and prior metformin	People with continuous observation of 365 days before event may enter the cohort when observing any of the following:<br>1. drug exposure of [GLP-1 receptor agonists drug of interest]* for the first time in the person's history.<br>Limit cohort entry events to the earliest event per person.<br>Restrict entry events to with all of the following criteria:<br>1. with the following event criteria: who are >= 18 years old.<br>2. having at least 1 condition occurrence of [Type 2 diabetes mellitus], starting anytime on or before cohort entry start date; allow events outside observation period.<br>3. having no condition occurrences of [Type 1 diabetes mellitus], starting anytime on or before cohort entry start date; allow events outside observation period.	I. Prior metformin use<br>Entry events with any of the following criteria:<br>1. having at least 1 drug era of [Metformin], starting anytime up to 90 days before cohort entry start date; allow events outside observation period; with era length >= 90 days.<br>2. having at least 3 drug exposures of [Metformin], starting anytime on or before cohort entry start date; allow events outside observation period.<br>II. No prior GLP-1 RA exposure<br>Entry events having no drug exposures of [GLP-1 receptor agonists excluding the drug of interest], starting anytime on or before cohort entry start date; allow events outside observation period.<br>III. No prior DPP4 inhibitor exposure<br>Entry events having no drug exposures of [DPP4 inhibitors], starting anytime on or before cohort	The cohort end date will be based on a continuous exposure to [GLP-1 receptor agonists drug of interest]*: allowing 30 days between exposures, adding 0 days after exposure ends, and using days supply and exposure end date for exposure duration.

© 2025 Cai CX et al. JAMA Ophthalmology.

	4. having no condition occurrences of [Secondary diabetes mellitus], starting anytime on or before cohort entry start date; allow events outside observation period.	entry start date; allow events outside observation period.<br>IV. No prior SGLT-2 inhibitor exposure<br>Entry events having no drug exposures of [SGLT2 inhibitors], starting anytime on or before cohort entry start date; allow events outside observation period.<br>V. No prior SU exposure<br>Entry events having no drug exposures of [Sulfonylureas], starting anytime on or before cohort entry start date; allow events outside observation period.<br>VI. No prior other anti-diabetic exposure<br>Entry events having no drug exposures of [Other anti-diabetics], starting anytime on or before cohort entry start date; allow events outside observation period.<br>VII. No prior insulin use or combo initiation: Proxy for < 30 days drug era anytime before index and no combination use on index<br>Entry events with all of the following criteria:<br>1. having no drug eras of [Insulin], starting anytime up to 30 days before cohort entry start date; allow events outside observation period; with era length > 30 days.<br>2. having no drug eras of [Insulin], starting between 30 days before and 0 days after cohort entry start date; allow events outside observation period.	
New user of [empagliflozin] as 2nd-line treatment with prior T2DM and prior metformin	People with continuous observation of 365 days before event may enter the cohort when observing any of the following:<br>1. drug exposure of [empagliflozin] for the first time in the person's history.<br>Limit cohort entry events to the earliest event per person.<br>Restrict entry events to with all of the following criteria:<br>1. with the following event	I. Prior metformin use<br>Entry events with any of the following criteria:<br>1. having at least 1 drug era of [Metformin], starting anytime up to 90 days before cohort entry start date; allow events outside observation period; with era length >= 90 days.<br>2. having at least 3 drug	The cohort end date will be based on a continuous exposure to [empagliflozin]: allowing 30 days between exposures, adding 0 days after exposure ends, and

© 2025 Cai CX et al. JAMA Ophthalmology.

	criteria: who are >= 18 years old.<br>2. having at least 1 condition occurrence of [Type 2 diabetes mellitus], starting anytime on or before cohort entry start date; allow events outside observation period.<br>3. having no condition occurrences of [Type 1 diabetes mellitus], starting anytime on or before cohort entry start date; allow events outside observation period.<br>4. having no condition occurrences of [Secondary diabetes mellitus], starting anytime on or before cohort entry start date; allow events outside observation period.	exposures of [Metformin], starting anytime on or before cohort entry start date; allow events outside observation period.<br>II. No prior GLP-1 RA exposure<br>Entry events having no drug exposures of [GLP-1 receptor agonists], starting anytime on or before cohort entry start date; allow events outside observation period.<br>III. No prior DPP4 inhibitor exposure<br>Entry events having no drug exposures of [DPP4 inhibitors], starting anytime on or before cohort entry start date; allow events outside observation period.<br>IV. No prior SGLT-2 inhibitor exposure<br>Entry events having no drug exposures of [empagliflozin], starting anytime on or before cohort entry start date; allow events outside observation period.<br>V. No prior SU exposure<br>Entry events having no drug exposures of [Sulfonylureas], starting anytime on or before cohort entry start date; allow events outside observation period.<br>VI. No prior other anti-diabetic exposure<br>Entry events having no drug exposures of [Other anti-diabetics], starting anytime on or before cohort entry start date; allow events outside observation period.<br>VII. No prior insulin use or combo initiation: Proxy for < 30 days drug era anytime before index and no combination use on index<br>Entry events with all of the following criteria:<br>1. having no drug eras of [Insulin], starting anytime up to 30 days before cohort entry start date; allow events outside observation period; with era length > 30 days.	using days supply and exposure end date for exposure duration.

© 2025 Cai CX et al. JAMA Ophthalmology.

		2. having no drug eras of [Insulin], starting between 30 days before and 0 days after cohort entry start date; allow events outside observation period.	
New user of [sitagliptin] as 2nd-line treatment with prior T2DM and prior metformin	People with continuous observation of 365 days before event may enter the cohort when observing any of the following:<br>1. drug exposure of [sitagliptin] for the first time in the person's history.<br>Limit cohort entry events to the earliest event per person.<br>Restrict entry events to with all of the following criteria:<br>1. with the following event criteria: who are >= 18 years old.<br>2. having at least 1 condition occurrence of [Type 2 diabetes mellitus], starting anytime on or before cohort entry start date; allow events outside observation period.<br>3. having no condition occurrences of [Type 1 diabetes mellitus], starting anytime on or before cohort entry start date; allow events outside observation period.<br>4. having no condition occurrences of [Secondary diabetes mellitus], starting anytime on or before cohort entry start date; allow events outside observation period.	I. Prior metformin use<br>Entry events with any of the following criteria:<br>1. having at least 1 drug era of [Metformin], starting anytime up to 90 days before cohort entry start date; allow events outside observation period; with era length >= 90 days.<br>2. having at least 3 drug exposures of [Metformin], starting anytime on or before cohort entry start date; allow events outside observation period.<br>II. No prior GLP-1 RA exposure<br>Entry events having no drug exposures of [GLP-1 receptor agonists], starting anytime on or before cohort entry start date; allow events outside observation period.<br>III. No prior DPP4 inhibitor exposure<br>Entry events having no drug exposures of [DPP4 inhibitors except sitagliptin], starting anytime on or before cohort entry start date; allow events outside observation period.<br>IV. No prior SGLT-2 inhibitor exposure<br>Entry events having no drug exposures of [SGLT2 inhibitors], starting anytime on or before cohort entry start date; allow events outside observation period.<br>V. No prior SU exposure<br>Entry events having no drug exposures of [Sulfonylureas], starting anytime on or before cohort entry start date; allow events outside observation period.<br>VI. No prior other anti-diabetic	The cohort end date will be based on a continuous exposure to [sitagliptin]: allowing 30 days between exposures, adding 0 days after exposure ends, and using days supply and exposure end date for exposure duration.

© 2025 Cai CX et al. JAMA Ophthalmology.

		exposure<br>Entry events having no drug exposures of [Other anti-diabetics], starting anytime on or before cohort entry start date; allow events outside observation period.<br>VII. No prior insulin use or combo initiation: Proxy for < 30 days drug era anytime before index and no combination use on index<br>Entry events with all of the following criteria:<br>1. having no drug eras of [Insulin], starting anytime up to 30 days before cohort entry start date; allow events outside observation period; with era length > 30 days.<br>2. having no drug eras of [Insulin], starting between 30 days before and 0 days after cohort entry start date; allow events outside observation period.	
New user of [glipizide] as 2nd-line treatment with prior T2DM and prior metformin	People with continuous observation of 365 days before event may enter the cohort when observing any of the following:<br>1. drug exposure of [glipizide] for the first time in the person's history.<br>Limit cohort entry events to the earliest event per person.<br>Restrict entry events to with all of the following criteria:<br>1. with the following event criteria: who are >= 18 years old.<br>2. having at least 1 condition occurrence of [Type 2 diabetes mellitus], starting anytime on or before cohort entry start date; allow events outside observation period.<br>3. having no condition occurrences of [Type 1 diabetes mellitus], starting anytime on or before cohort entry start date; allow events outside observation period.	I. Prior metformin use<br>Entry events with any of the following criteria:<br>1. having at least 1 drug era of [Metformin], starting anytime up to 90 days before cohort entry start date; allow events outside observation period; with era length >= 90 days.<br>2. having at least 3 drug exposures of [Metformin], starting anytime on or before cohort entry start date; allow events outside observation period.<br>II. No prior GLP-1 RA exposure<br>Entry events having no drug exposures of [GLP-1 receptor agonists], starting anytime on or before cohort entry start date; allow events outside observation period.<br>III. No prior DPP4 inhibitor exposure<br>Entry events having no drug	The cohort end date will be based on a continuous exposure to [glipizide]: allowing 30 days between exposures, adding 0 days after exposure ends, and using days supply and exposure end date for exposure duration.

© 2025 Cai CX et al. JAMA Ophthalmology.

	4. having no condition occurrences of [Secondary diabetes mellitus], starting anytime on or before cohort entry start date; allow events outside observation period.	exposures of [DPP4 inhibitors], starting anytime on or before cohort entry start date; allow events outside observation period.<br>IV. No prior SGLT-2 inhibitor exposure<br>Entry events having no drug exposures of [SGLT2 inhibitors], starting anytime on or before cohort entry start date; allow events outside observation period.<br>V. No prior SU exposure<br>Entry events having no drug exposures of [Sulfonylureas except glipizide], starting anytime on or before cohort entry start date; allow events outside observation period.<br>VI. No prior other anti-diabetic exposure<br>Entry events having no drug exposures of [Other anti-diabetics], starting anytime on or before cohort entry start date; allow events outside observation period.<br>VII. No prior insulin use or combo initiation: Proxy for < 30 days drug era anytime before index and no combination use on index<br>Entry events with all of the following criteria:<br>1. having no drug eras of [Insulin], starting anytime up to 30 days before cohort entry start date; allow events outside observation period; with era length > 30 days.<br>2. having no drug eras of [Insulin], starting between 30 days before and 0 days after cohort entry start date; allow events outside observation period.	
New user of [GLP-1 receptor agonists drug of interest]* with prior T2DM and prior metformin and no insulin	People with continuous observation of 365 days before event may enter the cohort when observing any of the following:<br>1. drug exposure of [GLP-1 receptor agonists drug of interest]* for the first time in	I. Prior metformin use<br>Entry events with any of the following criteria:<br>1. having at least 1 drug era of [Metformin], starting anytime up to 90 days	The cohort end date will be based on a continuous exposure to [GLP-1 receptor

© 2025 Cai CX et al. JAMA Ophthalmology.

	the person's history.<br>Limit cohort entry events to the earliest event per person.<br>Restrict entry events to with all of the following criteria:<br>1. with the following event criteria: who are >= 18 years old.<br>2. having at least 1 condition occurrence of [Type 2 diabetes mellitus], starting anytime on or before cohort entry start date; allow events outside observation period.<br>3. having no condition occurrences of [Type 1 diabetes mellitus], starting anytime on or before cohort entry start date; allow events outside observation period.<br>4. having no condition occurrences of [Secondary diabetes mellitus], starting anytime on or before cohort entry start date; allow events outside observation period.	before cohort entry start date; allow events outside observation period; with era length >= 90 days.<br>2. having at least 3 drug exposures of [Metformin], starting anytime on or before cohort entry start date; allow events outside observation period.<br>II. No prior insulin use or combo initiation: Proxy for < 30 days drug era anytime before index and no combination use on index<br>Entry events with all of the following criteria:<br>1. having no drug eras of [Insulin], starting anytime up to 30 days before cohort entry start date; allow events outside observation period; with era length > 30 days.<br>2. having no drug eras of [Insulin], starting between 30 days before and 0 days after cohort entry start date; allow events outside observation period.	agonists drug of interest]*: allowing 30 days between exposures, adding 0 days after exposure ends, and using days supply and exposure end date for exposure duration.
New user of empagliflozin with prior T2DM and prior metformin and no insulin	People with continuous observation of 365 days before event may enter the cohort when observing any of the following:<br>1. drug exposure of [empagliflozin] for the first time in the person's history.<br>Limit cohort entry events to the earliest event per person.<br>Restrict entry events to with all of the following criteria:<br>1. with the following event criteria: who are >= 18 years old.<br>2. having at least 1 condition occurrence of [Type 2 diabetes mellitus], starting anytime on or before cohort entry start date; allow events outside observation period.<br>3. having no condition	I. Prior metformin use<br>Entry events with any of the following criteria:<br>1. having at least 1 drug era of [Metformin], starting anytime up to 90 days before cohort entry start date; allow events outside observation period; with era length >= 90 days.<br>2. having at least 3 drug exposures of [Metformin], starting anytime on or before cohort entry start date; allow events outside observation period.<br>II. No prior insulin use or combo initiation: Proxy for < 30 days drug era anytime before index and no	The cohort end date will be based on a continuous exposure to [empagliflozin]: allowing 30 days between exposures, adding 0 days after exposure ends, and using days supply and exposure end date for exposure duration.

© 2025 Cai CX et al. JAMA Ophthalmology.

	occurrences of [Type 1 diabetes mellitus], starting anytime on or before cohort entry start date; allow events outside observation period.<br>4. having no condition occurrences of [Secondary diabetes mellitus], starting anytime on or before cohort entry start date; allow events outside observation period.	combination use on index<br>Entry events with all of the following criteria:<br>1. having no drug eras of [Insulin], starting anytime up to 30 days before cohort entry start date; allow events outside observation period; with era length > 30 days.<br>2. having no drug eras of [Insulin], starting between 30 days before and 0 days after cohort entry start date; allow events outside observation period.	
New user of sitagliptin with prior T2DM and prior metformin and no insulin	People with continuous observation of 365 days before event may enter the cohort when observing any of the following:<br>1. drug exposure of [sitagliptin] for the first time in the person's history.<br>Limit cohort entry events to the earliest event per person.<br>Restrict entry events to with all of the following criteria:<br>1. with the following event criteria: who are >= 18 years old.<br>2. having at least 1 condition occurrence of [Type 2 diabetes mellitus], starting anytime on or before cohort entry start date; allow events outside observation period.<br>3. having no condition occurrences of [Type 1 diabetes mellitus], starting anytime on or before cohort entry start date; allow events outside observation period.<br>4. having no condition occurrences of [Secondary diabetes mellitus], starting anytime on or before cohort entry start date; allow events outside observation period.	I. Prior metformin use<br>Entry events with any of the following criteria:<br>1. having at least 1 drug era of [Metformin], starting anytime up to 90 days before cohort entry start date; allow events outside observation period; with era length >= 90 days.<br>2. having at least 3 drug exposures of [Metformin], starting anytime on or before cohort entry start date; allow events outside observation period.<br>II. No prior insulin use or combo initiation: Proxy for < 30 days drug era anytime before index and no combination use on index<br>Entry events with all of the following criteria:<br>1. having no drug eras of [Insulin], starting anytime up to 30 days before cohort entry start date; allow events outside observation period; with era length > 30 days.<br>2. having no drug eras of [Insulin], starting between 30 days before and 0 days	The cohort end date will be based on a continuous exposure to [sitagliptin]: allowing 30 days between exposures, adding 0 days after exposure ends, and using days supply and exposure end date for exposure duration.

© 2025 Cai CX et al. JAMA Ophthalmology.

		after cohort entry start date; allow events outside observation period.	
New user of glipizide with prior T2DM and prior metformin and no insulin	People with continuous observation of 365 days before event may enter the cohort when observing any of the following:<br>1. drug exposure of [glipizide] for the first time in the person's history.<br>Limit cohort entry events to the earliest event per person.<br>Restrict entry events to with all of the following criteria:<br>1. with the following event criteria: who are >= 18 years old.<br>2. having at least 1 condition occurrence of [Type 2 diabetes mellitus], starting anytime on or before cohort entry start date; allow events outside observation period.<br>3. having no condition occurrences of [Type 1 diabetes mellitus], starting anytime on or before cohort entry start date; allow events outside observation period.<br>4. having no condition occurrences of [Secondary diabetes mellitus], starting anytime on or before cohort entry start date; allow events outside observation period.	I. Prior metformin use<br>Entry events with any of the following criteria:<br>1. having at least 1 drug era of [Metformin], starting anytime up to 90 days before cohort entry start date; allow events outside observation period; with era length >= 90 days.<br>2. having at least 3 drug exposures of [Metformin], starting anytime on or before cohort entry start date; allow events outside observation period.<br>II. No prior insulin use or combo initiation: Proxy for < 30 days drug era anytime before index and no combination use on index<br>Entry events with all of the following criteria:<br>1. having no drug eras of [Insulin], starting anytime up to 30 days before cohort entry start date; allow events outside observation period; with era length > 30 days.<br>2. having no drug eras of [Insulin], starting between 30 days before and 0 days after cohort entry start date; allow events outside observation period.	The cohort end date will be based on a continuous exposure to [glipizide]: allowing 30 days between exposures, adding 0 days after exposure ends, and using days supply and exposure end date for exposure duration.
New user of semaglutide as 2nd-line treatment with prior T2DM Dec2017-Jan2020	Same as New user of GLP-1 receptor agonists drug of interest (= semaglutide) with prior T2DM and prior metformin and no insulin<br>AND Additional Inclusion Criteria<br>VIII. index date between 1Dec2017 and 31Jan2020<br>Entry events with the following event criteria: starting between December 1, 2017 and January 31, 2020.		
New user of semaglutide as	Same as New user of GLP-1 receptor agonists drug of interest (= semaglutide) with prior T2DM and prior metformin and no insulin		

© 2025 Cai CX et al. JAMA Ophthalmology.

2nd-line treatment with prior T2DM Feb2020-Jun2021	AND Additional Inclusion Criteria<br>VIII. index date between 1Feb2020 and 30Jun2021<br>Entry events with the following event criteria: starting between February 1, 2020 and June 30, 2021.		
New user of semaglutide as 2nd-line treatment with prior T2DM Jul2021-Dec2023	Same as New user of GLP-1 receptor agonists drug of interest (= semaglutide) with prior T2DM and prior metformin and no insulin<br>AND Additional Inclusion Criteria<br>VIII. index date between 1Jul2021 and 31Dec2023<br>Entry events with the following event criteria: starting between July 1, 2021 and December 31, 2023.		
New user of empagliflozin as 2nd-line treatment with prior T2DM Dec2017-Jan2020	Same as New user of empagliflozin with prior T2DM and prior metformin and no insulin<br>AND Additional Inclusion Criteria<br>VIII. index date between 1Dec2017 and 31Jan2020<br>Entry events with the following event criteria: starting between December 1, 2017 and January 31, 2020.		
New user of empagliflozin as 2nd-line treatment with prior T2DM Feb2020-Jun2021	Same as New user of empagliflozin with prior T2DM and prior metformin and no insulin<br>AND Additional Inclusion Criteria<br>VIII. index date between 1Feb2020 and 30Jun2021<br>Entry events with the following event criteria: starting between February 1, 2020 and June 30, 2021.		
New user of empagliflozin as 2nd-line treatment with prior T2DM Jul2021-Dec2023	Same as New user of empagliflozin with prior T2DM and prior metformin and no insulin<br>AND Additional Inclusion Criteria<br>VIII. index date between 1Jul2021 and 31Dec2023<br>Entry events with the following event criteria: starting between July 1, 2021 and December 31, 2023.		
“Sensitive” NAION	People may enter the cohort when observing any of the following:<br>1. condition occurrences of [Nonarteritic Anterior Ischemic Optic Neuropathy].<br>2. condition occurrences of [presention conditons for index date correction].	I. no giant cell arteritis (defined by 2x diagnosis)<br>Entry events with at most 0 of the following criteria:<br>1. having at least 2 distinct start dates from condition occurrences of [Giant cell arteritis or arteritic ischemic optic neuropathy].<br>II. has NAION in 60d<br>Entry events having at least 1 condition occurrence of [Nonarteritic Anterior Ischemic Optic Neuropathy], starting between 0 days before and 60 days after cohort entry start date.<br>III. no traumatic optic nerve injury on or prior	The person also exists the cohort at the end of continuous observation.

© 2025 Cai CX et al. JAMA Ophthalmology.

		Entry events having no condition occurrences of [traumatic optic nerve injury], starting anytime on or before cohort entry start date.<br>Limit qualifying entry events to the earliest event per person.	
“Specific” NAION	People may enter the cohort when observing any of the following:<br>1. condition occurrences of [Nonarteritic Anterior Ischemic Optic Neuropathy].<br>2. condition occurrences of [presention conditons for index date correction].	I. no giant cell arteritis (2dx)<br>Entry events with at most 0 of the following criteria:<br>1. having at least 2 distinct start dates from condition occurrences of [Giant cell arteritis or arteritic ischemic optic neuropathy], allow events outside observation period.<br>II. has 2+ NAION diagnosis with one in 60d of index and another in 90d<br>Entry events having at least 1 condition occurrence of [Nonarteritic Anterior Ischemic Optic Neuropathy], starting between 0 days before and 60 days after cohort entry start date; having at least 1 condition occurrence of [Nonarteritic Anterior Ischemic Optic Neuropathy], starting between 1 days after and 90 days after [Nonarteritic Anterior Ischemic Optic Neuropathy] start date.<br>III. no traumatic optic nerve injury on or prior<br>Entry events having no condition occurrences of [traumatic optic nerve injury], starting anytime on or before cohort entry start date; allow events outside observation period.<br>Limit qualifying entry events to the earliest event per person.	The person also exits the cohort at the end of continuous observation.

*GLP-1 receptor agonists drug of interest = semaglutide, dulaglutide, or exenatide

Abbreviations: T2DM = type 2 diabetes mellitus, NAION = non-arteritic anterior ischemic optic neuropathy

© 2025 Cai CX et al. JAMA Ophthalmology.

eTable 3: Codes from standardized vocabularies used to define clinical concepts.

Clinical Concept	Concept ID	Concept Name	Source Code	Source Vocabulary	Excluded
Secondary diabetes mellitus	195771	Secondary diabetes mellitus	8801005	SNOMED	NO
Complication due to secondary diabetes mellitus	761051	Complication due to secondary diabetes mellitus	138811000119100	SNOMED	NO
Type 1 diabetes mellitus	40484649	Type 1 diabetes mellitus well controlled	444074000	SNOMED	NO
Type 1 diabetes mellitus	42689695	Eating disorder co-occurrent with diabetes mellitus type 1	1067201000000106	SNOMED	NO
Type 1 diabetes mellitus	765533	Glaucoma due to diabetes mellitus type 1	60981000119103	SNOMED	NO
Type 1 diabetes mellitus	43531006	Maturity onset diabetes of the young, type 1	609562003	SNOMED	NO
Type 1 diabetes mellitus	765650	Cranial nerve palsy due to diabetes mellitus type 1	82561000119101	SNOMED	NO
Type 1 diabetes mellitus	45770986	Retinopathy due to unstable diabetes mellitus type 1	706894000	SNOMED	NO
Type 1 diabetes mellitus	201254	Type 1 diabetes mellitus	46635009	SNOMED	NO
Type 1 diabetes mellitus	45768456	Neuropathy due to unstable diabetes mellitus type 1	706891008	SNOMED	NO

© 2025 Cai CX et al. JAMA Ophthalmology.

Type 1 diabetes mellitus	40484648	Type 1 diabetes mellitus uncontrolled	444073006	SNOMED	NO
Type 1 diabetes mellitus	4128019	Brittle type 1 diabetes mellitus	290002008	SNOMED	NO
Type 1 diabetes mellitus	435216	Disorder due to type 1 diabetes mellitus	420868002	SNOMED	NO
Type 2 diabetes mellitus	443238	Diabetic - poor control	268519009	SNOMED	NO
Type 2 diabetes mellitus	201820	Diabetes mellitus	73211009	SNOMED	NO
Type 2 diabetes mellitus	442793	Complication due to diabetes mellitus	74627003	SNOMED	NO
Type 2 diabetes mellitus	40484648	Type 1 diabetes mellitus uncontrolled	444073006	SNOMED	YES
Type 2 diabetes mellitus	201254	Type 1 diabetes mellitus	46635009	SNOMED	YES
Type 2 diabetes mellitus	435216	Disorder due to type 1 diabetes mellitus	420868002	SNOMED	YES
Type 2 diabetes mellitus	195771	Secondary diabetes mellitus	8801005	SNOMED	YES
Type 2 diabetes mellitus	761051	Complication due to secondary diabetes mellitus	138811000119100	SNOMED	YES
Type 2 diabetes mellitus	4016045	Diabetic - good control	170763003	SNOMED	YES

© 2025 Cai CX et al. JAMA Ophthalmology.

Type 2 diabetes mellitus	40484649	Type 1 diabetes mellitus well controlled	444074000	SNOMED	YES
Type 2 diabetes mellitus	43531009	Pregnancy and type 1 diabetes mellitus	609566000	SNOMED	YES
Type 2 diabetes mellitus	4024659	Gestational diabetes mellitus	11687002	SNOMED	YES
DPP4 Inhibitors	43013884	alogliptin	1368001	RxNorm	NO
DPP4 Inhibitors	40239216	linagliptin	1100699	RxNorm	NO
DPP4 Inhibitors	40166035	saxagliptin	857974	RxNorm	NO
DPP4 Inhibitors	1580747	sitagliptin	593411	RxNorm	NO
DPP4 Inhibitors	19122137	vildagliptin	596554	RxNorm	NO
SGLT2 inhibitors	43526465	canagliflozin	1373458	RxNorm	NO
SGLT2 inhibitors	44785829	dapagliflozin	1488564	RxNorm	NO
SGLT2 inhibitors	45774751	empagliflozin	1545653	RxNorm	NO
SGLT2 inhibitors	793293	ertugliflozin	1992672	RxNorm	NO
SGLT2 inhibitors	1594973	chlorpropamid e	2404	RxNorm	NO
SGLT2 inhibitors	1597756	glimepiride	25789	RxNorm	NO
SGLT2 inhibitors	1560171	glipizide	4821	RxNorm	NO
SGLT2 inhibitors	19097821	gliquidone	25793	RxNorm	NO
SGLT2 inhibitors	1559684	glyburide	4815	RxNorm	NO

© 2025 Cai CX et al. JAMA Ophthalmology.

SGLT2 inhibitors	1502809	tolazamide	10633	RxNorm	NO
SGLT2 inhibitors	1502855	tolbutamide	10635	RxNorm	NO
Other anti-diabetics	1529331	acarbose	16681	RxNorm	NO
Other anti-diabetics	1530014	acetohexamid e	173	RxNorm	NO
Other anti-diabetics	730548	bromocriptine	1760	RxNorm	NO
Other anti-diabetics	19033498	carbutamide	2068	RxNorm	NO
Other anti-diabetics	19001409	glibornuride	102846	RxNorm	NO
Other anti-diabetics	19059796	gliclazide	4816	RxNorm	NO
Other anti-diabetics	19001441	glymidine	102848	RxNorm	NO
Other anti-diabetics	1510202	miglitol	30009	RxNorm	NO
Other anti-diabetics	1502826	nateglinide	274332	RxNorm	NO
Other anti-diabetics	1525215	pioglitazone	33738	RxNorm	NO
Other anti-diabetics	1516766	repaglinide	73044	RxNorm	NO
Other anti-diabetics	1547504	rosiglitazone	84108	RxNorm	NO
Other anti-diabetics	1515249	troglitazone	72610	RxNorm	NO
Insulin	1596977	insulin, regular, human	253182	RxNorm	NO
Insulin	1550023	insulin lispro	86009	RxNorm	NO
Insulin	1567198	insulin aspart, human	51428	RxNorm	NO
Insulin	1502905	insulin glargine	274783	RxNorm	NO

© 2025 Cai CX et al. JAMA Ophthalmology.

Insulin	1513876	insulin lispro protamine, human	314684	RxNorm	NO
Insulin	1531601	insulin aspart protamine, human	352385	RxNorm	NO
Insulin	1586346	insulin, regular, pork	221109	RxNorm	NO
Insulin	1544838	insulin glulisine, human	400008	RxNorm	NO
Insulin	1516976	insulin detemir	139825	RxNorm	NO
Insulin	1590165	insulin, regular, beef-pork	235275	RxNorm	NO
Insulin	1513849	lente insulin, human	314683	RxNorm	NO
Insulin	1562586	lente insulin, pork	93108	RxNorm	NO
Insulin	1588986	insulin human, rDNA origin	631657	RxNorm	NO
Insulin	1513843	lente insulin, beef-pork	314682	RxNorm	NO
Insulin	1586369	ultralente insulin, human	221110	RxNorm	NO
Insulin	35605670	insulin glargine	1740938	RxNorm	NO
Insulin	35602717	insulin degludec	1670007	RxNorm	NO
Insulin	21600713	INSULINS AND ANALOGUES	A10A	ATC	NO
Insulin	19078608	insulin, protamine zinc, beef-pork 100 unit/ml Injectable Suspension	311053	RxNorm	NO
Metformin	1503297	metformin	6809	RxNorm	NO
semaglutide	793143	semaglutide	1991302	RxNorm	NO

© 2025 Cai CX et al. JAMA Ophthalmology.

GLP-1 receptor agonists excluding semaglutide	45774435	dulaglutide	1551291	RxNorm	NO
GLP-1 receptor agonists excluding semaglutide	1583722	exenatide	60548	RxNorm	NO
GLP-1 receptor agonists excluding semaglutide	40170911	liraglutide	475968	RxNorm	NO
GLP-1 receptor agonists excluding semaglutide	44506754	lixisenatide	1440051	RxNorm	NO
GLP-1 receptor agonists excluding semaglutide	793143	semaglutide	1991302	RxNorm	YES
GLP-1 receptor agonists excluding semaglutide	44816332	albiglutide	1534763	RxNorm	NO
Dulaglutide	45774435	dulaglutide	1551291	RxNorm	NO
GLP-1 receptor agonists excluding dulaglutide	45774435	dulaglutide	1551291	RxNorm	YES
GLP-1 receptor agonists excluding dulaglutide	1583722	exenatide	60548	RxNorm	NO

© 2025 Cai CX et al. JAMA Ophthalmology.

GLP-1 receptor agonists excluding dulaglutide	40170911	liraglutide	475968	RxNorm	NO
GLP-1 receptor agonists excluding dulaglutide	44506754	lixisenatide	1440051	RxNorm	NO
GLP-1 receptor agonists excluding dulaglutide	793143	semaglutide	1991302	RxNorm	NO
GLP-1 receptor agonists excluding dulaglutide	44816332	albiglutide	1534763	RxNorm	NO
exenatide	1583722	exenatide	60548	RxNorm	NO
GLP-1 receptor agonists excluding exenatide	45774435	dulaglutide	1551291	RxNorm	NO
GLP-1 receptor agonists excluding exenatide	1583722	exenatide	60548	RxNorm	YES
GLP-1 receptor agonists excluding exenatide	40170911	liraglutide	475968	RxNorm	NO
GLP-1 receptor agonists excluding exenatide	44506754	lixisenatide	1440051	RxNorm	NO

© 2025 Cai CX et al. JAMA Ophthalmology.

GLP-1 receptor agonists excluding exenatide	793143	semaglutide	1991302	RxNorm	NO
GLP-1 receptor agonists excluding exenatide	44816332	albiglutide	1534763	RxNorm	NO
empagliflozin	45774751	empagliflozin	1545653	RxNorm	NO
GLP-1 receptor agonists	45774435	dulaglutide	1551291	RxNorm	NO
GLP-1 receptor agonists	1583722	exenatide	60548	RxNorm	NO
GLP-1 receptor agonists	40170911	liraglutide	475968	RxNorm	NO
GLP-1 receptor agonists	44506754	lixisenatide	1440051	RxNorm	NO
GLP-1 receptor agonists	793143	semaglutide	1991302	RxNorm	NO
GLP-1 receptor agonists	44816332	albiglutide	1534763	RxNorm	NO
sitagliptin	1580747	sitagliptin	593411	RxNorm	NO
glipizide	1560171	glipizide	4821	RxNorm	NO
Non-arteritic anterior ischemic optic neuropathy	373487	Ischemic optic neuropathy	14357004	SNOMED	NO
Non-arteritic anterior ischemic optic neuropathy	4047917	Arteritic ischemic optic neuropathy	230508004	SNOMED	YES
Giant cell arteritis or arteritic	4290976	Temporal arteritis	400130008	SNOMED	NO

© 2025 Cai CX et al. JAMA Ophthalmology.

ischemic optic neuropathy					
Giant cell arteritis or arteritic ischemic optic neuropathy	37116696	Arteritic anterior ischemic optic neuropathy	733506009	SNOMED	NO
Presention conditons for index date correction	4134605	Optic disc disorder	128331005	SNOMED	NO
Presention conditons for index date correction	374954	Optic neuritis	66760008	SNOMED	NO
Presention conditons for index date correction	377286	Visual field defect	12184005	SNOMED	NO
Presention conditons for index date correction	4308632	Optic disc edema	423341008	SNOMED	NO
Traumatic optic nerve injury	375428	Traumatic optic nerve injury	48233004	SNOMED	NO

Abbreviations:
ATC = Anatomical Therapeutic Chemical Classification System
DPP4 = Dipeptidyl peptidase-4
GLP-1 = Glucagon-Like Peptide 1
SGLT2 = Sodium-Glucose Cotransporter-2
SNOMED = Systemized Nomenclature of Medicine

© 2025 Cai CX et al. JAMA Ophthalmology.

eTable 4: Non-standard OMOP to standard OMOP concept mapping.

See separate online-only document

© 2025 Cai CX et al. JAMA Ophthalmology.

eTable 5: Results from the study diagnostics for the new-user active-comparator cohort design analysis for the “sensitive” NAION definition.

Comparisons	Database	Shared maximum standardized mean difference (balance diagnostic)	Equipoise (equipoise diagnostic)	MDRR (MDRR diagnostic)	EASE (EASE diagnostic)	All Diagnostics Passed*
**Semaglutide vs. Dulaglutide**	CCAE	0.05 (pass)	0.59 (pass)	9.85 (pass)	0.05 (pass)	Yes
	CUMC	0.36 (fail)	0.55 (pass)	NA (fail)	0.42 (fail)	No
	Clinformatics	0.05 (pass)	0.61 (pass)	9.85 (pass)	0.08 (pass)	Yes
	IQVIA	0.03 (pass)	0.69 (pass)	2.11 (pass)	0.06 (pass)	Yes
	JHME	0.25 (fail)	0.58 (pass)	271.29 (pass)	0.25 (pass)	No
	MDCD	0.13 (fail)	0.6 (pass)	NA (fail)	0.11 (pass)	No
	MDCR	0.16 (fail)	0.64 (pass)	NA (fail)	0.11 (pass)	No
	OHSU	0.39 (fail)	0.61 (pass)	NA (fail)	0.55 (fail)	No
	Optum EHR	0.05 (pass)	0.56 (pass)	25.41 (pass)	0.1 (pass)	Yes
	PharMetrics	0.05 (pass)	0.66 (pass)	4.47 (pass)	0.04 (pass)	Yes
	STARR	0.41 (fail)	0.55 (pass)	271.29 (pass)	0.45 (fail)	No
	USC	0.65 (fail)	1 (pass)	NA (fail)	NA (not evaluated)	No
	VA	0.25 (fail)	0.21 (pass)	NA (fail)	0.21 (pass)	No
	WU	0.22 (fail)	0.87 (pass)	NA (fail)	0.14 (pass)	No
**Semaglutide vs. Exenatide**	CCAE	0.18 (fail)	0.12 (fail)	NA (fail)	0.14 (pass)	No
	CUMC	NA (pass)	0 (fail)	NA (fail)	NA (not evaluated)	No
	Clinformatics	0.15 (fail)	0.17 (fail)	52.56 (pass)	0.1 (pass)	No
	IQVIA	0.05 (pass)	0.17 (fail)	5.88 (pass)	0.07 (pass)	No
	JHME	NA (pass)	0 (fail)	NA (fail)	NA (not evaluated)	No
	MDCD	0.29 (fail)	0.21 (pass)	NA (fail)	0.44 (fail)	No
	MDCR	1.21 (fail)	0.13 (fail)	NA (fail)	NA (not evaluated)	No
	OHSU	0.83 (fail)	1 (pass)	NA (fail)	NA (not evaluated)	No
	Optum EHR	0.14 (fail)	0.17 (fail)	NA (fail)	0.24 (pass)	No

© 2025 Cai CX et al. JAMA Ophthalmology.

	PharMetrics	0.12 (fail)	0.17 (fail)	271.29 (pass)	0.07 (pass)	No
	STARR	1.4 (fail)	1 (pass)	NA (fail)	NA (not evaluated)	No
	USC	NA (not evaluated)	NA (not evaluated)	NA (fail)	NA (not evaluated)	No
	VA	NA (pass)	0 (fail)	NA (fail)	NA (not evaluated)	No
	WU	0.46 (fail)	0.14 (fail)	NA (fail)	NA (not evaluated)	No
**Semaglutide vs. Empagliflozin**	CCAE	0.05 (pass)	0.44 (pass)	4.73 (pass)	0.07 (pass)	Yes
	CUMC	0.22 (fail)	0.39 (pass)	NA (fail)	0.27 (fail)	No
	Clinformatics	0.05 (pass)	0.45 (pass)	5.04 (pass)	0.06 (pass)	Yes
	IQVIA	0.04 (pass)	0.47 (pass)	1.97 (pass)	0.08 (pass)	Yes
	JHME	0.22 (fail)	0.44 (pass)	271.29 (pass)	0.31 (fail)	No
	MDCD	0.14 (fail)	0.37 (pass)	NA (fail)	0.12 (pass)	No
	MDCR	0.1 (fail)	0.48 (pass)	NA (fail)	0.08 (pass)	No
	OHSU	0.26 (fail)	0.52 (pass)	NA (fail)	0.24 (pass)	No
	Optum EHR	0.04 (pass)	0.41 (pass)	271.29 (pass)	0.05 (pass)	Yes
	PharMetrics	0.05 (pass)	0.44 (pass)	5.88 (pass)	0.06 (pass)	Yes
	STARR	0.27 (fail)	0.46 (pass)	271.29 (pass)	0.52 (fail)	No
	USC	0.42 (fail)	0.54 (pass)	NA (fail)	NA (not evaluated)	No
	VA	0.07 (pass)	0.25 (pass)	52.56 (pass)	0.07 (pass)	Yes
	WU	0.18 (fail)	0.45 (pass)	NA (fail)	0.11 (pass)	No
**Semaglutide vs. Sitagliptin**	CCAE	0.06 (pass)	0.24 (pass)	9.85 (pass)	0.11 (pass)	Yes
	CUMC	0.28 (fail)	0.32 (pass)	NA (fail)	0.36 (fail)	No
	Clinformatics	0.07 (pass)	0.22 (pass)	6.47 (pass)	0.12 (pass)	Yes
	IQVIA	0.05 (pass)	0.25 (pass)	2.35 (pass)	0.12 (pass)	Yes
	JHME	0.24 (fail)	0.3 (pass)	271.29 (pass)	0.44 (fail)	No
	MDCD	0.23 (fail)	0.3 (pass)	NA (fail)	0.14 (pass)	No
	MDCR	0.15 (fail)	0.29 (pass)	NA (fail)	0.1 (pass)	No
	OHSU	0.48 (fail)	0.42 (pass)	NA (fail)	NA (not evaluated)	No
	Optum EHR	0.05 (pass)	0.23 (pass)	NA (fail)	0.07 (pass)	No
	PharMetrics	0.07 (pass)	0.27 (pass)	5.88 (pass)	0.05 (pass)	Yes

© 2025 Cai CX et al. JAMA Ophthalmology.

	STARR	0.31 (fail)	0.28 (pass)	271.29 (pass)	0.36 (fail)	No
	USC	NA (pass)	0 (fail)	NA (fail)	NA (not evaluated)	No
	VA	0.87 (fail)	0.11 (fail)	NA (fail)	NA (not evaluated)	No
	WU	0.33 (fail)	0.3 (pass)	NA (fail)	0.16 (pass)	No
**Semaglutide vs. Glipizide**	CCAE	0.04 (pass)	0.27 (pass)	9.85 (pass)	0.06 (pass)	Yes
	CUMC	0.46 (fail)	0.26 (pass)	NA (fail)	0.44 (fail)	No
	Clinformatics	0.05 (pass)	0.27 (pass)	4.25 (pass)	0.07 (pass)	Yes
	IQVIA	0.05 (pass)	0.28 (pass)	2.13 (pass)	0.12 (pass)	Yes
	JHME	0.23 (fail)	0.27 (pass)	NA (fail)	0.33 (fail)	No
	MDCD	0.26 (fail)	0.3 (pass)	NA (fail)	0.14 (pass)	No
	MDCR	0.16 (fail)	0.31 (pass)	NA (fail)	0.11 (pass)	No
	OHSU	0.36 (fail)	0.39 (pass)	NA (fail)	0.33 (fail)	No
	Optum EHR	0.06 (pass)	0.22 (pass)	271.29 (pass)	0.09 (pass)	Yes
	PharMetrics	0.06 (pass)	0.29 (pass)	7.25 (pass)	0.12 (pass)	Yes
	STARR	0.3 (fail)	0.24 (pass)	271.29 (pass)	0.26 (fail)	No
	USC	0.73 (fail)	0.22 (pass)	NA (fail)	NA (not evaluated)	No
	VA	0.09 (pass)	0.11 (fail)	16.47 (pass)	0.12 (pass)	No
	WU	0.23 (fail)	0.3 (pass)	271.29 (pass)	0.18 (pass)	No
**Dulaglutide vs. Empagliflozin**	CCAE	0.05 (pass)	0.78 (pass)	16.47 (pass)	0.05 (pass)	Yes
	CUMC	0.35 (fail)	0.56 (pass)	NA (fail)	0.21 (pass)	No
	Clinformatics	0.06 (pass)	0.68 (pass)	9.85 (pass)	0.07 (pass)	Yes
	IQVIA	0.03 (pass)	0.68 (pass)	2.14 (pass)	0.03 (pass)	Yes
	JHME	0.23 (fail)	0.77 (pass)	NA (fail)	0.17 (pass)	No
	MDCD	0.1 (fail)	0.57 (pass)	NA (fail)	0.07 (pass)	No
	MDCR	0.13 (fail)	0.72 (pass)	NA (fail)	0.07 (pass)	No
	OHSU	0.37 (fail)	0.74 (pass)	NA (fail)	0.28 (fail)	No
	Optum EHR	0.05 (pass)	0.65 (pass)	16.47 (pass)	0.05 (pass)	Yes
	PharMetrics	0.04 (pass)	0.76 (pass)	4.47 (pass)	0.04 (pass)	Yes
	STARR	0.38 (fail)	0.71 (pass)	NA (fail)	0.36 (fail)	No
	USC	0.56 (fail)	1 (pass)	NA (fail)	NA (not evaluated)	No

© 2025 Cai CX et al. JAMA Ophthalmology.

	VA	0.21 (fail)	0.17 (fail)	52.56 (pass)	0.2 (pass)	No
	WU	0.19 (fail)	0.63 (pass)	NA (fail)	0.14 (pass)	No
**Dulaglutide vs. Sitagliptin**	CCAE	0.06 (pass)	0.6 (pass)	16.47 (pass)	0.04 (pass)	Yes
	CUMC	0.34 (fail)	0.7 (pass)	NA (fail)	0.25 (pass)	No
	Clinformatics	0.07 (pass)	0.39 (pass)	12.25 (pass)	0.05 (pass)	Yes
	IQVIA	0.03 (pass)	0.49 (pass)	2.21 (pass)	0.05 (pass)	Yes
	JHME	0.24 (fail)	0.69 (pass)	NA (fail)	0.22 (pass)	No
	MDCD	0.11 (fail)	0.37 (pass)	NA (fail)	0.08 (pass)	No
	MDCR	0.2 (fail)	0.6 (pass)	NA (fail)	0.15 (pass)	No
	Optum EHR	NA (pass)	0 (fail)	NA (fail)	NA (not evaluated)	No
	PharMetrics	0.05 (pass)	0.55 (pass)	3.89 (pass)	0.03 (pass)	Yes
	STARR	0.34 (fail)	0.55 (pass)	NA (fail)	0.33 (fail)	No
	USC	0.86 (fail)	0.45 (pass)	NA (fail)	NA (not evaluated)	No
	VA	0.35 (fail)	0.6 (pass)	NA (fail)	0.73 (fail)	No
	WU	0.28 (fail)	0.54 (pass)	NA (fail)	0.18 (pass)	No
**Dulaglutide vs. Glipizide**	CCAE	0.05 (pass)	0.6 (pass)	25.41 (pass)	0.04 (pass)	Yes
	CUMC	0.36 (fail)	0.66 (pass)	NA (fail)	0.43 (fail)	No
	Clinformatics	0.06 (pass)	0.54 (pass)	5.88 (pass)	0.09 (pass)	Yes
	IQVIA	0.03 (pass)	0.52 (pass)	2.03 (pass)	0.05 (pass)	Yes
	JHME	0.26 (fail)	0.65 (pass)	NA (fail)	0.17 (pass)	No
	MDCD	0.1 (pass)	0.36 (pass)	271.29 (pass)	0.07 (pass)	Yes
	MDCR	0.15 (fail)	0.63 (pass)	271.29 (pass)	0.15 (pass)	No
	Optum EHR	0.05 (pass)	0.5 (pass)	9.85 (pass)	0.11 (pass)	Yes
	PharMetrics	0.04 (pass)	0.58 (pass)	4.73 (pass)	0.03 (pass)	Yes
	STARR	0.43 (fail)	0.52 (pass)	NA (fail)	0.27 (fail)	No
	USC	0.56 (fail)	0.36 (pass)	NA (fail)	NA (not evaluated)	No
	VA	0.2 (fail)	0.18 (fail)	271.29 (pass)	0.21 (pass)	No
	WU	0.24 (fail)	0.52 (pass)	271.29 (pass)	0.19 (pass)	No
**Exenatide vs. Empagliflozin**	CCAE	0.15 (fail)	0.38 (pass)	271.29 (pass)	0.09 (pass)	No

© 2025 Cai CX et al. JAMA Ophthalmology.

	CUMC	1.13 (fail)	1 (pass)	NA (fail)	NA (not evaluated)	No
	Clinformatics	0.13 (fail)	0.34 (pass)	52.56 (pass)	0.08 (pass)	No
	IQVIA	0.11 (fail)	0.32 (pass)	16.47 (pass)	0.05 (pass)	No
	JHME	0.97 (fail)	0.53 (pass)	NA (fail)	NA (not evaluated)	No
	MDCD	0.23 (fail)	0.32 (pass)	NA (fail)	0.12 (pass)	No
	MDCR	0.53 (fail)	0.43 (pass)	NA (fail)	0.63 (fail)	No
	Optum EHR	0.13 (fail)	0.38 (pass)	NA (fail)	0.25 (fail)	No
	PharMetrics	0.11 (fail)	0.38 (pass)	52.56 (pass)	0.1 (pass)	No
	STARR	1.13 (fail)	1 (pass)	NA (fail)	NA (not evaluated)	No
	USC	NA (not evaluated)	NA (not evaluated)	NA (fail)	NA (not evaluated)	No
	VA	0.79 (fail)	0.29 (pass)	NA (fail)	NA (not evaluated)	No
	WU	0.5 (fail)	0.47 (pass)	NA (fail)	0.57 (fail)	No
**Exenatide vs. Sitagliptin**	CCAE	0.16 (fail)	0.69 (pass)	271.29 (pass)	0.14 (pass)	No
	CUMC	1.13 (fail)	1 (pass)	NA (fail)	NA (not evaluated)	No
	Clinformatics	0.13 (fail)	0.4 (pass)	52.56 (pass)	0.08 (pass)	No
	IQVIA	0.06 (pass)	0.61 (pass)	9.85 (pass)	0.04 (pass)	Yes
	JHME	0.87 (fail)	1 (pass)	NA (fail)	NA (not evaluated)	No
	MDCD	0.17 (fail)	0.5 (pass)	NA (fail)	0.15 (pass)	No
	MDCR	0.63 (fail)	0.85 (pass)	NA (fail)	0.39 (fail)	No
	Optum EHR	0.12 (fail)	0.61 (pass)	NA (fail)	0.47 (fail)	No
	PharMetrics	0.11 (fail)	0.65 (pass)	52.56 (pass)	0.1 (pass)	No
	STARR	1.32 (fail)	1 (pass)	NA (fail)	NA (not evaluated)	No
	USC	NA (not evaluated)	NA (not evaluated)	NA (fail)	NA (not evaluated)	No
	VA	0.72 (fail)	1 (pass)	NA (fail)	NA (not evaluated)	No
	WU	0.49 (fail)	1 (pass)	NA (fail)	0.52 (fail)	No
**Exenatide vs. Glipizide**	CCAE	0.13 (fail)	0.52 (pass)	271.29 (pass)	0.16 (pass)	No

© 2025 Cai CX et al. JAMA Ophthalmology.

	CUMC	0.96 (fail)	1 (pass)	NA (fail)	NA (not evaluated)	No
	Clinformatics	0.14 (fail)	0.5 (pass)	25.41 (pass)	0.08 (pass)	No
	IQVIA	0.11 (fail)	0.52 (pass)	6.47 (pass)	0.03 (pass)	No
	JHME	1.21 (fail)	1 (pass)	NA (fail)	NA (not evaluated)	No
	MDCD	0.17 (fail)	0.47 (pass)	NA (fail)	0.19 (pass)	No
	MDCR	0.57 (fail)	0.74 (pass)	NA (fail)	0.39 (fail)	No
	Optum EHR	0.12 (fail)	0.59 (pass)	NA (fail)	0.17 (pass)	No
	PharMetrics	0.12 (fail)	0.53 (pass)	52.56 (pass)	0.13 (pass)	No
	STARR	1.32 (fail)	1 (pass)	NA (fail)	NA (not evaluated)	No
	USC	NA (not evaluated)	NA (not evaluated)	NA (fail)	NA (not evaluated)	No
	VA	0.73 (fail)	0.34 (pass)	NA (fail)	NA (not evaluated)	No
	WU	0.45 (fail)	0.92 (pass)	NA (fail)	1.02 (fail)	No

* Only databases that passed all study diagnostics contributed to the specific drug target-comparator analysis.

Abbreviations: NAION = non-arteritic anterior ischemic optic neuropathy, MDRR = minimum detectable relative risk, EASE = Expected Absolute Systematic Error

Database abbreviations:
CCAE = Merative MarketScan Commercial Claims and Encounters Database
Clinformatics = Optum's de-identified Clinformatics Data Mart Database
CUMC = Columbia University Medical Center
IQVIA = IQVIA Open Claims
JHME = Johns Hopkins Medical Enterprise
MDCD = Merative MarketScan Multi-State Medicaid Database
MDCR = Merative MarketScan Medicare Supplemental and Coordination of Benefits Database
OHSU = Oregon Health & Science University
Optum EHR = Optum de-identified Electronic Health Record data set
PharMetrics = PharMetrics Plus
STARR = Stanford University
USC = Keck Medical Center of University of Southern California
VA = Department of Veterans Affairs
WashU = Washington University in St. Louis

© 2025 Cai CX et al. JAMA Ophthalmology.

eTable 6: Results from the study diagnostics for the new-user active-comparator cohort design analysis for the “specific” NAION definition.

Comparisons	Database	Maximum standardized mean difference (balance diagnostic)	Equipoise (equipoise diagnostic)	MDRR (MDRR diagnostic)	EASE (EASE diagnostic)	All Diagnostics Passed*
**Semaglutide vs. Dulaglutide**	CCAE	0.05 (pass)	0.59 (pass)	16.47 (pass)	0.05 (pass)	Yes
	CUMC	0.36 (fail)	0.55 (pass)	NA (fail)	0.42 (fail)	No
	Clinformatics	0.05 (pass)	0.61 (pass)	16.47 (pass)	0.08 (pass)	Yes
	IQVIA	0.03 (pass)	0.69 (pass)	2.94 (pass)	0.06 (pass)	Yes
	JHME	0.25 (fail)	0.58 (pass)	NA (fail)	0.25 (pass)	No
	MDCD	0.13 (fail)	0.6 (pass)	NA (fail)	0.11 (pass)	No
	MDCR	0.16 (fail)	0.64 (pass)	NA (fail)	0.11 (pass)	No
	OHSU	0.39 (fail)	0.61 (pass)	NA (fail)	0.55 (fail)	No
	Optum EHR	0.05 (pass)	0.56 (pass)	52.56 (pass)	0.1 (pass)	Yes
	PharMetrics	0.05 (pass)	0.66 (pass)	6.47 (pass)	0.04 (pass)	Yes
	STARR	0.41 (fail)	0.55 (pass)	271.29 (pass)	0.45 (fail)	No
	USC	0.65 (fail)	1 (pass)	NA (fail)	NA (not evaluated)	No
	VA	0.25 (fail)	0.21 (pass)	NA (fail)	0.21 (pass)	No
	WU	0.22 (fail)	0.87 (pass)	NA (fail)	0.14 (pass)	No
**Semaglutide vs. Exenatide**	CCAE	0.18 (fail)	0.12 (fail)	NA (fail)	0.14 (pass)	No
	CUMC	NA (pass)	0 (fail)	NA (fail)	NA (not evaluated)	No
	Clinformatics	0.15 (fail)	0.17 (fail)	271.29 (pass)	0.1 (pass)	No
	IQVIA	0.05 (pass)	0.17 (fail)	12.25 (pass)	0.07 (pass)	No
	JHME	NA (pass)	0 (fail)	NA (fail)	NA (not evaluated)	No
	MDCD	0.29 (fail)	0.21 (pass)	NA (fail)	0.44 (fail)	No
	MDCR	1.21 (fail)	0.13 (fail)	NA (fail)	NA (not evaluated)	No
	OHSU	0.83 (fail)	1 (pass)	NA (fail)	NA (not evaluated)	No
	Optum EHR	0.14 (fail)	0.17 (fail)	NA (fail)	0.24 (pass)	No

© 2025 Cai CX et al. JAMA Ophthalmology.

	PharMetrics	0.12 (fail)	0.17 (fail)	271.29 (pass)	0.07 (pass)	No
	STARR	1.4 (fail)	1 (pass)	NA (fail)	NA (not evaluated)	No
	USC	NA (not evaluated)	NA (not evaluated)	NA (fail)	NA (not evaluated)	No
	VA	NA (pass)	0 (fail)	NA (fail)	NA (not evaluated)	No
	WU	0.46 (fail)	0.14 (fail)	NA (fail)	NA (not evaluated)	No
**Semaglutide vs. Empagliflozin**	CCAE	0.05 (pass)	0.44 (pass)	7.25 (pass)	0.07 (pass)	Yes
	CUMC	0.22 (fail)	0.39 (pass)	NA (fail)	0.27 (fail)	No
	Clinformatics	0.05 (pass)	0.45 (pass)	7.25 (pass)	0.06 (pass)	Yes
	IQVIA	0.04 (pass)	0.47 (pass)	2.61 (pass)	0.08 (pass)	Yes
	JHME	0.22 (fail)	0.44 (pass)	NA (fail)	0.31 (fail)	No
	MDCD	0.14 (fail)	0.37 (pass)	NA (fail)	0.12 (pass)	No
	MDCR	0.1 (fail)	0.48 (pass)	271.29 (pass)	0.08 (pass)	No
	OHSU	0.26 (fail)	0.52 (pass)	NA (fail)	0.24 (pass)	No
	Optum EHR	0.04 (pass)	0.41 (pass)	271.29 (pass)	0.05 (pass)	Yes
	PharMetrics	0.05 (pass)	0.44 (pass)	12.25 (pass)	0.06 (pass)	Yes
	STARR	0.27 (fail)	0.46 (pass)	271.29 (pass)	0.52 (fail)	No
	USC	0.42 (fail)	0.54 (pass)	NA (fail)	NA (not evaluated)	No
	VA	0.07 (pass)	0.25 (pass)	52.56 (pass)	0.07 (pass)	Yes
	WU	0.18 (fail)	0.45 (pass)	NA (fail)	0.11 (pass)	No
**Semaglutide vs. Sitagliptin**	CCAE	0.06 (pass)	0.24 (pass)	25.41 (pass)	0.11 (pass)	Yes
	CUMC	0.28 (fail)	0.32 (pass)	NA (fail)	0.36 (fail)	No
	Clinformatics	0.07 (pass)	0.22 (pass)	8.31 (pass)	0.12 (pass)	Yes
	IQVIA	0.05 (pass)	0.25 (pass)	3.5 (pass)	0.12 (pass)	Yes
	JHME	0.24 (fail)	0.3 (pass)	NA (fail)	0.44 (fail)	No
	MDCD	0.23 (fail)	0.3 (pass)	NA (fail)	0.14 (pass)	No
	MDCR	0.15 (fail)	0.29 (pass)	NA (fail)	0.1 (pass)	No
	OHSU	0.48 (fail)	0.42 (pass)	NA (fail)	NA (not evaluated)	No

© 2025 Cai CX et al. JAMA Ophthalmology.

	Optum EHR	0.05 (pass)	0.23 (pass)	NA (fail)	0.07 (pass)	No
	PharMetrics	0.07 (pass)	0.27 (pass)	12.25 (pass)	0.05 (pass)	Yes
	STARR	0.31 (fail)	0.28 (pass)	271.29 (pass)	0.36 (fail)	No
	USC	NA (pass)	0 (fail)	NA (fail)	NA (not evaluated)	No
	VA	0.87 (fail)	0.11 (fail)	NA (fail)	NA (not evaluated)	No
	WU	0.33 (fail)	0.3 (pass)	NA (fail)	0.16 (pass)	No
**Semaglutide vs. Glipizide**	CCAE	0.04 (pass)	0.27 (pass)	25.41 (pass)	0.06 (pass)	Yes
	CUMC	0.46 (fail)	0.26 (pass)	NA (fail)	0.44 (fail)	No
	Clinformatics	0.05 (pass)	0.27 (pass)	4.73 (pass)	0.07 (pass)	Yes
	IQVIA	0.05 (pass)	0.28 (pass)	3.07 (pass)	0.12 (pass)	Yes
	JHME	0.23 (fail)	0.27 (pass)	NA (fail)	0.33 (fail)	No
	MDCD	0.26 (fail)	0.3 (pass)	NA (fail)	0.14 (pass)	No
	MDCR	0.16 (fail)	0.31 (pass)	NA (fail)	0.11 (pass)	No
	OHSU	0.36 (fail)	0.39 (pass)	NA (fail)	0.33 (fail)	No
	Optum EHR	0.06 (pass)	0.22 (pass)	NA (fail)	0.09 (pass)	No
	PharMetrics	0.06 (pass)	0.29 (pass)	12.25 (pass)	0.12 (pass)	Yes
	STARR	0.3 (fail)	0.24 (pass)	271.29 (pass)	0.26 (fail)	No
	USC	0.73 (fail)	0.22 (pass)	NA (fail)	NA (not evaluated)	No
	VA	0.09 (pass)	0.11 (fail)	52.56 (pass)	0.12 (pass)	No
	WU	0.23 (fail)	0.3 (pass)	NA (fail)	0.18 (pass)	No
**Dulaglutide vs. Empagliflozin**	CCAE	0.05 (pass)	0.78 (pass)	25.41 (pass)	0.05 (pass)	Yes
	CUMC	0.35 (fail)	0.56 (pass)	NA (fail)	0.21 (pass)	No
	Clinformatics	0.06 (pass)	0.68 (pass)	271.29 (pass)	0.07 (pass)	Yes
	IQVIA	0.03 (pass)	0.68 (pass)	3.07 (pass)	0.03 (pass)	Yes
	JHME	0.23 (fail)	0.77 (pass)	NA (fail)	0.17 (pass)	No
	MDCD	0.1 (fail)	0.57 (pass)	NA (fail)	0.07 (pass)	No
	MDCR	0.13 (fail)	0.72 (pass)	NA (fail)	0.07 (pass)	No
	OHSU	0.37 (fail)	0.74 (pass)	NA (fail)	0.28 (fail)	No
	Optum EHR	0.05 (pass)	0.65 (pass)	16.47 (pass)	0.05 (pass)	Yes

© 2025 Cai CX et al. JAMA Ophthalmology.

	PharMetrics	0.04 (pass)	0.76 (pass)	8.31 (pass)	0.04 (pass)	Yes
	STARR	0.38 (fail)	0.71 (pass)	NA (fail)	0.36 (fail)	No
	USC	0.56 (fail)	1 (pass)	NA (fail)	NA (not evaluated)	No
	VA	0.21 (fail)	0.17 (fail)	52.56 (pass)	0.2 (pass)	No
	WU	0.19 (fail)	0.63 (pass)	NA (fail)	0.14 (pass)	No
**Dulaglutide vs. Sitagliptin**	CCAE	0.06 (pass)	0.6 (pass)	25.41 (pass)	0.04 (pass)	Yes
	CUMC	0.34 (fail)	0.7 (pass)	NA (fail)	0.25 (pass)	No
	Clinformatics	0.07 (pass)	0.39 (pass)	16.47 (pass)	0.05 (pass)	Yes
	IQVIA	0.03 (pass)	0.49 (pass)	3.22 (pass)	0.05 (pass)	Yes
	JHME	0.24 (fail)	0.69 (pass)	NA (fail)	0.22 (pass)	No
	MDCD	0.11 (fail)	0.37 (pass)	NA (fail)	0.08 (pass)	No
	MDCR	0.2 (fail)	0.6 (pass)	NA (fail)	0.15 (pass)	No
	Optum EHR	NA (pass)	0 (fail)	NA (fail)	NA (not evaluated)	No
	PharMetrics	0.05 (pass)	0.55 (pass)	6.47 (pass)	0.03 (pass)	Yes
	STARR	0.34 (fail)	0.55 (pass)	NA (fail)	0.33 (fail)	No
	USC	0.86 (fail)	0.45 (pass)	NA (fail)	NA (not evaluated)	No
	VA	0.35 (fail)	0.6 (pass)	NA (fail)	0.73 (fail)	No
	WU	0.28 (fail)	0.54 (pass)	NA (fail)	0.18 (pass)	No
**Dulaglutide vs. Glipizide**	CCAE	0.05 (pass)	0.6 (pass)	52.56 (pass)	0.04 (pass)	Yes
	CUMC	0.36 (fail)	0.66 (pass)	NA (fail)	0.43 (fail)	No
	Clinformatics	0.06 (pass)	0.54 (pass)	7.25 (pass)	0.09 (pass)	Yes
	IQVIA	0.03 (pass)	0.52 (pass)	3 (pass)	0.05 (pass)	Yes
	JHME	0.26 (fail)	0.65 (pass)	NA (fail)	0.17 (pass)	No
	MDCD	0.1 (pass)	0.36 (pass)	NA (fail)	0.07 (pass)	No
	MDCR	0.15 (fail)	0.63 (pass)	NA (fail)	0.15 (pass)	No
	Optum EHR	0.05 (pass)	0.5 (pass)	12.25 (pass)	0.11 (pass)	Yes
	PharMetrics	0.04 (pass)	0.58 (pass)	6.47 (pass)	0.03 (pass)	Yes
	STARR	0.43 (fail)	0.52 (pass)	NA (fail)	0.27 (fail)	No
	USC	0.56 (fail)	0.36 (pass)	NA (fail)	NA (not evaluated)	No
	VA	0.2 (fail)	0.18 (fail)	271.29 (pass)	0.21 (pass)	No
	WU	0.24 (fail)	0.52 (pass)	NA (fail)	0.19 (pass)	No

© 2025 Cai CX et al. JAMA Ophthalmology.

**Exenatide vs. Empagliflozin**	CCAE	0.15 (fail)	0.38 (pass)	NA (fail)	0.09 (pass)	No
	CUMC	1.13 (fail)	1 (pass)	NA (fail)	NA (not evaluated)	No
	Clinformatics	0.13 (fail)	0.34 (pass)	52.56 (pass)	0.08 (pass)	No
	IQVIA	0.11 (fail)	0.32 (pass)	52.56 (pass)	0.05 (pass)	No
	JHME	0.97 (fail)	0.53 (pass)	NA (fail)	NA (not evaluated)	No
	MDCD	0.23 (fail)	0.32 (pass)	NA (fail)	0.12 (pass)	No
	MDCR	0.53 (fail)	0.43 (pass)	NA (fail)	0.63 (fail)	No
	Optum EHR	0.13 (fail)	0.38 (pass)	NA (fail)	0.25 (fail)	No
	PharMetrics	0.11 (fail)	0.38 (pass)	52.56 (pass)	0.1 (pass)	No
	STARR	1.13 (fail)	1 (pass)	NA (fail)	NA (not evaluated)	No
	USC	NA (not evaluated)	NA (not evaluated)	NA (fail)	NA (not evaluated)	No
	VA	0.79 (fail)	0.29 (pass)	NA (fail)	NA (not evaluated)	No
	WU	0.5 (fail)	0.47 (pass)	NA (fail)	0.57 (fail)	No
**Exenatide vs. Sitagliptin**	CCAE	0.16 (fail)	0.69 (pass)	NA (fail)	0.14 (pass)	No
	CUMC	1.13 (fail)	1 (pass)	NA (fail)	NA (not evaluated)	No
	Clinformatics	0.13 (fail)	0.4 (pass)	52.56 (pass)	0.08 (pass)	No
	IQVIA	0.06 (pass)	0.61 (pass)	12.25 (pass)	0.04 (pass)	Yes
	JHME	0.87 (fail)	1 (pass)	NA (fail)	NA (not evaluated)	No
	MDCD	0.17 (fail)	0.5 (pass)	NA (fail)	0.15 (pass)	No
	MDCR	0.63 (fail)	0.85 (pass)	NA (fail)	0.39 (fail)	No
	Optum EHR	0.12 (fail)	0.61 (pass)	NA (fail)	0.47 (fail)	No
	PharMetrics	0.11 (fail)	0.65 (pass)	271.29 (pass)	0.1 (pass)	No
	STARR	1.32 (fail)	1 (pass)	NA (fail)	NA (not evaluated)	No
	USC	NA (not evaluated)	NA (not evaluated)	NA (fail)	NA (not evaluated)	No
	VA	0.72 (fail)	1 (pass)	NA (fail)	NA (not evaluated)	No
	WU	0.49 (fail)	1 (pass)	NA (fail)	0.52 (fail)	No

© 2025 Cai CX et al. JAMA Ophthalmology.

**Exenatide vs. Glipizide**	CCAE	0.13 (fail)	0.52 (pass)	NA (fail)	0.16 (pass)	No
	CUMC	0.96 (fail)	1 (pass)	NA (fail)	NA (not evaluated)	No
	Clinformatics	0.14 (fail)	0.5 (pass)	16.47 (pass)	0.08 (pass)	No
	IQVIA	0.11 (fail)	0.52 (pass)	25.41 (pass)	0.03 (pass)	No
	JHME	1.21 (fail)	1 (pass)	NA (fail)	NA (not evaluated)	No
	MDCD	0.17 (fail)	0.47 (pass)	NA (fail)	0.19 (pass)	No
	MDCR	0.57 (fail)	0.74 (pass)	NA (fail)	0.39 (fail)	No
	Optum EHR	0.12 (fail)	0.59 (pass)	NA (fail)	0.17 (pass)	No
	PharMetrics	0.12 (fail)	0.53 (pass)	52.56 (pass)	0.13 (pass)	No
	STARR	1.32 (fail)	1 (pass)	NA (fail)	NA (not evaluated)	No
	USC	NA (not evaluated)	NA (not evaluated)	NA (fail)	NA (not evaluated)	No
	VA	0.73 (fail)	0.34 (pass)	NA (fail)	NA (not evaluated)	No
	WU	0.45 (fail)	0.92 (pass)	NA (fail)	1.02 (fail)	No

* Only databases that passed all study diagnostics contributed to the specific drug target-comparator analysis.

Abbreviations: NAION = non-arteritic anterior ischemic optic neuropathy, MDRR = minimum detectable relative risk, EASE = Expected Absolute Systematic Error

Database abbreviations:
CCAE = Merative MarketScan Commercial Claims and Encounters Database
Clinformatics = Optum's de-identified Clinformatics Data Mart Database
CUMC = Columbia University Medical Center
IQVIA = IQVIA Open Claims
JHME = Johns Hopkins Medical Enterprise
MDCD = Merative MarketScan Multi-State Medicaid Database
MDCR = Merative MarketScan Medicare Supplemental and Coordination of Benefits Database
OHSU = Oregon Health & Science University
Optum EHR = Optum de-identified Electronic Health Record data set
PharMetrics = PharMetrics Plus
STARR = Stanford University
USC = Keck Medical Center of University of Southern California
VA = Department of Veterans Affairs
WashU = Washington University in St. Louis

© 2025 Cai CX et al. JAMA Ophthalmology.

eTable 7: Results from the study diagnostics for the self-controlled case-series analysis for the “sensitive” NAION definition.

Exposure	Database	MDRR (MDRR diagnostic)	EASE (EASE diagnostic)	Time trend (time trend diagnostic)	Pre-exposure (pre-exposure diagnostic)	All Diagnostics Passed*
**Semaglutide**	CCAE	1.53 (pass)	0.05 (pass)	1 (pass)	0.36 (pass)	Yes
	CUMC	13.2 (pass)	0.29 (fail)	1 (pass)	NA (not evaluated)	No
	Clinformatics	1.38 (pass)	0.08 (pass)	1 (pass)	0.97 (pass)	Yes
	IQVIA	1.17 (pass)	0.12 (pass)	1 (pass)	0.54 (pass)	Yes
	JHME	4.02 (pass)	0.06 (pass)	1 (pass)	NA (not evaluated)	Yes
	MDCD	3.56 (pass)	0.1 (pass)	1 (pass)	NA (not evaluated)	Yes
	MDCR	2.52 (pass)	0.02 (pass)	1 (pass)	0.8 (pass)	Yes
	OHSU	13.52 (pass)	0.29 (fail)	1 (pass)	NA (not evaluated)	No
	Optum EHR	1.88 (pass)	0.35 (fail)	1 (pass)	0.91 (pass)	No
	PharMetrics	1.33 (pass)	0.06 (pass)	1 (pass)	0.1 (pass)	Yes
	STARR	10.6 (pass)	0.24 (pass)	0.34 (pass)	NA (not evaluated)	Yes
	USC	77.35 (pass)	0.05 (pass)	0.01 (fail)	NA (not evaluated)	No
	VA	1.35 (pass)	0.1 (pass)	1 (pass)	0.32 (pass)	Yes
	WU	5.28 (pass)	0.05 (pass)	1 (pass)	0.95 (pass)	Yes
**Dulaglutide**	CCAE	1.6 (pass)	0.05 (pass)	1 (pass)	0.14 (pass)	Yes
	CUMC	12.97 (pass)	0.29 (fail)	1 (pass)	NA (not evaluated)	No
	Clinformatics	1.35 (pass)	0.08 (pass)	1 (pass)	NA (not evaluated)	Yes
	IQVIA	1.16 (pass)	0.12 (pass)	1 (pass)	0.03 (fail)	No
	JHME	5.04 (pass)	0.04 (pass)	1 (pass)	NA (not evaluated)	Yes
	MDCD	1.9 (pass)	0.1 (pass)	1 (pass)	0.94 (pass)	Yes
	MDCR	2.36 (pass)	0.02 (pass)	1 (pass)	NA (not evaluated)	Yes
	OHSU	11.07 (pass)	0.29 (fail)	1 (pass)	NA (not evaluated)	No
	Optum EHR	1.82 (pass)	0.35 (fail)	1 (pass)	0.94 (pass)	No
	PharMetrics	1.36 (pass)	0.06 (pass)	1 (pass)	0.81 (pass)	Yes
	STARR	7.89 (pass)	0.24 (pass)	0.34 (pass)	NA (not evaluated)	Yes

© 2025 Cai CX et al. JAMA Ophthalmology.

	USC	22026.47 (pass)	0.05 (pass)	0.01 (fail)	0.88 (pass)	No
	VA	1.79 (pass)	0.1 (pass)	1 (pass)	0.11 (pass)	Yes
	WU	6.24 (pass)	0.05 (pass)	1 (pass)	0.88 (pass)	Yes
**Exenatide**	CCAE	3.2 (pass)	0.05 (pass)	1 (pass)	NA (not evaluated)	Yes
	CUMC	854.16 (pass)	0.29 (fail)	1 (pass)	NA (not evaluated)	No
	Clinformatics	2.18 (pass)	0.08 (pass)	1 (pass)	0.71 (pass)	Yes
	IQVIA	1.48 (pass)	0.12 (pass)	1 (pass)	0.11 (pass)	Yes
	JHME	22026.47 (pass)	0.13 (pass)	1 (pass)	NA (not evaluated)	Yes
	MDCD	3.9 (pass)	0.1 (pass)	1 (pass)	NA (not evaluated)	Yes
	MDCR	7.98 (pass)	0.02 (pass)	1 (pass)	NA (not evaluated)	Yes
	OHSU	533.91 (pass)	0.29 (fail)	1 (pass)	NA (not evaluated)	No
	Optum EHR	3.41 (pass)	0.35 (fail)	1 (pass)	0.72 (pass)	No
	PharMetrics	2.21 (pass)	0.06 (pass)	1 (pass)	NA (not evaluated)	Yes
	STARR	70.83 (pass)	0.24 (pass)	0.34 (pass)	NA (not evaluated)	Yes
	USC	NA (pass)	0.05 (pass)	0.01 (fail)	NA (not evaluated)	No
	VA	6.19 (pass)	0.1 (pass)	1 (pass)	0.88 (pass)	Yes
	WU	583.23 (pass)	0.05 (pass)	1 (pass)	NA (not evaluated)	Yes
**Empagliflozin**	CCAE	1.53 (pass)	0.05 (pass)	1 (pass)	0.21 (pass)	Yes
	CUMC	7.01 (pass)	0.29 (fail)	1 (pass)	NA (not evaluated)	No
	Clinformatics	1.3 (pass)	0.08 (pass)	1 (pass)	0.26 (pass)	Yes
	IQVIA	1.14 (pass)	0.12 (pass)	1 (pass)	0.07 (pass)	Yes
	JHME	3.97 (pass)	0.06 (pass)	1 (pass)	NA (not evaluated)	Yes
	MDCD	2.19 (pass)	0.1 (pass)	1 (pass)	0.98 (pass)	Yes
	MDCR	2.13 (pass)	0.02 (pass)	1 (pass)	0.24 (pass)	Yes
	OHSU	11.07 (pass)	0.29 (fail)	1 (pass)	NA (not evaluated)	No
	Optum EHR	1.69 (pass)	0.35 (fail)	1 (pass)	0.72 (pass)	No
	PharMetrics	1.3 (pass)	0.06 (pass)	1 (pass)	0.59 (pass)	Yes

© 2025 Cai CX et al. JAMA Ophthalmology.

	STARR	7.12 (pass)	0.24 (pass)	0.34 (pass)	NA (not evaluated)	Yes
	USC	22026.47 (pass)	0.05 (pass)	0.01 (fail)	NA (not evaluated)	No
	VA	1.25 (pass)	0.1 (pass)	1 (pass)	0.07 (pass)	Yes
	WU	4.15 (pass)	0.05 (pass)	1 (pass)	NA (not evaluated)	Yes
**Sitagliptin**	CCAE	1.55 (pass)	0.05 (pass)	1 (pass)	0.3 (pass)	Yes
	CUMC	9.31 (pass)	0.29 (fail)	1 (pass)	NA (not evaluated)	No
	Clinformatics	1.3 (pass)	0.08 (pass)	1 (pass)	0.35 (pass)	Yes
	IQVIA	1.13 (pass)	0.12 (pass)	1 (pass)	0.08 (pass)	Yes
	JHME	4.3 (pass)	0.08 (pass)	1 (pass)	NA (not evaluated)	Yes
	MDCD	1.94 (pass)	0.1 (pass)	1 (pass)	0.08 (pass)	Yes
	MDCR	1.87 (pass)	0.02 (pass)	1 (pass)	0.2 (pass)	Yes
	OHSU	30.67 (pass)	0.29 (fail)	1 (pass)	NA (not evaluated)	No
	Optum EHR	1.54 (pass)	0.35 (fail)	1 (pass)	1 (pass)	No
	PharMetrics	1.31 (pass)	0.06 (pass)	1 (pass)	0.25 (pass)	Yes
	STARR	11.19 (pass)	0.24 (pass)	0.34 (pass)	0.95 (pass)	Yes
	USC	17.83 (pass)	0.05 (pass)	0.01 (fail)	NA (not evaluated)	No
	VA	5.28 (pass)	0.1 (pass)	1 (pass)	0.72 (pass)	Yes
	WU	5.19 (pass)	0.05 (pass)	1 (pass)	0.88 (pass)	Yes
**Glipizide**	CCAE	1.53 (pass)	0.05 (pass)	1 (pass)	0.13 (pass)	Yes
	CUMC	11.24 (pass)	0.29 (fail)	1 (pass)	0.82 (pass)	No
	Clinformatics	1.21 (pass)	0.08 (pass)	1 (pass)	0.33 (pass)	Yes
	IQVIA	1.1 (pass)	0.12 (pass)	1 (pass)	0 (fail)	No
	JHME	4.3 (pass)	0.15 (pass)	1 (pass)	NA (not evaluated)	Yes
	MDCD	1.78 (pass)	0.1 (pass)	1 (pass)	0.91 (pass)	Yes
	MDCR	1.87 (pass)	0.02 (pass)	1 (pass)	0.58 (pass)	Yes
	OHSU	6.74 (pass)	0.29 (fail)	1 (pass)	NA (not evaluated)	No
	Optum EHR	1.5 (pass)	0.35 (fail)	1 (pass)	0.99 (pass)	No
	PharMetrics	1.28 (pass)	0.06 (pass)	1 (pass)	0.36 (pass)	Yes
	STARR	5.73 (pass)	0.24 (pass)	0.34 (pass)	0.28 (pass)	Yes
	USC	18.5 (pass)	0.05 (pass)	0.01 (fail)	NA (not evaluated)	No
	VA	1.2 (pass)	0.1 (pass)	1 (pass)	0.97 (pass)	Yes

© 2025 Cai CX et al. JAMA Ophthalmology.

	WU	4.31 (pass)	0.05 (pass)	1 (pass)	NA (not evaluated)	Yes

* Only databases that passed all study diagnostics contributed to the analysis.

Abbreviations: NAION = non-arteritic anterior ischemic optic neuropathy, MDRR = minimum detectable relative risk, EASE = Expected Absolute Systematic Error

Database abbreviations:
CCAE = Merative MarketScan Commercial Claims and Encounters Database
Clinformatics = Optum's de-identified Clinformatics Data Mart Database
CUMC = Columbia University Medical Center
IQVIA = IQVIA Open Claims
JHME = Johns Hopkins Medical Enterprise
MDCD = Merative MarketScan Multi-State Medicaid Database
MDCR = Merative MarketScan Medicare Supplemental and Coordination of Benefits Database
OHSU = Oregon Health & Science University
Optum EHR = Optum de-identified Electronic Health Record data set
PharMetrics = PharMetrics Plus
STARR = Stanford University
USC = Keck Medical Center of University of Southern California
VA = Department of Veterans Affairs
WashU = Washington University in St. Louis

© 2025 Cai CX et al. JAMA Ophthalmology.

eTable 8: Results from the study diagnostics for the self-controlled case-series analysis for the “specific” NAION definition.

Exposure	Database	MDRR (MDRR diagnostic)	EASE (EASE diagnostic)	Time trend (time trend diagnostic)	Pre-exposure (pre-exposure diagnostic)	All Diagnostics Passed*
**Semaglutide**	CCAE	1.67 (pass)	0.05 (pass)	1 (pass)	0.23 (pass)	Yes
	CUMC	66.91 (pass)	0.29 (fail)	0.02 (fail)	NA (not evaluated)	No
	Clinformatics	1.52 (pass)	0.08 (pass)	1 (pass)	0.59 (pass)	Yes
	IQVIA	1.26 (pass)	0.12 (pass)	1 (pass)	0.13 (pass)	Yes
	JHME	5.84 (pass)	0.06 (pass)	0.25 (pass)	NA (not evaluated)	Yes
	MDCD	7.08 (pass)	0.1 (pass)	0.25 (pass)	NA (not evaluated)	Yes
	MDCR	3.59 (pass)	0.02 (pass)	1 (pass)	0.67 (pass)	Yes
	OHSU	16.9 (pass)	0.29 (fail)	0.02 (fail)	NA (not evaluated)	No
	Optum EHR	2.12 (pass)	0.35 (fail)	1 (pass)	0.4 (pass)	No
	PharMetrics	1.45 (pass)	0.06 (pass)	1 (pass)	0.17 (pass)	Yes
	STARR	29.14 (pass)	0.24 (pass)	0.05 (pass)	NA (not evaluated)	Yes
	USC	22026.47 (pass)	0.05 (pass)	0 (fail)	NA (not evaluated)	No
	VA	1.5 (pass)	0.1 (pass)	1 (pass)	0.29 (pass)	Yes
	WU	9.53 (pass)	0.05 (pass)	1 (pass)	0.88 (pass)	Yes
**Dulaglutide**	CCAE	1.86 (pass)	0.05 (pass)	1 (pass)	NA (not evaluated)	Yes
	CUMC	18.18 (pass)	0.29 (fail)	0.02 (fail)	NA (not evaluated)	No
	Clinformatics	1.53 (pass)	0.08 (pass)	1 (pass)	0.04 (fail)	No
	IQVIA	1.26 (pass)	0.12 (pass)	1 (pass)	0.16 (pass)	Yes
	JHME	6.5 (pass)	0.04 (pass)	0.25 (pass)	NA (not evaluated)	Yes
	MDCD	2.5 (pass)	0.1 (pass)	0.28 (pass)	0.76 (pass)	Yes
	MDCR	3.56 (pass)	0.02 (pass)	1 (pass)	NA (not evaluated)	Yes
	OHSU	16.45 (pass)	0.29 (fail)	0.02 (fail)	NA (not evaluated)	No
	Optum EHR	2.09 (pass)	0.35 (fail)	1 (pass)	0.64 (pass)	No
	PharMetrics	1.48 (pass)	0.06 (pass)	1 (pass)	0.69 (pass)	Yes
	STARR	65.04 (pass)	0.24 (pass)	0.05 (pass)	NA (not evaluated)	Yes

© 2025 Cai CX et al. JAMA Ophthalmology.

	USC	22026.47 (pass)	0.05 (pass)	0 (fail)	NA (not evaluated)	No
	VA	2.18 (pass)	0.1 (pass)	1 (pass)	NA (not evaluated)	Yes
	WU	7.59 (pass)	0.05 (pass)	1 (pass)	NA (not evaluated)	Yes
**Exenatide**	CCAE	4.45 (pass)	0.05 (pass)	1 (pass)	NA (not evaluated)	Yes
	CUMC	NA (pass)	0.29 (fail)	0.02 (fail)	NA (not evaluated)	No
	Clinformatics	2.96 (pass)	0.08 (pass)	1 (pass)	NA (not evaluated)	Yes
	IQVIA	1.74 (pass)	0.12 (pass)	1 (pass)	0.21 (pass)	Yes
	JHME	22026.47 (pass)	0.13 (pass)	0.25 (pass)	NA (not evaluated)	Yes
	MDCD	7.53 (pass)	0.1 (pass)	0.3 (pass)	NA (not evaluated)	Yes
	MDCR	193.68 (pass)	0.02 (pass)	1 (pass)	NA (not evaluated)	Yes
	OHSU	533.91 (pass)	0.29 (fail)	0.02 (fail)	NA (not evaluated)	No
	Optum EHR	3.94 (pass)	0.35 (fail)	1 (pass)	NA (not evaluated)	No
	PharMetrics	2.77 (pass)	0.06 (pass)	1 (pass)	NA (not evaluated)	Yes
	STARR	NA (pass)	0.24 (pass)	0.05 (pass)	NA (not evaluated)	Yes
	USC	NA (pass)	0.05 (pass)	0 (fail)	NA (not evaluated)	No
	VA	9.75 (pass)	0.1 (pass)	1 (pass)	NA (not evaluated)	Yes
	WU	NA (pass)	0.05 (pass)	1 (pass)	NA (not evaluated)	Yes
**Empagliflozin**	CCAE	1.71 (pass)	0.05 (pass)	1 (pass)	0.41 (pass)	Yes
	CUMC	9.9 (pass)	0.29 (fail)	0.02 (fail)	NA (not evaluated)	No
	Clinformatics	1.44 (pass)	0.08 (pass)	1 (pass)	0.5 (pass)	Yes
	IQVIA	1.22 (pass)	0.12 (pass)	1 (pass)	0.22 (pass)	Yes
	JHME	11.32 (pass)	0.06 (pass)	0.25 (pass)	NA (not evaluated)	Yes
	MDCD	2.94 (pass)	0.1 (pass)	0.15 (pass)	0.95 (pass)	Yes
	MDCR	2.69 (pass)	0.02 (pass)	1 (pass)	0.25 (pass)	Yes

© 2025 Cai CX et al. JAMA Ophthalmology.

	OHSU	23.55 (pass)	0.29 (fail)	0.02 (fail)	NA (not evaluated)	No
	Optum EHR	1.91 (pass)	0.35 (fail)	1 (pass)	0.39 (pass)	No
	PharMetrics	1.42 (pass)	0.06 (pass)	1 (pass)	0.33 (pass)	Yes
	STARR	21.08 (pass)	0.24 (pass)	0.05 (pass)	NA (not evaluated)	Yes
	USC	NA (pass)	0.05 (pass)	0 (fail)	NA (not evaluated)	No
	VA	1.38 (pass)	0.1 (pass)	1 (pass)	0.37 (pass)	Yes
	WU	5.33 (pass)	0.05 (pass)	1 (pass)	NA (not evaluated)	Yes
**Sitagliptin**	CCAE	1.81 (pass)	0.05 (pass)	1 (pass)	0.2 (pass)	Yes
	CUMC	12.88 (pass)	0.29 (fail)	0.02 (fail)	NA (not evaluated)	No
	Clinformatics	1.46 (pass)	0.08 (pass)	1 (pass)	0.01 (fail)	No
	IQVIA	1.2 (pass)	0.12 (pass)	1 (pass)	0.03 (fail)	No
	JHME	7.77 (pass)	0.08 (pass)	0.25 (pass)	NA (not evaluated)	Yes
	MDCD	2.61 (pass)	0.1 (pass)	0.3 (pass)	0.05 (fail)	No
	MDCR	2.18 (pass)	0.02 (pass)	1 (pass)	0.28 (pass)	Yes
	OHSU	30.67 (pass)	0.29 (fail)	0.02 (fail)	NA (not evaluated)	No
	Optum EHR	1.71 (pass)	0.35 (fail)	1 (pass)	0.99 (pass)	No
	PharMetrics	1.45 (pass)	0.06 (pass)	1 (pass)	0.28 (pass)	Yes
	STARR	28.7 (pass)	0.24 (pass)	0.05 (pass)	NA (not evaluated)	Yes
	USC	100.23 (pass)	0.05 (pass)	0 (fail)	NA (not evaluated)	No
	VA	30.94 (pass)	0.1 (pass)	1 (pass)	NA (not evaluated)	Yes
	WU	5.78 (pass)	0.05 (pass)	1 (pass)	0.88 (pass)	Yes
**Glipizide**	CCAE	1.7 (pass)	0.05 (pass)	1 (pass)	0.18 (pass)	Yes
	CUMC	18.47 (pass)	0.29 (fail)	0.02 (fail)	0.82 (pass)	No
	Clinformatics	1.3 (pass)	0.08 (pass)	1 (pass)	0.12 (pass)	Yes
	IQVIA	1.16 (pass)	0.12 (pass)	1 (pass)	0.01 (fail)	No
	JHME	8.97 (pass)	0.15 (pass)	0.25 (pass)	NA (not evaluated)	Yes
	MDCD	2.48 (pass)	0.1 (pass)	0.3 (pass)	0.98 (pass)	Yes
	MDCR	2.29 (pass)	0.02 (pass)	1 (pass)	0.85 (pass)	Yes
	OHSU	13.09 (pass)	0.29 (fail)	0.02 (fail)	NA (not evaluated)	No
	Optum EHR	1.65 (pass)	0.35 (fail)	1 (pass)	0.99 (pass)	No

© 2025 Cai CX et al. JAMA Ophthalmology.

	PharMetrics	1.42 (pass)	0.06 (pass)	1 (pass)	0.68 (pass)	Yes
	STARR	13.38 (pass)	0.24 (pass)	0.05 (pass)	NA (not evaluated)	Yes
	USC	37.58 (pass)	0.05 (pass)	0 (fail)	NA (not evaluated)	No
	VA	1.3 (pass)	0.1 (pass)	1 (pass)	0.66 (pass)	Yes
	WU	7.47 (pass)	0.05 (pass)	1 (pass)	NA (not evaluated)	Yes

* Only databases that passed all study diagnostics contributed to the analysis.

Abbreviations: NAION = non-arteritic anterior ischemic optic neuropathy, MDRR = minimum detectable relative risk, EASE = Expected Absolute Systematic Error

Database abbreviations:
CCAE = Merative MarketScan Commercial Claims and Encounters Database
Clinformatics = Optum's de-identified Clinformatics Data Mart Database
CUMC = Columbia University Medical Center
IQVIA = IQVIA Open Claims
JHME = Johns Hopkins Medical Enterprise
MDCD = Merative MarketScan Multi-State Medicaid Database
MDCR = Merative MarketScan Medicare Supplemental and Coordination of Benefits Database
OHSU = Oregon Health & Science University
Optum EHR = Optum de-identified Electronic Health Record data set
PharMetrics = PharMetrics Plus
STARR = Stanford University
USC = Keck Medical Center of University of Southern California
VA = Department of Veterans Affairs
WashU = Washington University in St. Louis

© 2025 Cai CX et al. JAMA Ophthalmology.

eTable 9: Performance characteristics of definition algorithms for the “sensitive” and “specific” definitions of NAION.

NAION definition	Database	Sensitivity (95% CI)	Positive Predictive Value (95% CI)	Specificity (95% CI)	Negative Predictive Value (95% CI)
“Sensitive” Definition	CCAE	0.649 (0.606 - 0.690)	0.701 (0.658 - 0.741)	1.000 (1.000 - 1.000)	1.000 (1.000 - 1.000)
	MDCD	0.717 (0.682 - 0.750)	0.650 (0.615 - 0.683)	1.000 (1.000 - 1.000)	1.000 (1.000 - 1.000)
	MDCR	0.691 (0.678 - 0.704)	0.814 (0.802 - 0.826)	1.000 (1.000 - 1.000)	0.999 (0.999 - 0.999)
	Optum EHR	0.764 (0.719 - 0.805)	0.652 (0.606 - 0.696)	1.000 (1.000 - 1.000)	1.000 (1.000 - 1.000)
	Clinformatics	0.808 (0.789 - 0.827)	0.726 (0.706 - 0.746)	1.000 (1.000 - 1.000)	1.000 (1.000 - 1.000)
“Specific” Definition	CCAE	0.341 (0.300 - 0.384)	0.911 (0.861 - 0.947)	1.000 (1.000 - 1.000)	1.000 (1.000 - 1.000)
	MDCD	0.274 (0.240 - 0.309)	0.851 (0.796 - 0.896)	1.000 (1.000 - 1.000)	1.000 (1.000 - 1.000)
	MDCR	0.248 (0.235 - 0.260)	0.957 (0.943 - 0.968)	1.000 (1.000 - 1.000)	0.998 (0.998 - 0.998)
	Optum EHR	0.377 (0.328 - 0.429)	0.773 (0.706 - 0.832)	1.000 (1.000 - 1.000)	1.000 (1.000 - 1.000)
	Clinformatics	0.406 (0.381 - 0.431)	0.920 (0.898 - 0.939)	1.000 (1.000 - 1.000)	0.999 (0.999 - 1.000)

Abbreviations: NAION = non-arteritic anterior ischemic optic neuropathy, CI = Confidence Interval

Database Abbreviations:

CCAE = Merative MarketScan Commercial Claims and Encounters Database
MDCD = Merative MarketScan Multi-State Medicaid Database
MDCR = Merative MarketScan Medicare Supplemental and Coordination of Benefits Database
Optum EHR = Optum de-identified Electronic Health Record data set
Clinformatics = Optum’s de-identified Clinformatics Data Mart Database

© 2025 Cai CX et al. JAMA Ophthalmology.

eTable 10: Baseline characteristics of patients in each exposure cohort semaglutide, dulaglutide, exenatide, empagliflozin, sitagliptin, glipizide during the study period in all databases.

Database	Covariate	Semaglutide (GLP-1 RA)	Dulaglutide (GLP-1 RA)	Exenatide (GLP-1 RA)	Empagliflozin (SGLT2 inhibitor)	Sitagliptin (DPP4 inhibitor)	Glipizide (sulfonylurea)
**CCAE**		N=50281	N=16473	N=1037	N=27613	N=22447	N=25434
	Age (years)*						
	≤29	1107 (2)	342 (2)	29 (2)	288 (1)	245 (1)	324 (1)
	30-49	17366 (35)	5616 (34)	366 (35)	7276 (26)	6263 (28)	7519 (30)
	50-69	31808 (63)	10515 (64)	645 (62)	20049 (73)	15939 (71)	17591 (69)
	Sex						
	Female	30885 (61)	9125 (55)	594 (57)	10611 (38)	10166 (45)	10697 (42)
	Male	19396 (39)	7348 (45)	443 (43)	17002 (62)	12281 (55)	14737 (58)
	DCSI (SD)&	1.89 (1.56)	2.09 (1.61)	2.13 (1.69)	2.24 (1.68)	2.2 (1.75)	2.16 (1.67)
	CCI (SD)&	3.14 (1.87)	3.27 (1.87)	3.2 (1.76)	3.41 (1.96)	3.28 (1.93)	3.25 (1.9)
	Essential hypertension	33449 (67)	11190 (68)	722 (70)	19229 (70)	15474 (69)	16826 (66)
	Hyperlipidemia	34128 (68)	11287 (69)	708 (68)	20610 (75)	16292 (73)	17154 (67)
	Obstructive sleep apnea	12216 (24)	3728 (23)	241 (23)	5210 (19)	3505 (16)	3821 (15)
	Chronic kidney disease	1648 (3)	585 (4)	32 (3)	1200 (4)	804 (4)	1060 (4)
	Anemia	5808 (12)	1611 (10)	92 (9)	2441 (9)	2083 (9)	2115 (8)
	Interferon use	8 (0)	5 (0)	0 (0)	<5 (0)	5 (0)	5 (0)
	Phosphodiesterase inhibitor use	<5 (0)	0 (0)	0 (0)	<5 (0)	0 (0)	<5 (0)
**Clinformatics**		**N=43620**	**N=14923**	**N=1414**	**N=43302**	**N=19581**	**N=44092**
	Age (years)*						
	≤29	453 (1)	168 (1)	23 (1)	165 (0)	29 (0)	174 (0)
	30-49	8110 (19)	3095 (21)	315 (22)	5122 (12)	875 (4)	4478 (10)
	50-69	24473 (56)	8238 (55)	832 (59)	21614 (50)	7292 (37)	20330 (46)
	≥70	10586 (24)	3426 (23)	248 (18)	16401 (38)	11389 (58)	19111 (43)
	Race						
	Asian	1179 (3)	332 (2)	37 (3)	2333 (5)	961 (5)	2008 (5)
	Black or African American	5748 (13)	1939 (13)	166 (12)	5228 (12)	3020 (15)	5458 (12)
	White	28635 (66)	9976 (67)	900 (64)	27337 (63)	11283 (58)	26464 (60)
	Ethnicity						
	Hispanic or Latino	4920 (11)	1703 (11)	228 (16)	5329 (12)	2892 (15)	6836 (16)

© 2025 Cai CX et al. JAMA Ophthalmology.

	Not Hispanic or Latino	35562 (82)	12247 (82)	1103 (78)	34898 (81)	15264 (78)	33930 (77)
	Sex						
	Female	26699 (61)	8343 (56)	797 (56)	17872 (41)	10683 (55)	20587 (47)
	Male	16921 (39)	6580 (44)	617 (44)	25430 (59)	8898 (45)	23505 (53)
	DCSI (SD)&	2.57 (1.97)	2.64 (1.97)	2.72 (2.08)	3.25 (2.25)	3.57 (2.39)	3.26 (2.31)
	CCI (SD)&	4.04 (2.37)	4.05 (2.33)	3.92 (2.23)	4.69 (2.67)	5.03 (2.71)	4.64 (2.68)
	Essential hypertension	33537 (77)	11420 (77)	1087 (77)	34789 (80)	16441 (84)	35070 (80)
	Hyperlipidemia	34056 (78)	11393 (76)	1079 (76)	35942 (83)	16342 (83)	34918 (79)
	Obstructive sleep apnea	12079 (28)	3729 (25)	348 (25)	8654 (20)	2905 (15)	6142 (14)
	Chronic kidney disease	4776 (11)	1670 (11)	134 (9)	7358 (17)	4142 (21)	8067 (18)
	Anemia	6373 (15)	1859 (12)	172 (12)	7048 (16)	3888 (20)	7178 (16)
	Interferon use	6 (0)	5 (0)	0 (0)	7 (0)	<5 (0)	9 (0)
	Phosphodiesterase inhibitor use	0 (0)	0 (0)	0 (0)	6 (0)	<5 (0)	<5 (0)
**CUMC**		**N=1814**	**N=330**	**N=18**	**N=1395**	**N=967**	**N=495**
	Age (years)*						
	≤29	51 (3)	15 (0)	5 (0)	15 (0)	10 (0)	5 (1)
	30-49	396 (22)	67 (19)	5 (0)	140 (10)	88 (9)	57 (11)
	50-69	1041 (57)	188 (57)	11 (33)	695 (50)	497 (51)	230 (46)
	≥70	329 (18)	75 (21)	15 (0)	558 (40)	386 (39)	209 (41)
	Race						
	American Indian or Alaska Native	7 (0)	<5 (0)	<5 (0)	<5 (0)	<5 (0)	<5 (0)
	Asian	53 (3)	7 (2)	0 (0)	54 (4)	40 (4)	16 (3)
	Black or African American	331 (18)	58 (18)	<5 (0)	239 (17)	183 (19)	109 (22)
	Native Hawaiian or Other Pacific Islander	<5 (0)	0 (0)	0 (0)	<5 (0)	0 (0)	0 (0)
	White	764 (42)	140 (42)	12 (67)	556 (40)	328 (34)	170 (34)
	Ethnicity						
	Hispanic or Latino	418 (23)	77 (23)	<5 (0)	356 (26)	281 (29)	132 (27)
	Not Hispanic or Latino	1078 (59)	189 (57)	12 (67)	781 (56)	505 (52)	262 (53)
	Sex						
	Female	1200 (66)	200 (61)	15 (83)	614 (44)	523 (54)	251 (51)
	Male	614 (34)	130 (39)	<5 (0)	781 (56)	444 (46)	244 (49)

© 2025 Cai CX et al. JAMA Ophthalmology.

	DCSI (SD)&	2 (1.74)	2.05 (1.74)	2.39 (2.43)	2.84 (2.13)	2.43 (2.13)	2.27 (2.03)
	CCI (SD)&	3.46 (2.55)	3.41 (2.23)	3.5 (2.66)	4.13 (2.91)	3.87 (2.93)	3.75 (2.65)
	Essential hypertension	1058 (58)	156 (47)	10 (56)	934 (67)	574 (59)	272 (55)
	Hyperlipidemia	1096 (60)	168 (51)	12 (67)	880 (63)	499 (52)	226 (46)
	Obstructive sleep apnea	303 (17)	34 (10)	<5 (0)	117 (8)	55 (6)	22 (4)
	Chronic kidney disease	68 (4)	17 (5)	<5 (0)	132 (9)	71 (7)	27 (5)
	Anemia	234 (13)	25 (8)	0 (0)	145 (10)	76 (8)	37 (7)
	Phosphodiesterase inhibitor use	<5 (0)	<5 (0)	0 (0)	<5 (0)	0 (0)	0 (0)
**IQVIA**		**N=582835**	**N=237440**	**N=18704**	**N=481310**	**N=365524**	**N=589384**
	Age (years)*						
	≤29	13285 (2)	6276 (3)	609 (3)	4690 (1)	4192 (1)	8004 (1)
	30-49	147798 (25)	64566 (27)	5625 (30)	73911 (15)	60854 (17)	105316 (18)
	50-69	339925 (58)	135589 (57)	10255 (55)	271269 (56)	198133 (54)	316186 (54)
	≥70	81827 (14)	31009 (13)	2215 (12)	131440 (27)	102345 (28)	159878 (27)
	Sex						
	Female	370087 (63)	142671 (60)	11297 (60)	209238 (43)	190586 (52)	286761 (49)
	Male	212747 (37)	94768 (40)	7407 (40)	272072 (57)	174937 (48)	302623 (51)
	DCSI (SD)&	2.06 (1.91)	2.17 (1.95)	2.26 (2.04)	2.69 (2.19)	2.66 (2.24)	2.59 (2.19)
	CCI (SD)&	3.32 (2.17)	3.36 (2.18)	3.27 (2.11)	3.85 (2.46)	3.7 (2.41)	3.65 (2.38)
	Essential hypertension	279355 (48)	114084 (48)	8974 (48)	247904 (52)	187520 (51)	286889 (49)
	Hyperlipidemia	243844 (42)	96444 (41)	7335 (39)	222884 (46)	166243 (45)	242790 (41)
	Obstructive sleep apnea	97869 (17)	37920 (16)	3008 (16)	57126 (12)	34021 (9)	48928 (8)
	Chronic kidney disease	23405 (4)	9988 (4)	707 (4)	34951 (7)	23990 (7)	42050 (7)
	Anemia	38454 (7)	14630 (6)	1218 (7)	33403 (7)	28510 (8)	40391 (7)
	Interferon use	96 (0)	47 (0)	<5 (0)	63 (0)	75 (0)	93 (0)
	Phosphodiesterase inhibitor use	5 (0)	6 (0)	0 (0)	80 (0)	22 (0)	24 (0)
**JHME**		**N=1626**	**N=621**	**N=19**	**N=1231**	**N=936**	**N=1039**
	Age (years)*						
	≤29	31 (2)	16 (1)	5 (0)	17 (1)	15 (1)	13 (1)

© 2025 Cai CX et al. JAMA Ophthalmology.

	30-49	468 (29)	187 (30)	15 (0)	167 (14)	176 (19)	201 (19)
	50-69	938 (58)	355 (57)	15 (0)	700 (57)	500 (53)	610 (59)
	≥70	189 (12)	72 (10)	5 (0)	354 (29)	251 (27)	215 (21)
	Race						
	American Indian or Alaska Native	12 (1)	<5 (0)	0 (0)	7 (1)	<5 (0)	5 (0)
	Asian	<5 (0)	<5 (0)	0 (0)	5 (0)	10 (1)	9 (1)
	Asian Indian	19 (1)	5 (1)	0 (0)	17 (1)	20 (2)	17 (2)
	Black or African American	642 (39)	239 (38)	<5 (0)	404 (33)	288 (31)	370 (36)
	Chinese	6 (0)	<5 (0)	0 (0)	9 (1)	6 (1)	6 (1)
	Filipino	12 (1)	<5 (0)	0 (0)	9 (1)	5 (1)	10 (1)
	Korean	6 (0)	<5 (0)	0 (0)	<5 (0)	7 (1)	5 (0)
	Native Hawaiian or Other Pacific Islander	<5 (0)	<5 (0)	0 (0)	0 (0)	<5 (0)	0 (0)
	Other Pacific Islander	5 (0)	0 (0)	0 (0)	0 (0)	<5 (0)	<5 (0)
	Vietnamese	<5 (0)	0 (0)	0 (0)	0 (0)	<5 (0)	<5 (0)
	White	779 (48)	326 (52)	15 (79)	644 (52)	495 (53)	452 (44)
	Japanese	0 (0)	0 (0)	0 (0)	<5 (0)	<5 (0)	0 (0)
	Ethnicity						
	Hispanic or Latino	<5 (0)	<5 (0)	0 (0)	6 (0)	8 (1)	20 (2)
	Not Hispanic or Latino	1505 (93)	580 (93)	18 (95)	1142 (93)	869 (93)	903 (87)
	Sex						
	Female	1042 (64)	385 (62)	14 (74)	508 (41)	500 (53)	576 (55)
	Male	584 (36)	236 (38)	5 (26)	723 (59)	436 (47)	463 (45)
	DCSI (SD)&	1.54 (1.38)	1.59 (1.63)	0.79 (1)	2.16 (1.82)	1.51 (1.4)	1.73 (1.5)
	CCI (SD)&	2.82 (1.8)	2.84 (1.92)	1.89 (1.45)	3.52 (2.29)	2.96 (2.02)	3.06 (1.9)
	Essential hypertension	1052 (65)	407 (66)	16 (84)	876 (71)	642 (69)	700 (67)
	Hyperlipidemia	973 (60)	353 (57)	14 (74)	799 (65)	597 (64)	625 (60)
	Obstructive sleep apnea	313 (19)	107 (17)	<5 (0)	206 (17)	103 (11)	108 (10)
	Chronic kidney disease	85 (5)	41 (7)	<5 (0)	163 (13)	66 (7)	70 (7)
	Anemia	177 (11)	67 (11)	<5 (0)	162 (13)	105 (11)	122 (12)
	Phosphodiesterase inhibitor use	0 (0)	0 (0)	0 (0)	<5 (0)	0 (0)	0 (0)
**MDCD**		**N=2120**	**N=6460**	**N=744**	**N=5672**	**N=5111**	**N=8229**

© 2025 Cai CX et al. JAMA Ophthalmology.

	Age (years)*						
	≤29	186 (9)	492 (8)	61 (8)	214 (4)	231 (5)	339 (4)
	30-49	1014 (48)	3064 (47)	361 (49)	1910 (34)	1784 (35)	3139 (38)
	50-69	880 (42)	2838 (44)	314 (42)	3320 (59)	2752 (54)	4394 (53)
	≥70	48 (2)	70 (1)	15 (0)	232 (4)	347 (7)	359 (4)
	Race						
	Black or African American	537 (25)	1380 (21)	208 (28)	1330 (23)	1324 (26)	2256 (27)
	White	1181 (56)	3835 (59)	404 (54)	3069 (54)	2661 (52)	4136 (50)
	Ethnicity						
	Hispanic or Latino	79 (4)	645 (10)	68 (9)	402 (7)	285 (6)	479 (6)
	Sex						
	Female	1632 (77)	4644 (72)	546 (73)	3070 (54)	3201 (63)	4930 (60)
	Male	488 (23)	1816 (28)	198 (27)	2602 (46)	1910 (37)	3299 (40)
	DCSI (SD)&	2.3 (1.91)	2.39 (1.9)	2.76 (2.07)	3.01 (2.17)	2.98 (2.2)	2.91 (2.09)
	CCI (SD)&	3.75 (2.3)	3.82 (2.26)	4.08 (2.32)	4.44 (2.59)	4.39 (2.59)	4.27 (2.5)
	Essential hypertension	1400 (66)	4324 (67)	520 (70)	4204 (74)	3763 (74)	6097 (74)
	Hyperlipidemia	1214 (57)	3594 (56)	414 (56)	3689 (65)	3234 (63)	5062 (62)
	Obstructive sleep apnea	615 (29)	1936 (30)	231 (31)	1200 (21)	932 (18)	1440 (17)
	Chronic kidney disease	88 (4)	305 (5)	51 (7)	460 (8)	365 (7)	591 (7)
	Anemia	376 (18)	817 (13)	117 (16)	765 (13)	769 (15)	1168 (14)
	Interferon use	0 (0)	<5 (0)	0 (0)	<5 (0)	<5 (0)	<5 (0)
	Phosphodiesterase inhibitor use	0 (0)	0 (0)	0 (0)	6 (0)	<5 (0)	0 (0)
**MDCR**		**N=3982**	**N=1473**	**N=96**	**N=5997**	**N=3550**	**N=3607**
	Age (years)*						
	30-49	5 (0)	5 (0)	0 (0)	10 (0)	0 (0)	0 (0)
	50-69	1482 (37)	546 (37)	42 (44)	1483 (25)	893 (25)	906 (25)
	≥70	2498 (63)	932 (63)	58 (55)	4512 (75)	2661 (75)	2705 (75)
	Sex						
	Female	2454 (62)	847 (58)	50 (52)	2517 (42)	1748 (49)	1720 (48)
	Male	1528 (38)	626 (42)	46 (48)	3480 (58)	1802 (51)	1887 (52)
	DCSI (SD)&	2.85 (2.14)	3.16 (2.3)	3.33 (2.45)	3.95 (2.53)	3.68 (2.56)	3.62 (2.51)
	CCI (SD)&	4.47 (2.46)	4.67 (2.52)	4.6 (2.72)	5.49 (2.82)	5.05 (2.72)	5.07 (2.82)
	Essential hypertension	3315 (83)	1217 (83)	81 (84)	5181 (86)	2974 (84)	2980 (83)
	Hyperlipidemia	3299 (83)	1227 (83)	75 (78)	5099 (85)	2905 (82)	2868 (80)

© 2025 Cai CX et al. JAMA Ophthalmology.

	Obstructive sleep apnea	1325 (33)	453 (31)	29 (30)	1402 (23)	605 (17)	612 (17)
	Chronic kidney disease	629 (16)	239 (16)	10 (10)	1475 (25)	621 (17)	745 (21)
	Anemia	691 (17)	223 (15)	18 (19)	1304 (22)	681 (19)	698 (19)
	Interferon use	0 (0)	0 (0)	0 (0)	<5 (0)	<5 (0)	0 (0)
	Phosphodiesterase inhibitor use	0 (0)	0 (0)	0 (0)	<5 (0)	0 (0)	0 (0)
**OHSU**		**N=604**	**N=194**	**N=32**	**N=578**	**N=142**	**N=656**
	Age (years)*						
	≤29	20 (2)	15 (0)	5 (0)	15 (0)	5 (0)	20 (2)
	30-49	184 (30)	51 (24)	15 (16)	88 (15)	31 (18)	156 (24)
	50-69	315 (52)	105 (54)	24 (59)	267 (46)	66 (46)	316 (48)
	≥70	93 (14)	37 (16)	10 (0)	220 (37)	48 (27)	171 (25)
	Race						
	American Indian or Alaska Native	9 (1)	<5 (0)	0 (0)	9 (2)	<5 (0)	17 (3)
	Asian	19 (3)	9 (5)	<5 (0)	38 (7)	8 (6)	35 (5)
	Black or African American	34 (6)	6 (3)	<5 (0)	22 (4)	<5 (0)	30 (5)
	Middle Eastern or North African	5 (1)	0 (0)	0 (0)	<5 (0)	0 (0)	<5 (0)
	Native Hawaiian or Other Pacific Islander	5 (1)	<5 (0)	<5 (0)	6 (1)	<5 (0)	7 (1)
	Other Race	53 (9)	10 (5)	<5 (0)	36 (6)	10 (7)	47 (7)
	Unknown	27 (4)	8 (4)	<5 (0)	20 (3)	8 (6)	25 (4)
	White	452 (75)	157 (81)	25 (78)	446 (77)	110 (77)	494 (75)
	Ethnicity						
	Hispanic or Latino	65 (11)	17 (9)	<5 (0)	50 (9)	16 (11)	84 (13)
	Not Hispanic or Latino	479 (79)	162 (84)	28 (88)	479 (83)	111 (78)	529 (81)
	Sex						
	Female	404 (67)	117 (60)	18 (56)	254 (44)	83 (58)	331 (50)
	Male	200 (33)	77 (40)	14 (44)	324 (56)	59 (42)	325 (50)
	DCSI (SD)&	1.75 (1.78)	1.66 (1.79)	1.44 (1.72)	2.42 (1.92)	1.73 (1.58)	1.75 (1.6)
	CCI (SD)&	3.13 (2.07)	3.22 (2.41)	2.94 (1.83)	3.86 (2.38)	3.08 (1.98)	3.17 (2.21)
	Essential hypertension	319 (53)	73 (38)	14 (44)	336 (58)	60 (42)	281 (43)
	Hyperlipidemia	287 (48)	74 (38)	11 (34)	324 (56)	61 (43)	237 (36)
	Obstructive sleep apnea	162 (27)	29 (15)	6 (19)	104 (18)	11 (8)	68 (10)

© 2025 Cai CX et al. JAMA Ophthalmology.

	Chronic kidney disease	35 (6)	<5 (0)	0 (0)	72 (12)	10 (7)	41 (6)
	Anemia	61 (10)	15 (8)	<5 (0)	50 (9)	9 (6)	55 (8)
	Phosphodiesterase inhibitor use	0 (0)	0 (0)	0 (0)	0 (0)	<5 (0)	0 (0)
**Optum EHR**		**N=38731**	**N=18323**	**N=1877**	**N=34553**	**N=35376**	**N=45714**
	Age (years)*						
	≤29	1063 (3)	520 (3)	55 (3)	346 (1)	457 (1)	663 (1)
	30-49	10889 (28)	5400 (29)	595 (32)	6200 (18)	6809 (19)	9368 (20)
	50-69	21962 (57)	10357 (57)	1027 (55)	19815 (57)	19399 (55)	24788 (54)
	≥70	4817 (12)	2046 (11)	202 (10)	8192 (24)	8711 (25)	10895 (24)
	Race						
	Asian	956 (2)	360 (2)	28 (1)	1416 (4)	1414 (4)	1516 (3)
	Black or African American	6001 (15)	2576 (14)	220 (12)	4183 (12)	4902 (14)	5856 (13)
	White	28887 (75)	13924 (76)	1491 (79)	26036 (75)	25944 (73)	33929 (74)
	Ethnicity						
	Hispanic or Latino	2485 (6)	1512 (8)	97 (5)	2343 (7)	2571 (7)	4016 (9)
	Not Hispanic or Latino	31751 (82)	15071 (82)	1611 (86)	28238 (82)	28674 (81)	37201 (81)
	Sex						
	Female	24689 (64)	10832 (59)	1117 (60)	14431 (42)	18240 (52)	20950 (46)
	Male	14042 (36)	7491 (41)	760 (40)	20122 (58)	17136 (48)	24764 (54)
	DCSI (SD)&	2.55 (1.92)	2.55 (1.92)	2.63 (1.99)	2.96 (2.12)	2.73 (2.05)	2.77 (2.07)
	CCI (SD)&	3.06 (1.96)	3.12 (1.91)	3.05 (1.81)	3.52 (2.17)	3.22 (2.04)	3.34 (2.06)
	Essential hypertension	24417 (63)	11054 (60)	1118 (60)	23056 (67)	22850 (65)	28420 (62)
	Hyperlipidemia	24324 (63)	10934 (60)	1043 (56)	23551 (68)	22667 (64)	27327 (60)
	Obstructive sleep apnea	7905 (20)	3378 (18)	357 (19)	5280 (15)	4136 (12)	5175 (11)
	Chronic kidney disease	2010 (5)	939 (5)	64 (3)	2757 (8)	2347 (7)	3648 (8)
	Anemia	3424 (9)	1378 (8)	136 (7)	2756 (8)	2963 (8)	3512 (8)
	Interferon use	11 (0)	9 (0)	0 (0)	8 (0)	8 (0)	11 (0)
	Phosphodiesterase inhibitor use	<5 (0)	0 (0)	0 (0)	12 (0)	<5 (0)	7 (0)
**PharMetrics**		**N=76664**	**N=28967**	**N=1952**	**N=51910**	**N=39128**	**N=51445**
	Age (years)*						
	≤29	1581 (2)	521 (2)	46 (2)	530 (1)	456 (1)	632 (1)

© 2025 Cai CX et al. JAMA Ophthalmology.

	30-49	23806 (31)	9035 (31)	661 (34)	11400 (22)	9333 (24)	12438 (24)
	50-69	48401 (63)	18350 (63)	1180 (60)	35616 (69)	25731 (66)	33973 (66)
	≥70	2876 (4)	1061 (4)	65 (3)	4364 (8)	3608 (9)	4402 (9)
	Sex						
	Female	45528 (59)	15692 (54)	1064 (55)	19381 (37)	17608 (45)	21200 (41)
	Male	31136 (41)	13275 (46)	888 (45)	32529 (63)	21520 (55)	30245 (59)
	DCSI (SD)&	1.61 (1.34)	1.7 (1.36)	1.72 (1.41)	2.03 (1.59)	1.88 (1.58)	1.9 (1.54)
	CCI (SD)&	2.99 (1.8)	3.02 (1.78)	2.91 (1.65)	3.34 (2)	3.17 (1.96)	3.18 (1.94)
	Essential hypertension	51057 (67)	19406 (67)	1323 (68)	36887 (71)	27136 (69)	34495 (67)
	Hyperlipidemia	47662 (62)	17975 (62)	1178 (60)	36190 (70)	26513 (68)	32815 (64)
	Obstructive sleep apnea	13998 (18)	4784 (17)	314 (16)	7156 (14)	4236 (11)	4961 (10)
	Chronic kidney disease	3059 (4)	1147 (4)	83 (4)	3263 (6)	2171 (6)	3030 (6)
	Anemia	5686 (7)	1810 (6)	122 (6)	3510 (7)	2872 (7)	3456 (7)
	Interferon use	12 (0)	9 (0)	0 (0)	16 (0)	8 (0)	10 (0)
	Phosphodiesterase inhibitor use	<5 (0)	<5 (0)	0 (0)	5 (0)	0 (0)	<5 (0)
**STARR**		**N=1141**	**N=345**	**N=14**	**N=990**	**N=595**	**N=858**
	Age (years)*						
	≤29	39 (3)	18 (2)	0 (0)	13 (1)	5 (0)	14 (1)
	30-49	289 (25)	83 (24)	5 (0)	177 (18)	97 (16)	135 (16)
	50-69	628 (55)	183 (53)	20 (0)	486 (49)	289 (49)	429 (50)
	≥70	186 (16)	68 (18)	5 (0)	319 (32)	207 (35)	282 (33)
	Race						
	American Indian or Alaska Native	5 (0)	<5 (0)	0 (0)	<5 (0)	<5 (0)	<5 (0)
	Asian	253 (22)	52 (15)	<5 (0)	308 (31)	178 (30)	222 (26)
	Black or African American	93 (8)	20 (6)	<5 (0)	59 (6)	40 (7)	68 (8)
	Native Hawaiian or Other Pacific Islander	27 (2)	13 (4)	0 (0)	22 (2)	17 (3)	22 (3)
	White	527 (46)	169 (49)	5 (36)	386 (39)	217 (36)	326 (38)
	Ethnicity						
	Hispanic or Latino	197 (17)	74 (21)	<5 (0)	146 (15)	99 (17)	181 (21)
	Not Hispanic or Latino	893 (78)	257 (74)	10 (71)	782 (79)	460 (77)	644 (75)
	Sex						
	Female	698 (61)	197 (57)	10 (71)	404 (41)	310 (52)	387 (45)

© 2025 Cai CX et al. JAMA Ophthalmology.

	Male	443 (39)	148 (43)	<5 (0)	585 (59)	285 (48)	469 (55)
	DCSI (SD)&	1.96 (1.66)	2.2 (1.7)	1.79 (1.3)	2.67 (1.92)	2.37 (1.77)	2.49 (1.82)
	CCI (SD)&	3.3 (2.21)	3.5 (2.49)	2.93 (1.69)	3.96 (2.57)	3.66 (2.38)	3.67 (2.38)
	Essential hypertension	651 (57)	169 (49)	5 (36)	582 (59)	355 (60)	454 (53)
	Hyperlipidemia	730 (64)	186 (54)	6 (43)	645 (65)	370 (62)	479 (56)
	Obstructive sleep apnea	270 (24)	52 (15)	0 (0)	154 (16)	51 (9)	92 (11)
	Chronic kidney disease	52 (5)	18 (5)	<5 (0)	100 (10)	49 (8)	73 (9)
	Anemia	124 (11)	26 (8)	<5 (0)	117 (12)	63 (11)	79 (9)
**USC**		**N=197**	**N=54**	**glp-1 ra n=0**	**N=116**	**N=170**	**N=151**
	Age (years)*						
	≤29	4 (2)	0 (0)	0 (0)	2 (2)	0 (0)	4 (3)
	30-49	49 (25)	11 (20)	0 (0)	17 (15)	17 (10)	23 (15)
	50-69	115 (58)	32 (59)	0 (0)	58 (50)	78 (46)	69 (46)
	≥70	29 (15)	11 (20)	0 (0)	39 (34)	75 (44)	55 (36)
	Race						
	Asian	19 (10)	2 (4)	0 (0)	11 (9)	14 (8)	18 (12)
	Black or African American	10 (5)	4 (7)	0 (0)	5 (4)	2 (1)	6 (4)
	White	75 (38)	14 (26)	0 (0)	38 (33)	72 (42)	62 (41)
	American Indian or Alaska Native	0 (0)	1 (2)	0 (0)	0 (0)	1 (1)	0 (0)
	Native Hawaiian or Other Pacific Islander	0 (0)	0 (0)	0 (0)	2 (2)	1 (1)	0 (0)
	Ethnicity						
	Hispanic or Latino	53 (27)	19 (35)	0 (0)	21 (18)	47 (28)	45 (30)
	Not Hispanic or Latino	101 (51)	19 (35)	0 (0)	64 (55)	94 (55)	84 (56)
	Sex						
	Female	91 (46)	14 (26)	0 (0)	32 (28)	71 (42)	54 (36)
	Male	74 (38)	32 (59)	0 (0)	57 (49)	73 (43)	79 (52)
	Unknown concept	32 (16)	8 (15)	0 (0)	27 (23)	26 (15)	18 (12)
	DCSI (SD)&	1.2 (1.3)	1.22 (1.25)	0 (0)	1.71 (1.48)	2.04 (1.53)	1.7 (1.75)
	CCI (SD)&	2.22 (2.44)	2.41 (2.56)	0 (0)	2.69 (2.42)	3.34 (2.77)	3.02 (3.12)
	Essential hypertension	4 (2)	6 (11)	0 (0)	3 (3)	54 (32)	40 (26)
	Hyperlipidemia	4 (2)	5 (9)	0 (0)	3 (3)	50 (29)	31 (21)

© 2025 Cai CX et al. JAMA Ophthalmology.

	Obstructive sleep apnea	1 (1)	1 (2)	0 (0)	2 (2)	3 (2)	7 (5)
	Chronic kidney disease	0 (0)	1 (2)	0 (0)	1 (1)	13 (8)	12 (8)
	Anemia	1 (1)	0 (0)	0 (0)	1 (1)	5 (3)	10 (7)
**VA**		**N=6852**	**N=547**	**N=25**	**N=62256**	**N=170**	**N=61819**
	Age (years)*						
	≤29	13 (0)	0 (0)	0 (0)	65 (0)	0 (0)	100 (0)
	30-49	1330 (19)	93 (13)	20 (0)	6396 (10)	20 (0)	8506 (14)
	50-69	3819 (56)	275 (50)	40 (0)	28821 (46)	78 (40)	32518 (53)
	≥70	1699 (25)	198 (33)	10 (0)	26989 (43)	106 (45)	20709 (33)
	Race						
	American Indian or Alaska Native	54 (1)	<10 (0)	0 (0)	500 (1)	0 (0)	599 (1)
	Asian	71 (1)	<10 (0)	0 (0)	970 (2)	<10 (0)	883 (1)
	Black or African American	1567 (23)	100 (18)	<10 (0)	11779 (19)	42 (25)	11957 (19)
	Native Hawaiian or Other Pacific Islander	86 (1)	<10 (0)	0 (0)	723 (1)	0 (0)	709 (1)
	White	4587 (67)	381 (70)	17 (68)	44229 (71)	117 (69)	43863 (71)
	Ethnicity						
	Hispanic or Latino	494 (7)	31 (6)	<10 (0)	4579 (7)	13 (8)	4979 (8)
	Not Hispanic or Latino	6153 (90)	496 (91)	21 (84)	55991 (90)	154 (91)	55442 (90)
	Sex						
	Female	1407 (21)	76 (14)	<10 (0)	4115 (7)	10 (6)	4394 (7)
	Male	5445 (79)	471 (86)	20 (80)	58141 (93)	160 (94)	57425 (93)
	DCSI (SD)&	2.7 (2.1)	3.1 (2.27)	2.4 (1.62)	3.22 (2.28)	3.44 (2.18)	2.84 (2.13)
	CCI (SD)&	3.96 (2.43)	4.12 (2.37)	3.32 (1.7)	4.49 (2.65)	4.52 (2.51)	3.98 (2.35)
	Essential hypertension	4882 (71)	390 (71)	12 (48)	47519 (76)	111 (65)	44693 (72)
	Hyperlipidemia	4670 (68)	368 (67)	15 (60)	46701 (75)	100 (59)	43858 (71)
	Obstructive sleep apnea	3183 (46)	185 (34)	<10 (0)	20175 (32)	37 (22)	15860 (26)
	Chronic kidney disease	486 (7)	50 (9)	<10 (0)	7021 (11)	19 (11)	4351 (7)
	Anemia	624 (9)	32 (6)	0 (0)	6428 (10)	14 (8)	4815 (8)
	Interferon use	0 (0)	0 (0)	0 (0)	<10 (0)	0 (0)	19 (0)
	Amiodarone use	43 (1)	<10 (0)	0 (0)	1387 (2)	<10 (0)	480 (1)
	Phosphodiesterase inhibitor use	0 (0)	0 (0)	0 (0)	24 (0)	0 (0)	<10 (0)

© 2025 Cai CX et al. JAMA Ophthalmology.

WashU		N=1840	N=868	N=78	N=1549	N=1098	N=1158
	Age (years)*						
	≤29	46 (2)	29 (3)	5 (0)	18 (1)	16 (1)	10 (0)
	30-49	502 (27)	215 (25)	24 (18)	235 (15)	158 (14)	154 (13)
	50-69	1076 (58)	524 (60)	45 (58)	909 (59)	592 (54)	619 (53)
	≥70	219 (12)	103 (11)	19 (12)	397 (25)	332 (30)	382 (33)
	Race						
	American Indian or Alaska Native	5 (0)	<5 (0)	0 (0)	<5 (0)	<5 (0)	<5 (0)
	Asian	22 (1)	6 (1)	<5 (0)	39 (3)	34 (3)	21 (2)
	Black or African American	476 (26)	210 (24)	12 (15)	382 (25)	260 (24)	234 (20)
	Other Pacific Islander	<5 (0)	<5 (0)	<5 (0)	<5 (0)	<5 (0)	<5 (0)
	White	1323 (72)	640 (74)	64 (82)	1110 (72)	784 (71)	889 (77)
	Ethnicity						
	Hispanic or Latino	30 (2)	12 (1)	0 (0)	26 (2)	13 (1)	13 (1)
	Not Hispanic or Latino	1800 (98)	851 (98)	78 (100)	1512 (98)	1077 (98)	1141 (99)
	Sex						
	Female	1235 (67)	584 (67)	49 (63)	646 (42)	591 (54)	565 (49)
	Male	605 (33)	284 (33)	29 (37)	903 (58)	507 (46)	593 (51)
	DCSI (SD)&	2.19 (1.73)	2.4 (1.72)	2.46 (2.04)	2.91 (1.99)	2.65 (1.92)	2.79 (1.99)
	CCI (SD)&	4.1 (2.42)	4.24 (2.38)	4.58 (2.28)	4.76 (2.55)	4.67 (2.44)	4.86 (2.44)
	Essential hypertension	1173 (64)	554 (64)	53 (68)	1023 (66)	744 (68)	726 (63)
	Hyperlipidemia	1224 (67)	582 (67)	51 (65)	1196 (77)	787 (72)	839 (72)
	Obstructive sleep apnea	537 (29)	211 (24)	14 (18)	329 (21)	200 (18)	168 (15)
	Chronic kidney disease	149 (8)	68 (8)	7 (9)	197 (13)	136 (12)	146 (13)
	Anemia	197 (11)	100 (12)	5 (6)	158 (10)	126 (11)	107 (9)
	Interferon use	<5 (0)	<5 (0)	0 (0)	0 (0)	<5 (0)	<5 (0)
	Phosphodiesterase inhibitor use	<5 (0)	0 (0)	0 (0)	<5 (0)	0 (0)	0 (0)

Abbreviations: T2DM = type 2 diabetes mellitus, GLP-1 RA = glucagon-like peptide 1 receptor agonist, SGLT2 = sodium-glucose cotransporter-2, DPP4 = dipeptidyl peptidase-4, DCSI = Diabetes Complications Severity Index, CCI = Charlson Comorbidity Index-Romano adaptation, SD = standard deviation

Database Abbreviations:
CCAE = Merative MarketScan Commercial Claims and Encounters Database

© 2025 Cai CX et al. JAMA Ophthalmology.

CUMC = Columbia University Medical Center
IQVIA = IQVIA Open Claims
JHME = Johns Hopkins Medical Enterprise
MDCD = Merative MarketScan Multi-State Medicaid Database
MDCR = Merative MarketScan Medicare Supplemental and Coordination of Benefits Database
OHSU = Oregon Health & Science University
Optum EHR = Optum de-identified Electronic Health Record data set
PharMetrics = PharMetrics Plus
STARR = Stanford University
USC = Keck Medical Center of University of Southern California
VA = Department of Veterans Affairs
WashU = Washington University in St. Louis

© 2025 Cai CX et al. JAMA Ophthalmology.

eTable 11: The incidence proportion and incidence rate of NAION among adults with T2DM and in each T2DM drug exposure cohort (semaglutide, dulaglutide, exenatide, empagliflozin, sitagliptin, glipizide) across all databases.

		"Sensitive" NAION definition[&]					"Specific" NAION definition[&]				
Cohort	Database	Patients at Risk*	On-Treatment Time (Person-Years)	Number of Outcomes	Incidence Proportion (per 100,000 Persons)	Incidence Rate (per 100,000 Person-Years)	Patients at Risk*	On-Treatment Time (Person-Years)	Number of Outcomes	Incidence Proportion (per 100,000 Persons)	Incidence Rate (per 100,000 Person-Years)
T2DM	Total[%]	37076692	75093073	26501	78.3	41	37096287	75165063	10473	32	16.8
	CCAE	2126003	3427034	1185	55.7	34.6	2126453	3428658	541	25.4	15.8
	Clinformatics	4010797	7443649	6269	156.3	84.2	4013178	7454714	2392	59.6	32.1
	CUMC	99138	213381.6	39	39.3	18.3	99183	213523.4	16	16.1	7.5
	IQVIA	20155571	43536250	9976	49.5	22.9	20167656	43576868	3892	19.3	8.9
	JHME	138751	227697.3	79	56.9	34.7	138833	227892	30	21.6	13.2
	MDCD	867613	1650953	826	95.2	50	868099	1652770	256	29.5	15.5
	MDCR	501145	824223.2	851	169.8	103.2	501599	825674.9	327	65.2	39.6
	OHSU	54958	122074.7	32	58.2	26.2	55019	122219.7	19	34.5	15.5
	Optum EHR	2516415	4303811	562	22.3	13.1	2516877	4305088	307	12.2	7.1
	PharMetrics	5619829	10736023	5348	95.2	49.8	5621567	10744401	2225	39.6	20.7
	STARR	68735	153558	31	45.1	20.2	68757	153655.9	17	24.7	11.1
	USC	41431	47863	21	50.7	43.9	41445	47886.4	10	24.1	20.9
	VA	753008	2053014	1235	164	60.2	754240	2057853	416	55.2	20.2
	WashU	123298	353541.1	47	38.1	13.3	123381	353858.1	25	20.3	7.1
Semaglutide (GLP-1 RA)	Total[%]	810390	400136.6	89	7.1	14.5	810937	400423.9	51	4.2	8.7
	CCAE	50173	26646.6	11	21.9	41.3	50194	26657.4	6	12	22.5
	Clinformatics	43555	20212.7	13	29.8	64.3	43588	20228.6	10	22.9	49.4
	CUMC	1794	1491.4	0	0	0	1796	1492.4	0	0	0
	IQVIA	581923	290882.1	52	8.9	17.9	582336	291103.7	30	5.2	10.3
	JHME	1473	1003.5	<5	NA	NA	1473	1003.6	0	0	0
	MDCD	2108	680.2	0	0	0	2111	681	0	0	0
	MDCR	3665	1710.4	0	0	0	3670	1712.4	0	0	0
	OHSU	602	384.1	0	0	0	603	384.2	0	0	0

© 2025 Cai CX et al. JAMA Ophthalmology.

	Optum EHR	38711	12501.5	<5	NA	NA	38719	12507.5	<5	NA	NA
	PharMetrics	76572	38014.4	8	10.4	21	76618	38037.7	5	6.5	13.1
	STARR	979	791.1	<5	NA	NA	979	791.1	<5	NA	NA
	USC	196	57.5	0	0	0	196	57.5	0	0	0
	VA	6824	4428.6	<10	NA	NA	6837	4432.9	<10	NA	NA
	WashU	1815	1332.7	0	0	0	1817	1333.9	0	0	0
Dulaglutide (GLP-1 RA)	Total[%]	326282	247706	54	7.9	13.4	326520	247868.5	31	3.2	4.2
	CCAE	16453	13651	<5	NA	NA	16465	13658.9	<5	NA	NA
	Clinformatics	14896	10757	<5	NA	NA	14912	10769.4	<5	NA	NA
	CUMC	312	332.3	0	0	0	313	334.8	0	0	0
	IQVIA	237140	185935.9	36	15.2	19.4	237313	186055.8	19	8	10.2
	JHME	561	733.8	0	0	0	561	733.8	0	0	0
	MDCD	6450	3555.6	0	0	0	6455	3557.6	0	0	0
	MDCR	1308	921.2	0	0	0	1311	921.7	0	0	0
	OHSU	193	205.7	0	0	0	193	205.7	0	0	0
	Optum EHR	18323	7179.8	5	27.3	69.6	18328	7181.9	<5	NA	NA
	PharMetrics	28936	22221.7	13	44.9	58.5	28955	22234.9	7	24.2	31.5
	STARR	260	393.5	0	0	0	260	393.5	0	0	0
	USC	54	28.2	0	0	0	54	28.2	0	0	0
	VA	539	518.1	<10	NA	NA	541	518.6	<10	NA	NA
	WashU	857	1272.1	0	0	0	859	1273.6	0	0	0
Exenatide (GLP-1 RA)	Total[%]	25936	14239.3	20	0	0	25951	14254.3	15	0	0
	CCAE	1037	639	<5	NA	NA	1038	639.8	0	0	0
	Clinformatics	1413	915	<5	NA	NA	1414	915.3	<5	NA	NA
	CUMC	16	15.4	0	0	0	16	15.4	0	0	0
	IQVIA	18677	10559.4	-5	NA	NA	18690	10573.2	-5	NA	NA
	JHME	19	19.3	0	0	0	19	19.3	0	0	0
	MDCD	738	350	0	0	0	738	350	0	0	0
	MDCR	68	37.7	0	0	0	68	37.7	0	0	0
	OHSU	31	27.7	0	0	0	31	27.7	0	0	0
	Optum EHR	1876	427.7	0	0	0	1876	427.7	0	0	0
	PharMetrics	1949	1087.3	<5	NA	NA	1949	1087.3	<5	NA	NA
	STARR	9	6.2	0	0	0	9	6.2	0	0	0
	USC	0	0	0	NA	NA	0	0	0	NA	NA

© 2025 Cai CX et al. JAMA Ophthalmology.

	VA	25	11.7	0	0	0	25	11.7	0	0	0
	WashU	78	142.8	0	0	0	78	142.8	0	0	0
Empagliflozin (SGLT2 inhibitor)	Total%	715802	537237.9	138	10.4	13.7	716626	537896.5	66	4	5.2
	CCAE	27558	21932.7	5	18.1	22.8	27575	21942	<5	NA	NA
	Clinformatics	43207	30197.7	8	18.5	26.5	43262	30242.5	<5	NA	NA
	CUMC	1371	1434.8	0	0	0	1373	1437.5	0	0	0
	IQVIA	480390	367667.7	74	15.4	20.1	480886	368058.5	26	5.4	7.1
	JHME	1062	1191.8	0	0	0	1063	1191.8	0	0	0
	MDCD	5644	3311.8	<5	NA	NA	5653	3320.1	0	0	0
	MDCR	5266	3323.6	<5	NA	NA	5278	3334.4	<5	NA	NA
	OHSU	566	490	0	0	0	568	490.5	0	0	0
	Optum EHR	34549	16432	<5	NA	NA	34556	16437	<5	NA	NA
	PharMetrics	51873	41427.5	13	25.1	31.4	51898	41455.8	7	13.5	16.9
	STARR	751	1060.8	0	0	0	751	1060.8	0	0	0
	USC	116	50.5	0	0	0	116	50.5	0	0	0
	VA	61916	46495.6	23	37.1	49.5	62112	46650.1	13	20.9	27.9
	WashU	1533	2221.4	0	0	0	1535	2225.1	0	0	0
Sitagliptin (DPP4 inhibitor)	Total%	493563	410713.1	122	12.3	15.1	494043	411151.8	63	4.8	5.9
	CCAE	22436	18315.4	<5	NA	NA	22445	18324.8	<5	NA	NA
	Clinformatics	19534	15660.6	13	66.6	83	19571	15699.4	5	25.5	31.8
	CUMC	899	976.7	0	0	0	899	976.7	0	0	0
	IQVIA	365028	313771.2	75	20.5	23.9	365398	314118.3	36	9.9	11.5
	JHME	868	1164	0	0	0	868	1164	0	0	0
	MDCD	5087	3704.2	<5	NA	NA	5090	3704.5	<5	NA	NA
	MDCR	3205	2228.4	<5	NA	NA	3217	2234.5	0	0	0
	OHSU	139	138.6	0	0	0	139	138.6	0	0	0
	Optum EHR	35357	19918.2	<5	NA	NA	35370	19929.5	<5	NA	NA
	PharMetrics	39103	32057.1	14	35.8	43.7	39136	32079.3	7	17.9	21.8
	STARR	492	794.1	0	0	0	492	794.1	0	0	0
	USC	163	180.2	0	0	0	163	180.2	0	0	0
	VA	166	58.9	0	0	0	166	58.9	0	0	0
	WashU	1086	1745.4	0	0	0	1089	1749	0	0	0

© 2025 Cai CX et al. JAMA Ophthalmology.

Glipizide (sulfonylurea)	Total%	832295	712353.9	228	18	21.2	833131	713274.4	113	8.7	10.4
	CCAE	25424	19000.3	<5	NA	NA	25434	19015.2	<5	NA	NA
	Clinformatics	44025	38638.4	25	56.8	64.7	44067	38688.9	18	40.8	46.5
	CUMC	457	458	0	0	0	457	458	0	0	0
	IQVIA	588554	525637.1	130	22.1	24.7	589109	526260.6	54	9.2	10.3
	JHME	964	1529	0	0	0	964	1529	0	0	0
	MDCD	8197	5385.5	<5	NA	NA	8209	5392	<5	NA	NA
	MDCR	3263	2361.5	<5	NA	NA	3267	2368.5	0	0	0
	OHSU	646	824.1	0	0	0	647	824.9	0	0	0
	Optum EHR	45728	23027.5	<5	NA	NA	45746	23041.9	<5	NA	NA
	PharMetrics	51418	39009.7	15	29.2	38.5	51451	39037.4	12	23.3	30.7
	STARR	725	1322	0	0	0	725	1322	0	0	0
	USC	144	153.2	0	0	0	145	153.6	0	0	0
	VA	61600	52599.4	33	53.6	62.7	61759	52770.8	14	22.7	26.5
	WashU	1150	2408	<5	NA	NA	1151	2411.7	0	0	0

Abbreviations: NAION = nonarteritic anterior ischemic optic neuropathy, T2DM = type 2 diabetes mellitus

Database Abbreviations:
CCAE = Merative MarketScan Commercial Claims and Encounters Database
Clinformatics = Optum's de-identified Clinformatics Data Mart Database
CUMC = Columbia University Medical Center
IQVIA = IQVIA Open Claims
JHME = Johns Hopkins Medical Enterprise
MDCD = Merative MarketScan Multi-State Medicaid Database
MDCR = Merative MarketScan Medicare Supplemental and Coordination of Benefits Database
OHSU = Oregon Health & Science University
Optum EHR = Optum de-identified Electronic Health Record data set
PharMetrics = PharMetrics Plus
STARR = Stanford University
USC = Keck Medical Center of University of Southern California
VA = Department of Veterans Affairs
WashU = Washington University in St. Louis

* Patients who had NAION prior to the medication exposure or <1 day time at risk were excluded from the calculations of incidence proportion and incidence rates
& The "sensitive" NAION definition required 1 diagnosis of ischemic optic neuropathy and the "specific" NAION definition required 2 diagnoses. All other criteria were the same between the two definitions.
$ Due to data privacy issues, counts less than 5 or 10 are masked as <5 or <10 (depending on local data security policies). Incidence proportions and incidence rates cannot be calculated for these values.
% Aggregated incidence proportion and incidence rates exclude empty or NA values

© 2025 Cai CX et al. JAMA Ophthalmology.

eFigure 1: Kaplan-Meier plot showing survival as a function of time comparing new-users of semaglutide and dulaglutide using the “sensitive” NAION definition. The plot is adjusted using the propensity score. The target curve (*New user of semaglutide as 2nd-line treatment with prior T2DM and prior metformin*) shows the actual observed survival. The comparator curve (*New user of dulaglutide as 2nd-line treatment with prior T2DM and prior metformin*) applies reweighting to approximate the counterfactual of what the target survival would look like had the target cohort been exposed to the comparator instead. The shaded area denotes the 95 percent confidence interval.

CCAE

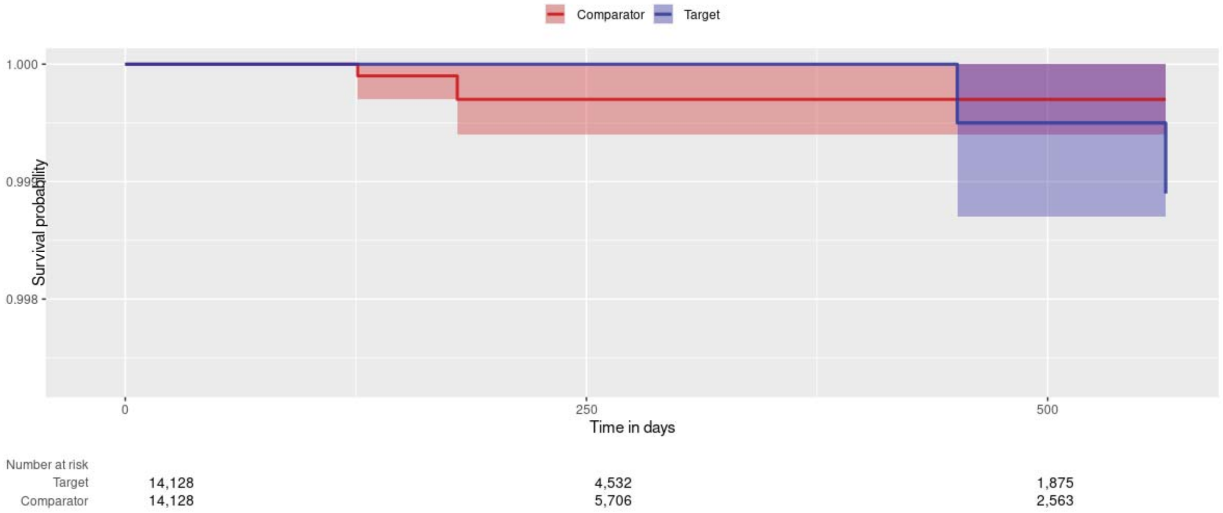

Clinformatics

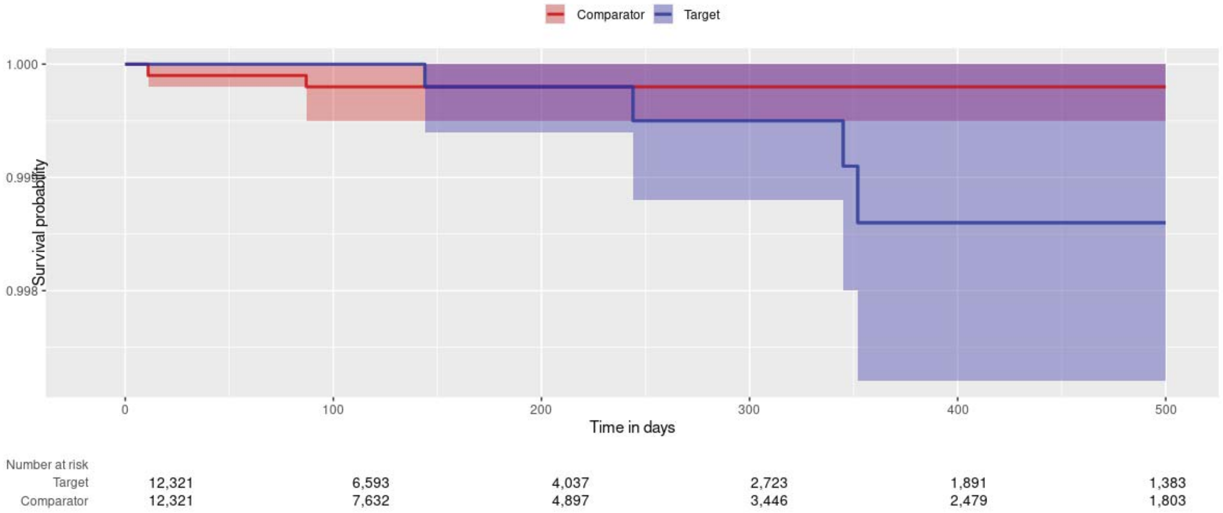

IQVIA

© 2025 Cai CX et al. JAMA Ophthalmology.

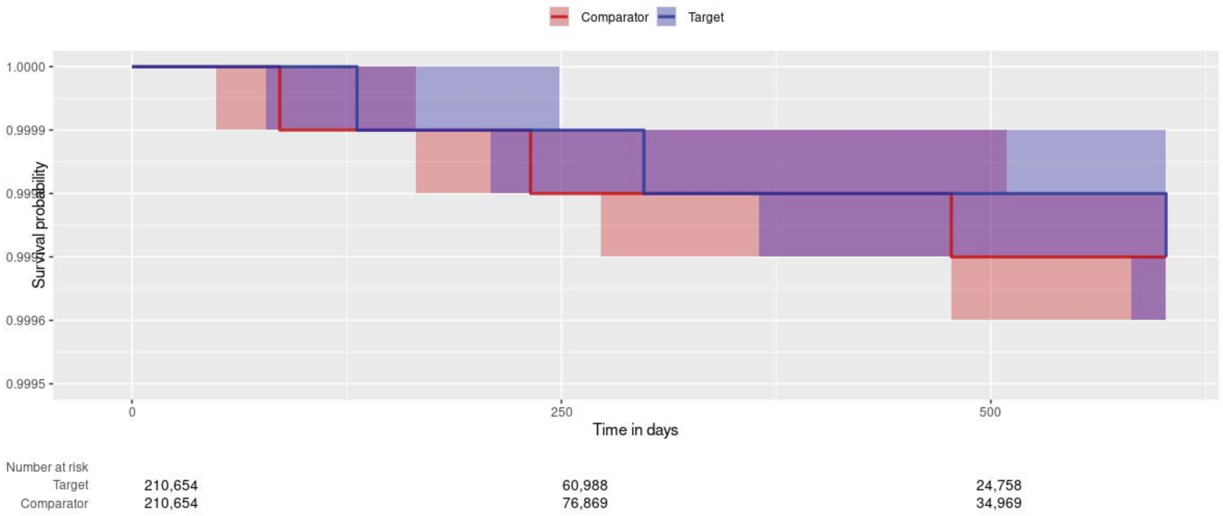

PharMetrics

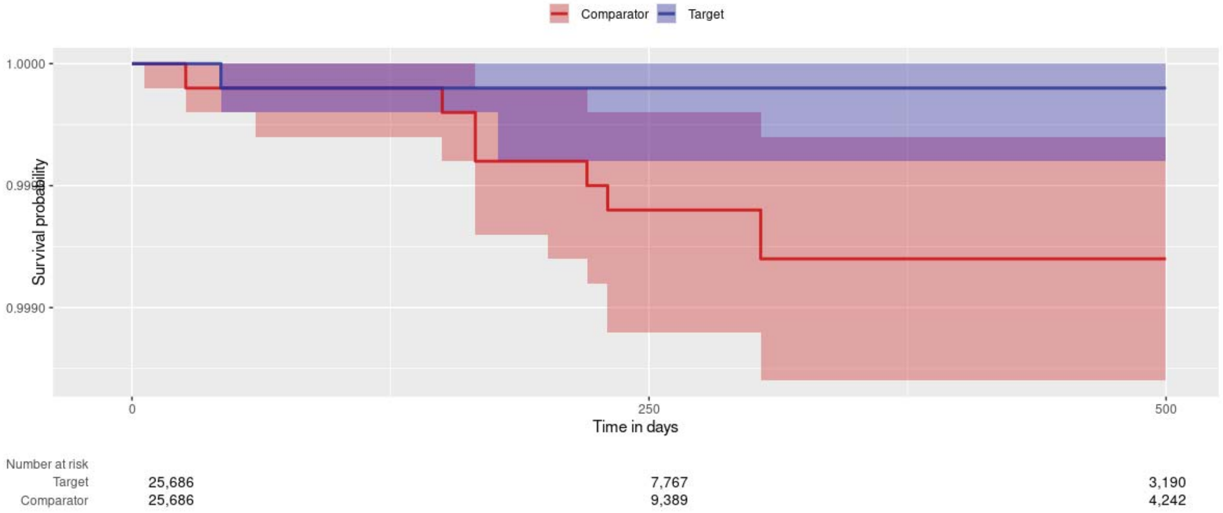

Abbreviations: T2DM = type 2 diabetes mellitus, NAION = non-arteritic anterior ischemic optic neuropathy

Database Abbreviations:
CCAE = Merative MarketScan Commercial Claims and Encounters Database
Clinformatics = Optum’s de-identified Clinformatics Data Mart Database
IQVIA = IQVIA Open Claims
PharMetrics = PharMetrics Plus

© 2025 Cai CX et al. JAMA Ophthalmology.

eFigure 2: Kaplan-Meier plot showing survival as a function of time comparing new-users of semaglutide and empagliflozin using the “sensitive” NAION definition. The plot is adjusted using the propensity score. The target curve (*New user of semaglutide as 2nd-line treatment with prior T2DM and prior metformin*) shows the actual observed survival. The comparator curve (*New user of empagliflozin as 2nd-line treatment with prior T2DM and prior metformin*) applies reweighting to approximate the counterfactual of what the target survival would look like had the target cohort been exposed to the comparator instead. The shaded area denotes the 95 percent confidence interval.

CCAE

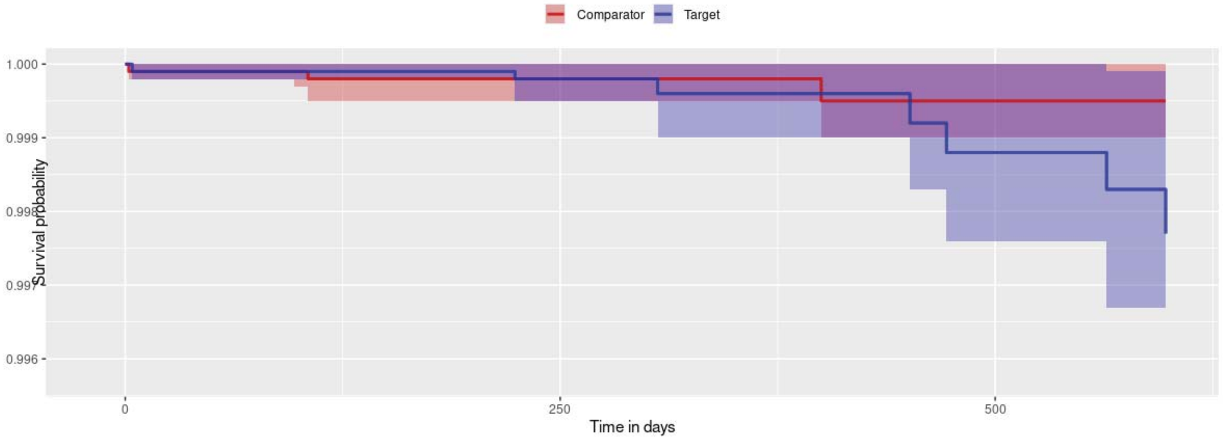

Number at risk			
Target	18,586	5,635	2,301
Comparator	18,586	6,725	2,979

Clinformatics

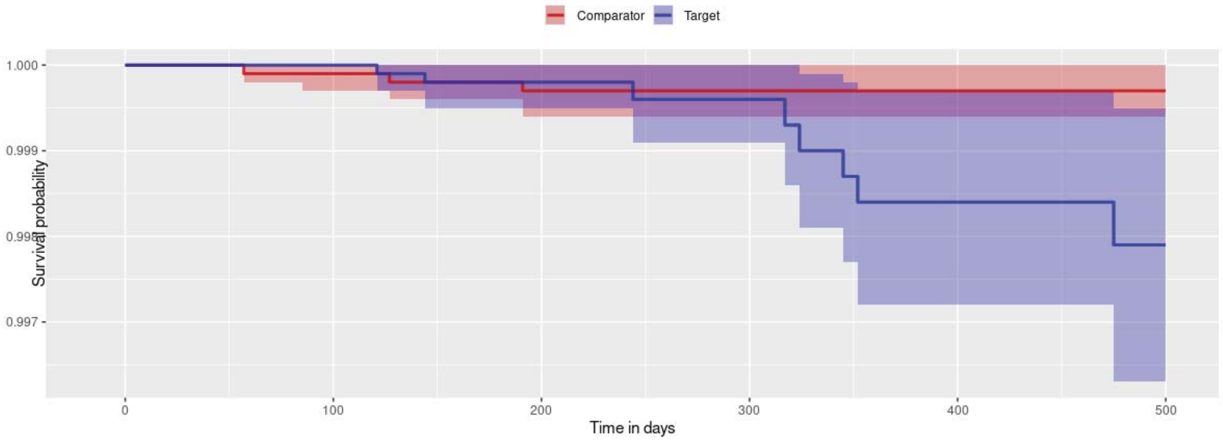

Number at risk						
Target	22,214	11,416	6,410	3,954	2,630	1,839
Comparator	22,214	13,017	8,429	5,463	3,738	2,638

IQVIA

© 2025 Cai CX et al. JAMA Ophthalmology.

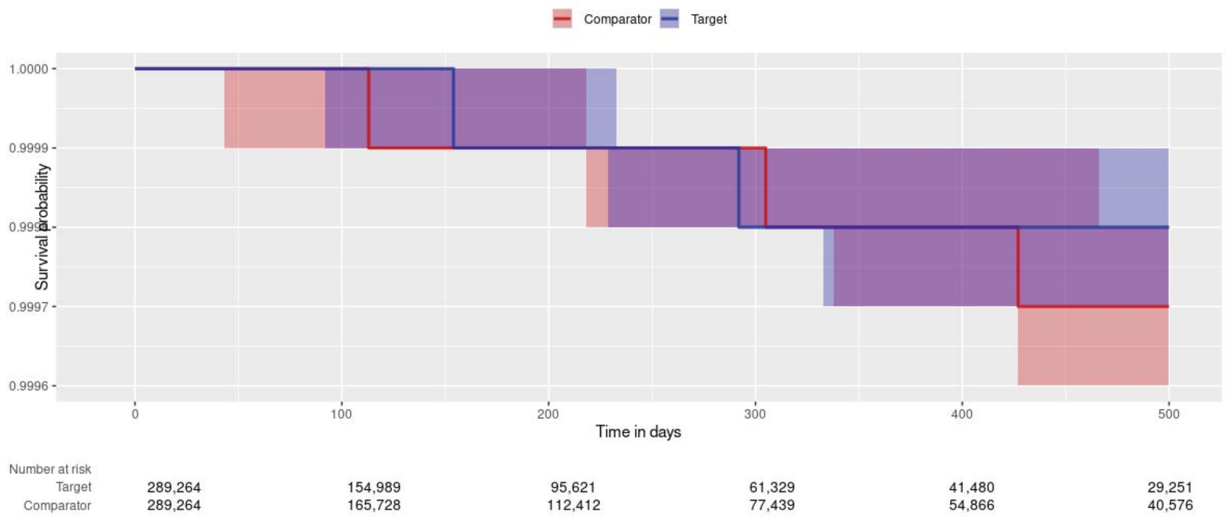

PharMetrics

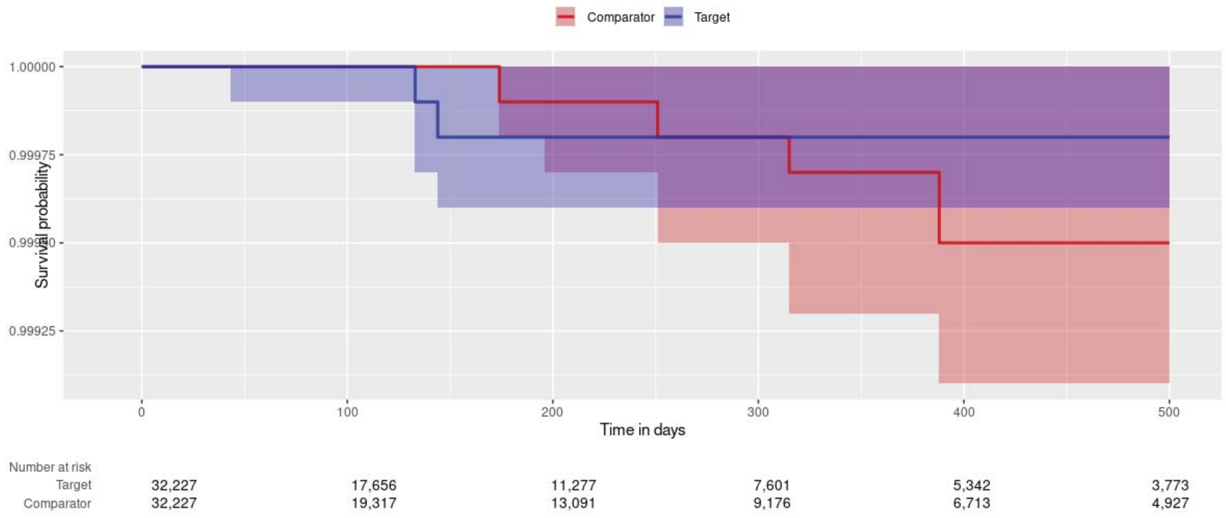

Abbreviations: T2DM = type 2 diabetes mellitus, NAION = non-arteritic anterior ischemic optic neuropathy

Database Abbreviations:
CCAE = Merative MarketScan Commercial Claims and Encounters Database
Clinformatics = Optum's de-identified Clinformatics Data Mart Database
IQVIA = IQVIA Open Claims
PharMetrics = PharMetrics Plus

© 2025 Cai CX et al. JAMA Ophthalmology.

eFigure 3: Forest plot for active-comparator cohort analysis, results from the first sensitivity analysis (not requiring second-line treatment). Hazard ratio (HR) and 95% confidence interval (CI) estimates for the risk of NAION while on-treatment with diabetes medication comparing between semaglutide, dulaglutide, exenatide, empagliflozin, sitagliptin, and glipizide. Only results from databases and comparisons that passed study diagnostics are provided, as well as the meta-analytic estimates.* Panel A on the left shows the results when applying the "sensitive" NAION definition, while Panel B on the right uses the "specific" NAION definition.

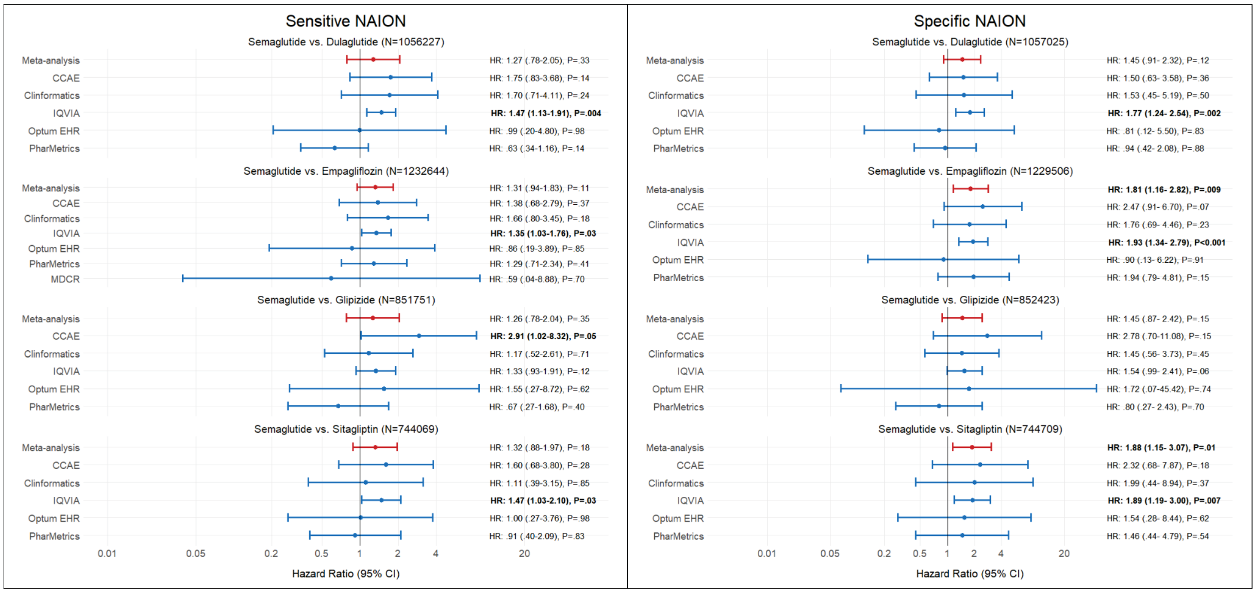

Abbreviations: NAION = non-arteritic anterior ischemic optic neuropathy

Database Abbreviations:
CCAE = Merative MarketScan Commercial Claims and Encounters Database
Clinformatics = Optum's de-identified Clinformatics Data Mart Database
IQVIA = IQVIA Open Claims
Optum EHR = Optum de-identified Electronic Health Record data set
PharMetrics = PharMetrics Plus

* Databases that have 0 cases of the outcome are unable to produce hazard ratio (HR) estimates but still contribute to the meta-analytic estimate.

© 2025 Cai CX et al. JAMA Ophthalmology.

eFigure 4: Forest plot for active-comparator cohort analysis, results from the second sensitivity analysis (restriction by calendar time). Hazard ratio (HR) and 95% confidence interval (CI) estimates for the risk of NAION while on-treatment with 2nd line diabetes medication comparing between semaglutide, dulaglutide, exenatide, empagliflozin, sitagliptin, and glipizide restricted by calendar time. Only results from databases and comparisons that passed study diagnostics are provided, as well as the meta-analytic estimates. Panel A on the left shows the results when applying the “sensitive” NAION definition, while Panel B on the right uses the “specific” NAION definition.

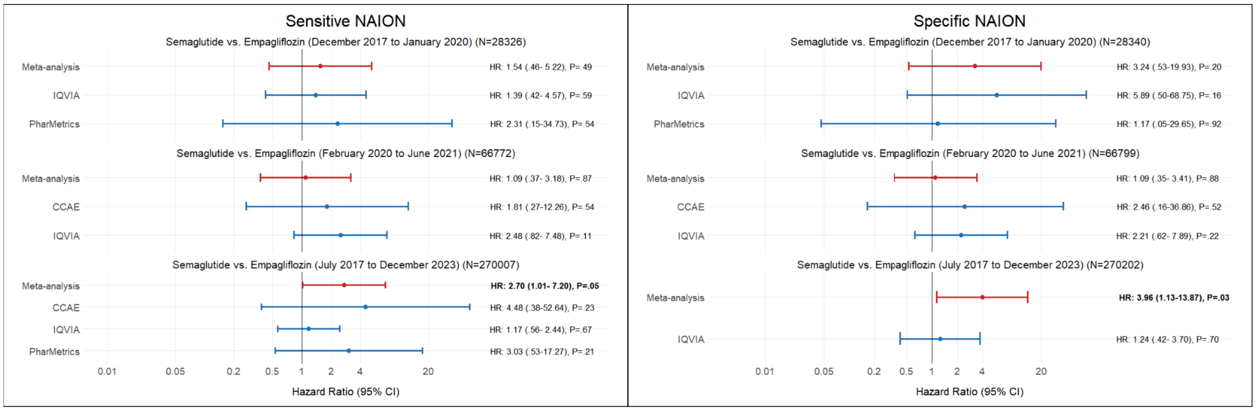

Abbreviations: NAION = non-arteritic anterior ischemic optic neuropathy

Database Abbreviations:
CCAE = Merative MarketScan Commercial Claims and Encounters Database
IQVIA = IQVIA Open Claims
PharMetrics = PharMetrics Plus

* Databases that have 0 cases of the outcome are unable to produce hazard ratio (HR) estimates but still contribute to the meta-analytic estimate.

© 2025 Cai CX et al. JAMA Ophthalmology.
