# Supplementary material for: Semaglutide and Nonarteritic Anterior Ischemic Optic Neuropathy
Source: JAMA Ophthalmol. 2025 Feb 20;143(4):304–14. doi: 10.1001/jamaophthalmol.2024.6555 (PMC11843465; doi:10.1001/jamaophthalmol.2024.6555)
Supplement: Supplement 3. — Data Sharing Statement. [file jamaophthalmol-e246555-s003.pdf]

Data Sharing Statement

Cai. Semaglutide and Nonarteritic Anterior Ischemic Optic Neuropathy. *JAMA Ophthalmol*. Published February 20, 2025. doi:10.1001/jamaophthalmol.2024.6555

Data
Data available: No
