# Supplementary material for: Semaglutide and Nonarteritic Anterior Ischemic Optic Neuropathy
Source: JAMA Ophthalmol. 2025 Feb 20;143(4):304–14. doi: 10.1001/jamaophthalmol.2024.6555 (PMC11843465; doi:10.1001/jamaophthalmol.2024.6555)
Supplement: Journal Club Slides [file jamaophthalmol-e246555-slides.pptx]

## Slide 1
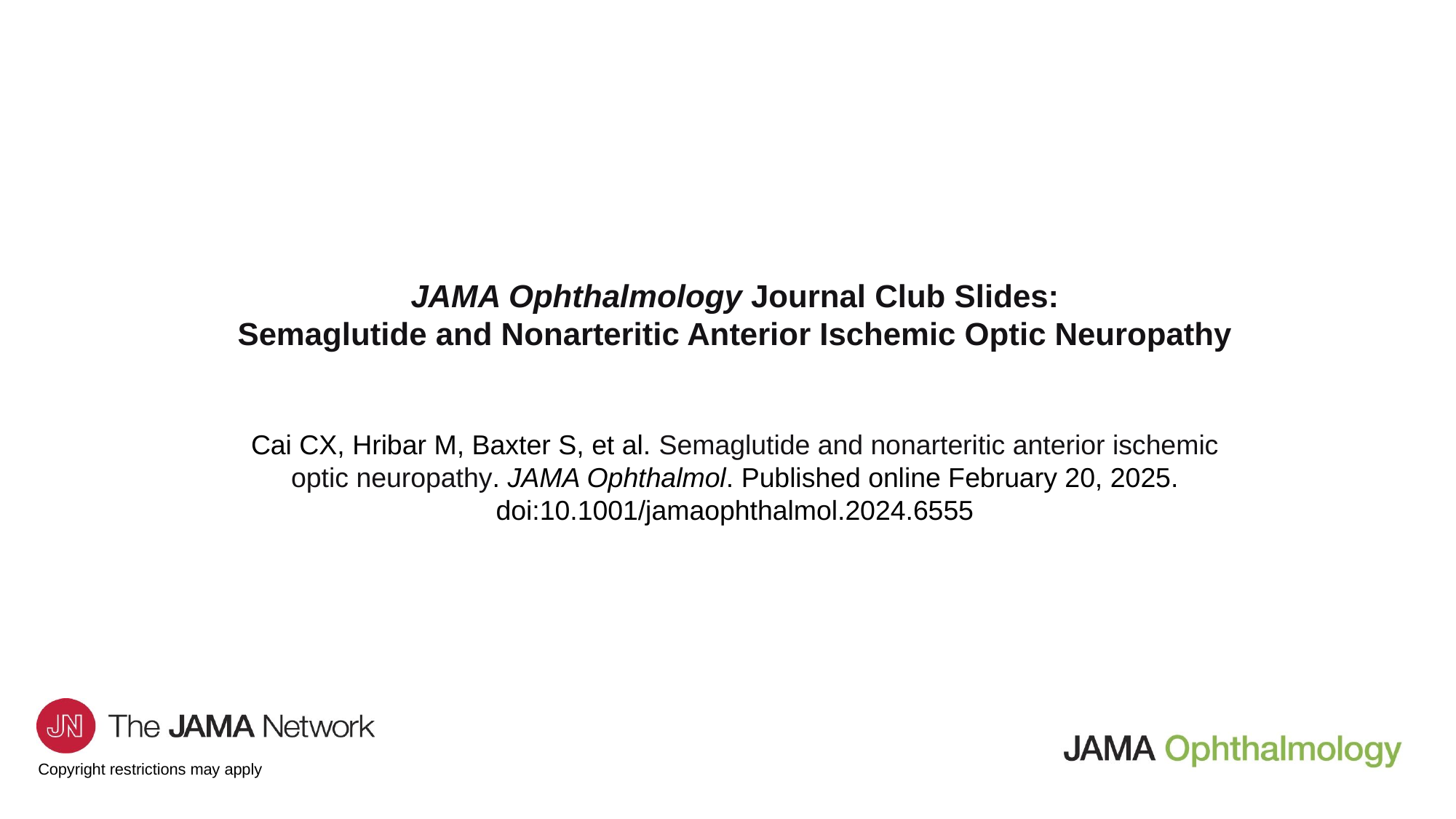

JAMA Ophthalmology Journal Club Slides:
Semaglutide and Nonarteritic Anterior Ischemic Optic Neuropathy
Cai CX, Hribar M, Baxter S, et al. Semaglutide and nonarteritic anterior ischemic optic neuropathy. JAMA Ophthalmol. Published online February 20, 2025. doi:10.1001/jamaophthalmol.2024.6555

## Slide 2
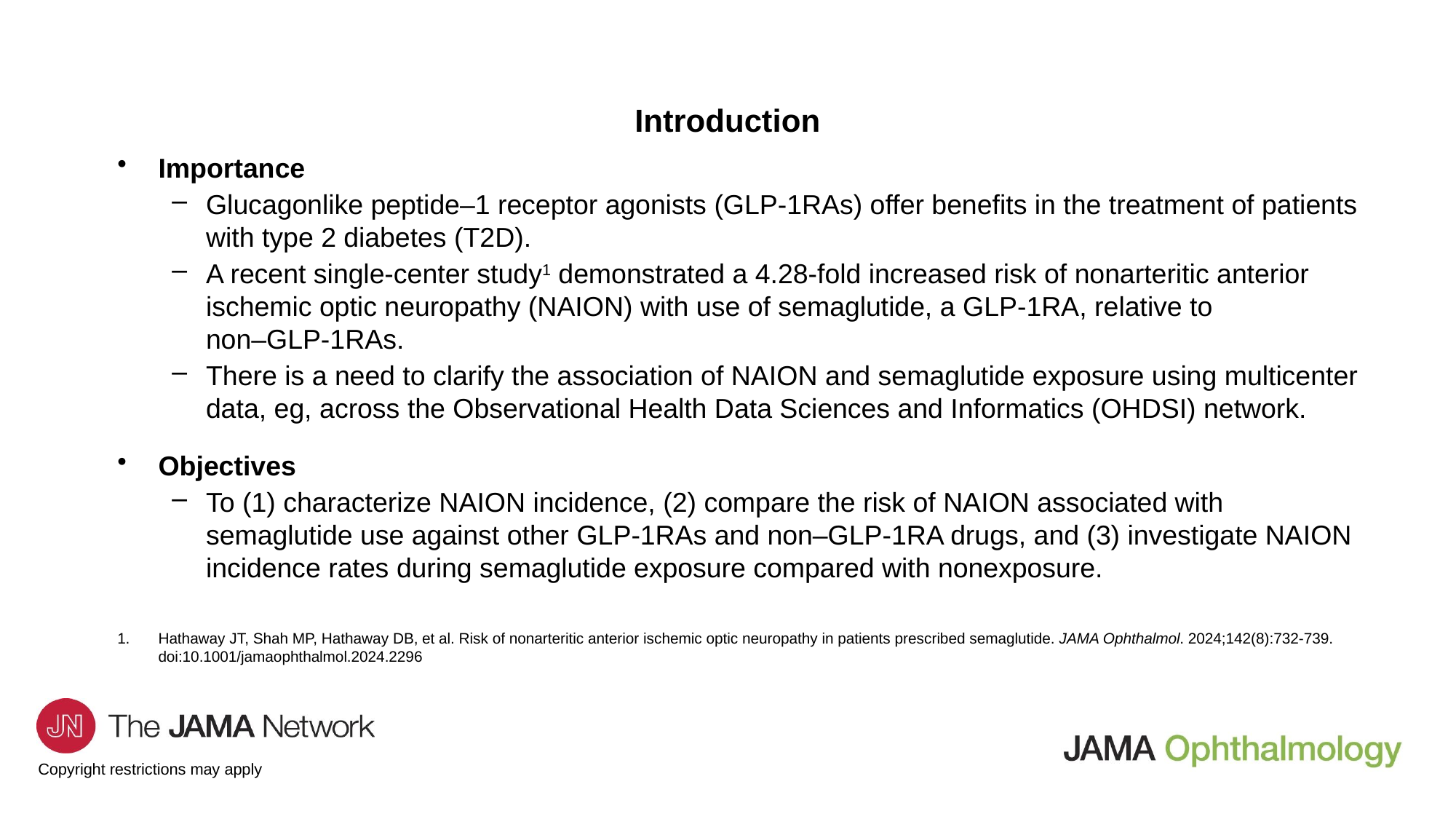

# Introduction
Importance
Glucagonlike peptide–1 receptor agonists (GLP-1RAs) offer benefits in the treatment of patients with type 2 diabetes (T2D).
A recent single-center study1 demonstrated a 4.28-fold increased risk of nonarteritic anterior ischemic optic neuropathy (NAION) with use of semaglutide, a GLP-1RA, relative to
non–GLP-1RAs.
There is a need to clarify the association of NAION and semaglutide exposure using multicenter data, eg, across the Observational Health Data Sciences and Informatics (OHDSI) network.
Objectives
To (1) characterize NAION incidence, (2) compare the risk of NAION associated with semaglutide use against other GLP-1RAs and non–GLP-1RA drugs, and (3) investigate NAION incidence rates during semaglutide exposure compared with nonexposure.
Hathaway JT, Shah MP, Hathaway DB, et al. Risk of nonarteritic anterior ischemic optic neuropathy in patients prescribed semaglutide. JAMA Ophthalmol. 2024;142(8):732-739. doi:10.1001/jamaophthalmol.2024.2296

## Slide 3
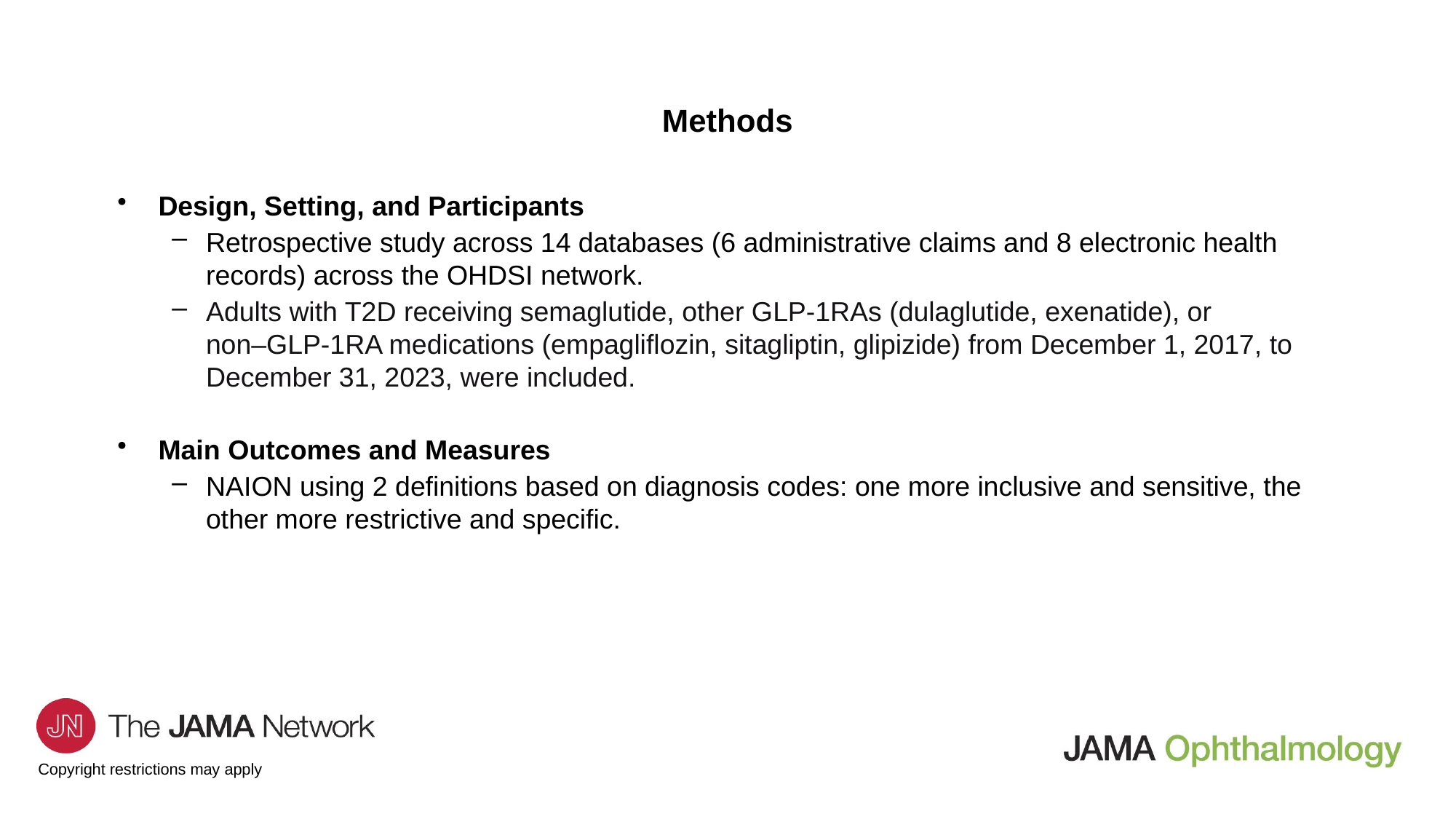

# Methods
Design, Setting, and Participants
Retrospective study across 14 databases (6 administrative claims and 8 electronic health records) across the OHDSI network.
Adults with T2D receiving semaglutide, other GLP-1RAs (dulaglutide, exenatide), or
non–GLP-1RA medications (empagliflozin, sitagliptin, glipizide) from December 1, 2017, to December 31, 2023, were included.
Main Outcomes and Measures
NAION using 2 definitions based on diagnosis codes: one more inclusive and sensitive, the other more restrictive and specific.

## Slide 4
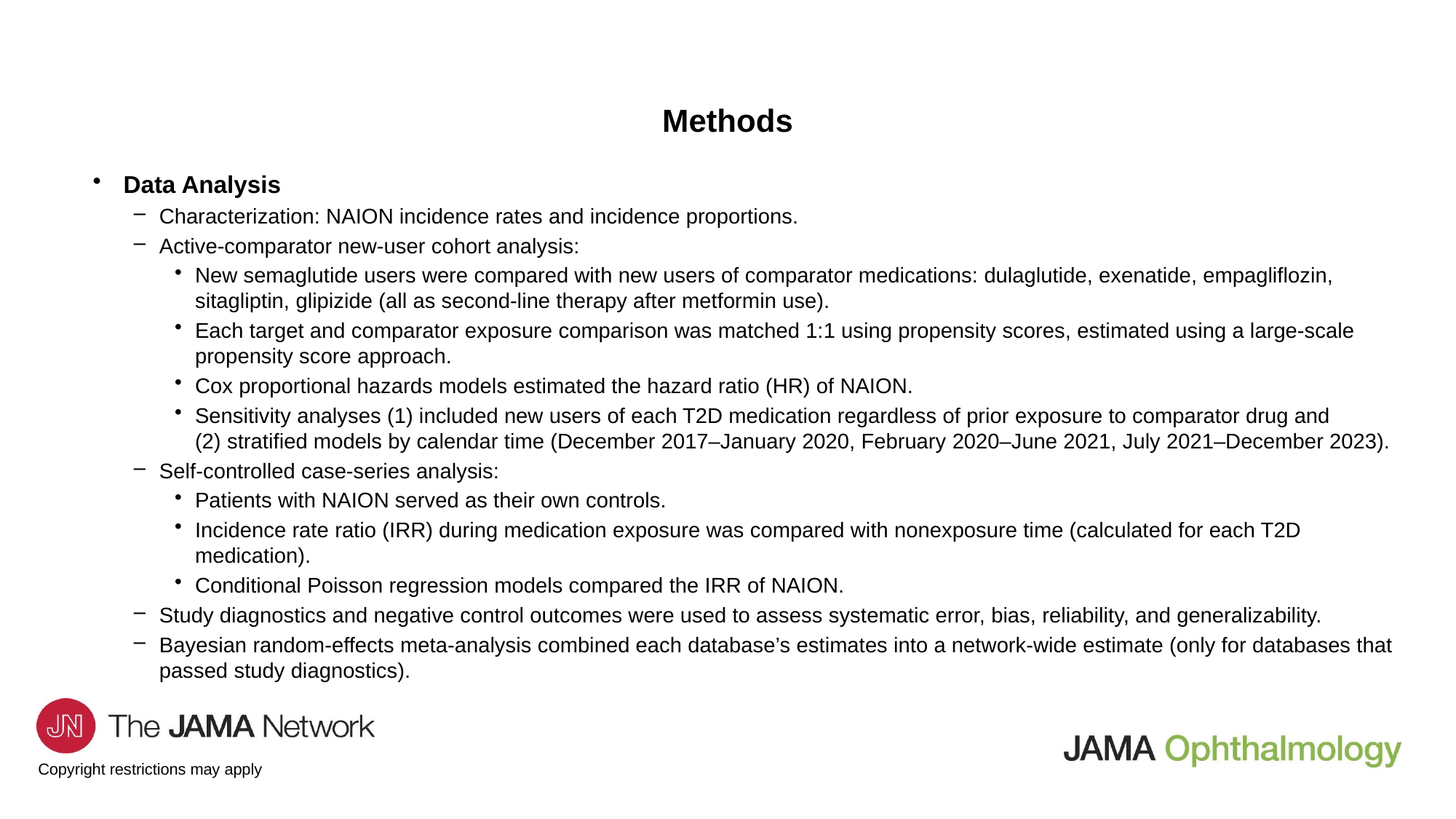

Methods
Data Analysis
Characterization: NAION incidence rates and incidence proportions.
Active-comparator new-user cohort analysis:
New semaglutide users were compared with new users of comparator medications: dulaglutide, exenatide, empagliflozin, sitagliptin, glipizide (all as second-line therapy after metformin use).
Each target and comparator exposure comparison was matched 1:1 using propensity scores, estimated using a large-scale propensity score approach.
Cox proportional hazards models estimated the hazard ratio (HR) of NAION.
Sensitivity analyses (1) included new users of each T2D medication regardless of prior exposure to comparator drug and
(2) stratified models by calendar time (December 2017–January 2020, February 2020–June 2021, July 2021–December 2023).
Self-controlled case-series analysis:
Patients with NAION served as their own controls.
Incidence rate ratio (IRR) during medication exposure was compared with nonexposure time (calculated for each T2D medication).
Conditional Poisson regression models compared the IRR of NAION.
Study diagnostics and negative control outcomes were used to assess systematic error, bias, reliability, and generalizability.
Bayesian random-effects meta-analysis combined each database’s estimates into a network-wide estimate (only for databases that passed study diagnostics).

## Slide 5
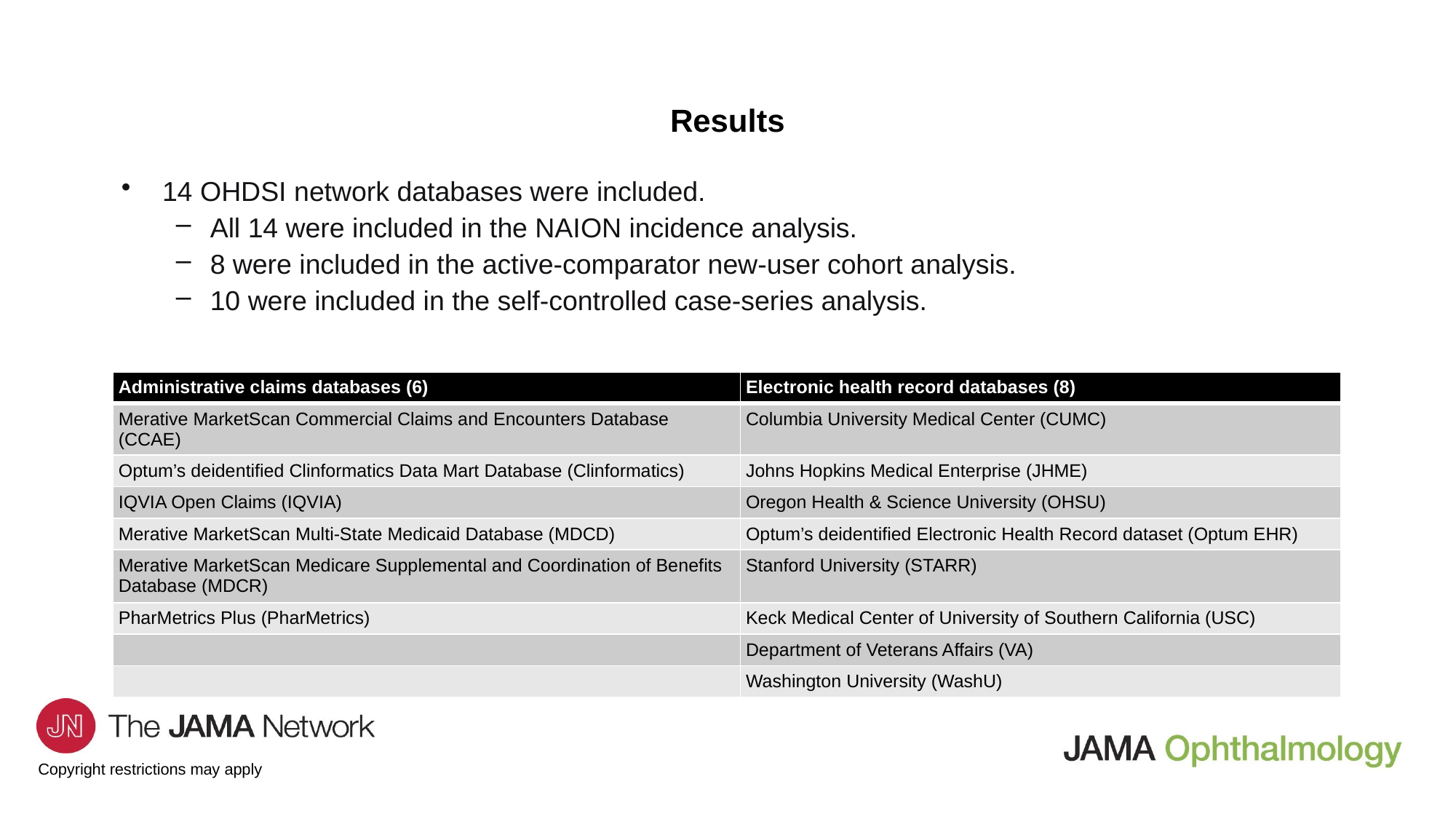

Results
14 OHDSI network databases were included.
All 14 were included in the NAION incidence analysis.
8 were included in the active-comparator new-user cohort analysis.
10 were included in the self-controlled case-series analysis.
| Administrative claims databases (6) | Electronic health record databases (8) |
| --- | --- |
| Merative MarketScan Commercial Claims and Encounters Database (CCAE) | Columbia University Medical Center (CUMC) |
| Optum’s deidentified Clinformatics Data Mart Database (Clinformatics) | Johns Hopkins Medical Enterprise (JHME) |
| IQVIA Open Claims (IQVIA) | Oregon Health & Science University (OHSU) |
| Merative MarketScan Multi-State Medicaid Database (MDCD) | Optum’s deidentified Electronic Health Record dataset (Optum EHR) |
| Merative MarketScan Medicare Supplemental and Coordination of Benefits Database (MDCR) | Stanford University (STARR) |
| PharMetrics Plus (PharMetrics) | Keck Medical Center of University of Southern California (USC) |
| | Department of Veterans Affairs (VA) |
| | Washington University (WashU) |

## Slide 6
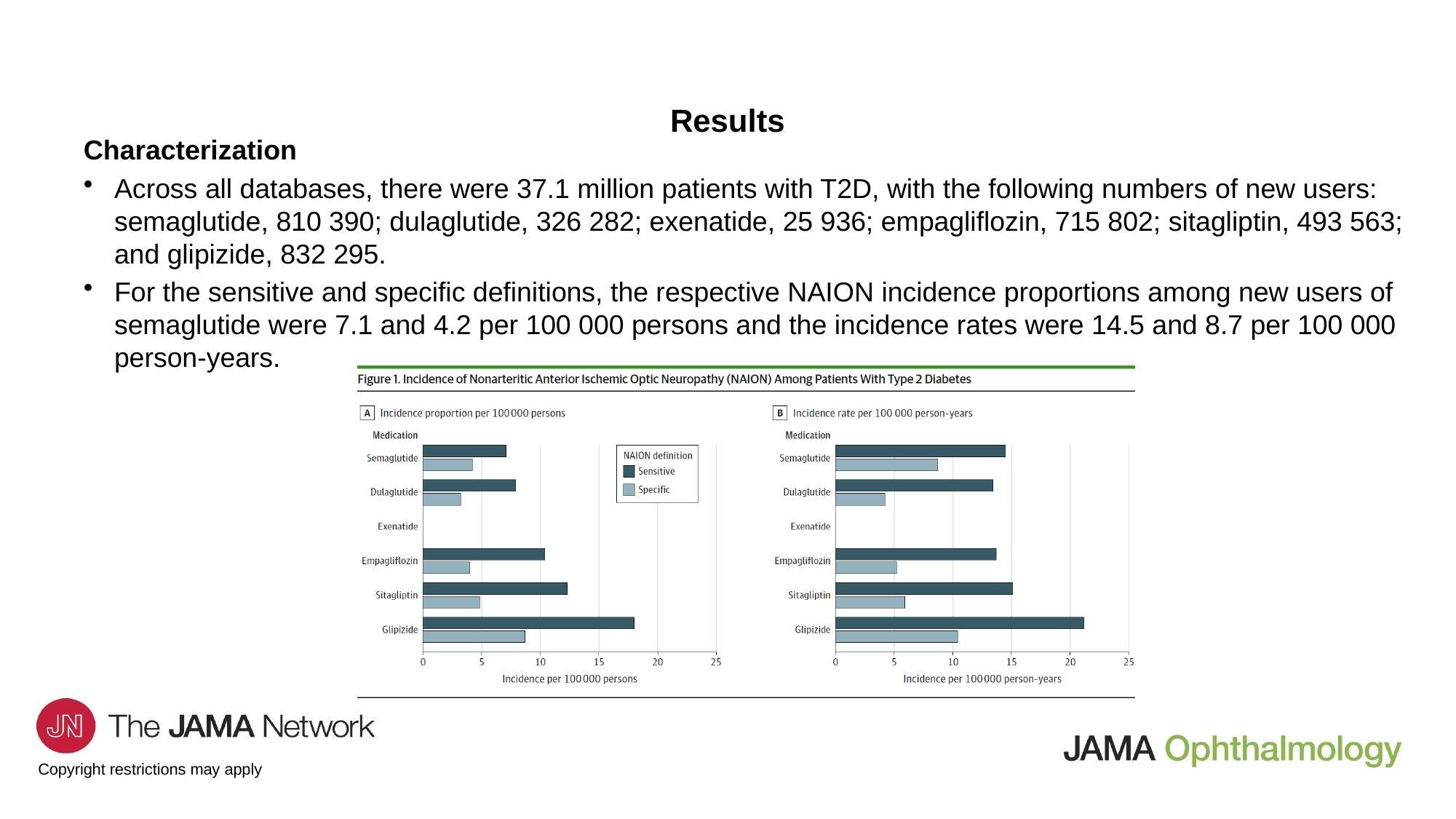

Results
Characterization
Across all databases, there were 37.1 million patients with T2D, with the following numbers of new users: semaglutide, 810 390; dulaglutide, 326 282; exenatide, 25 936; empagliflozin, 715 802; sitagliptin, 493 563; and glipizide, 832 295.
For the sensitive and specific definitions, the respective NAION incidence proportions among new users of semaglutide were 7.1 and 4.2 per 100 000 persons and the incidence rates were 14.5 and 8.7 per 100 000 person-years.

## Slide 7
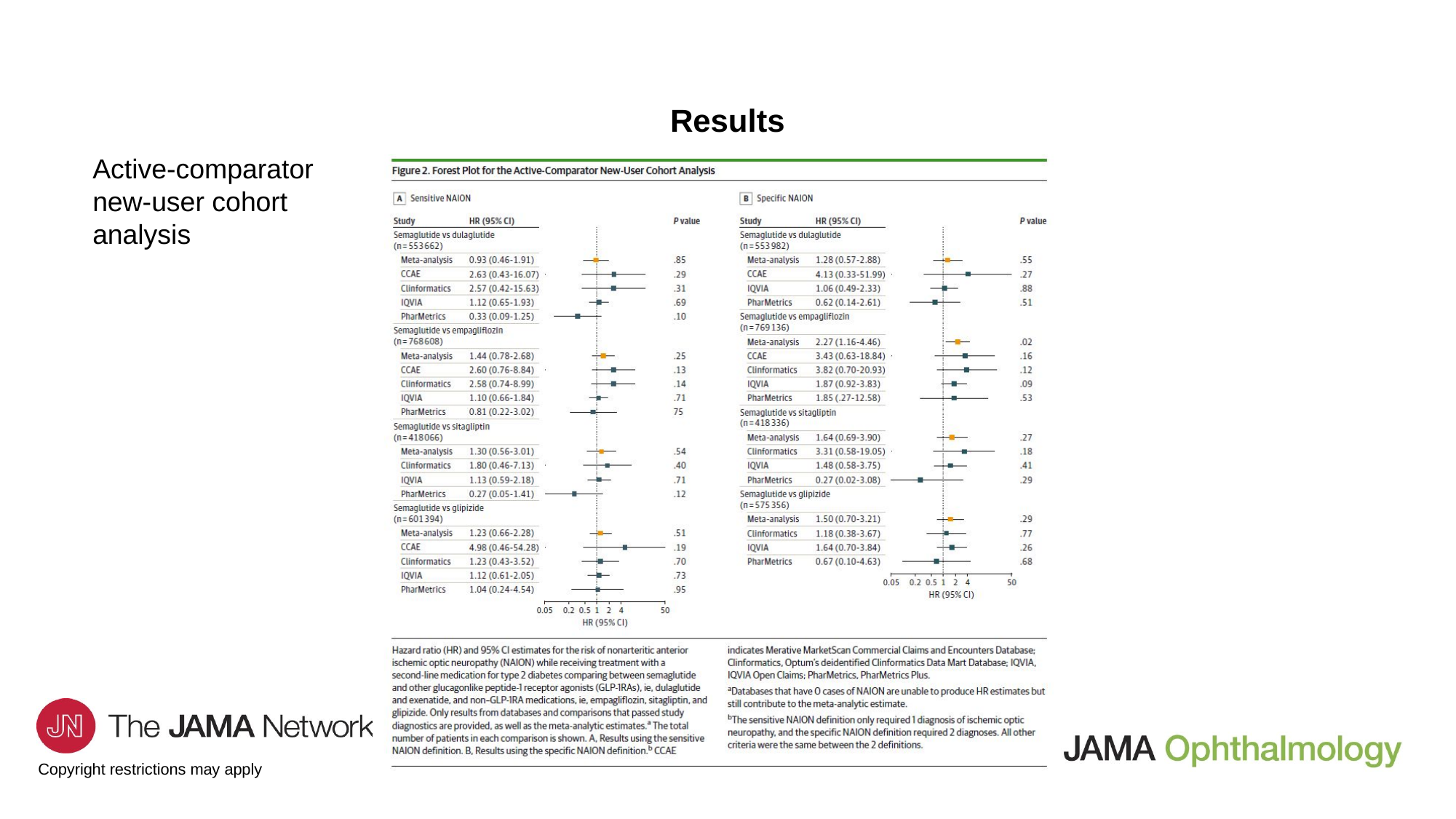

Results
Active-comparator new-user cohort analysis

## Slide 8
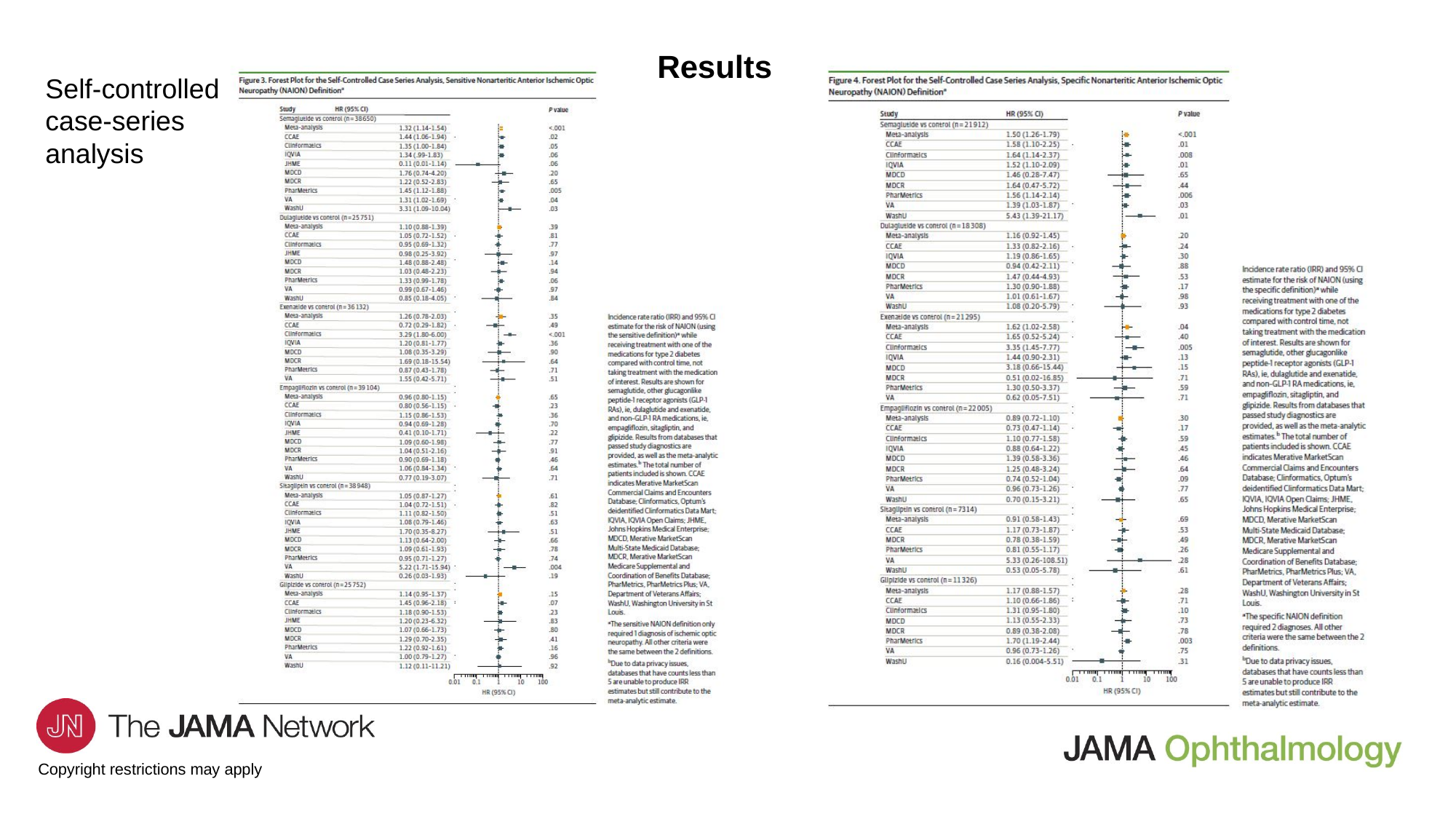

Results
Self-controlled case-series analysis

## Slide 9
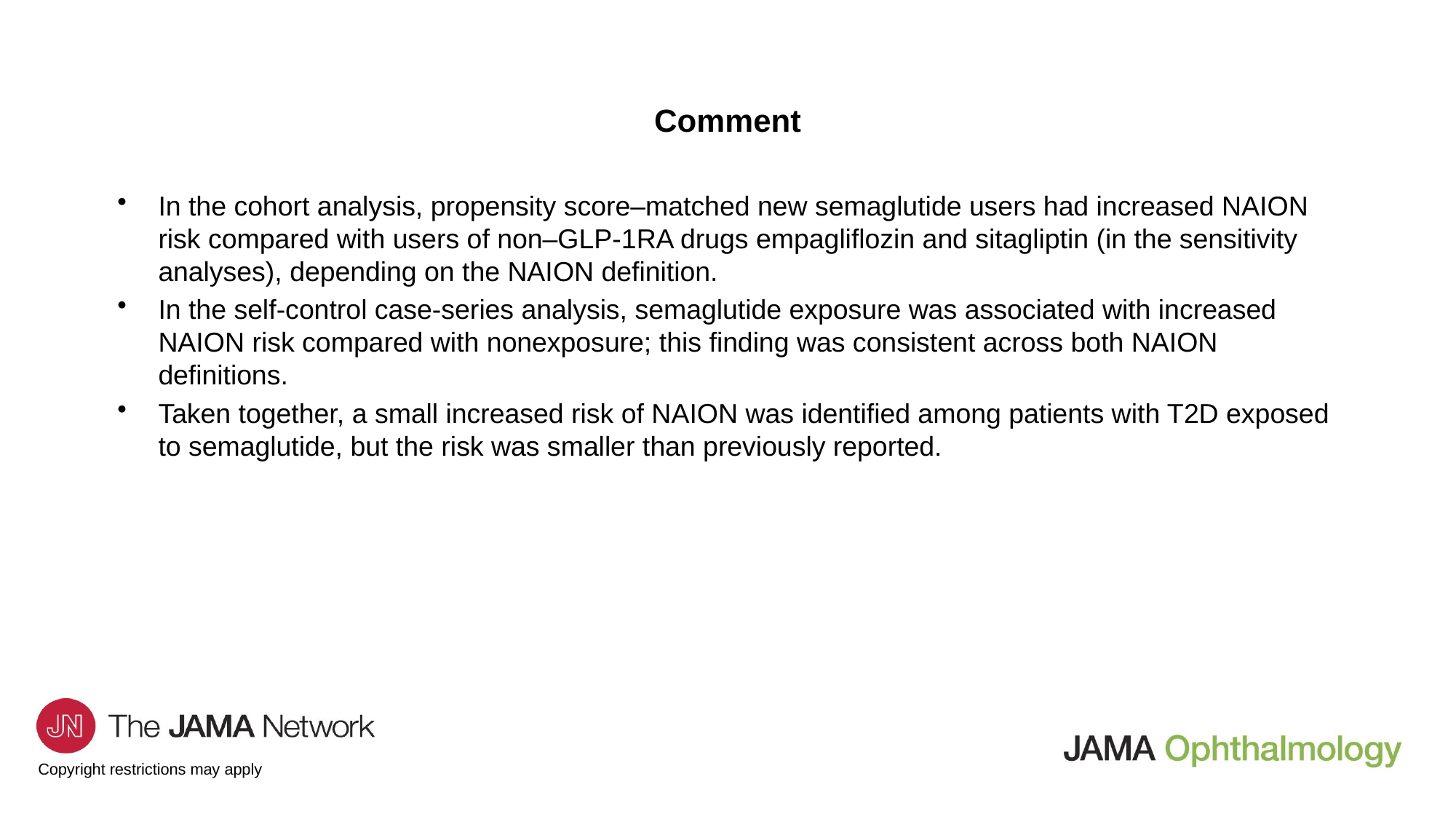

# Comment
In the cohort analysis, propensity score–matched new semaglutide users had increased NAION risk compared with users of non–GLP-1RA drugs empagliflozin and sitagliptin (in the sensitivity analyses), depending on the NAION definition.
In the self-control case-series analysis, semaglutide exposure was associated with increased NAION risk compared with nonexposure; this finding was consistent across both NAION definitions.
Taken together, a small increased risk of NAION was identified among patients with T2D exposed to semaglutide, but the risk was smaller than previously reported.

## Slide 10
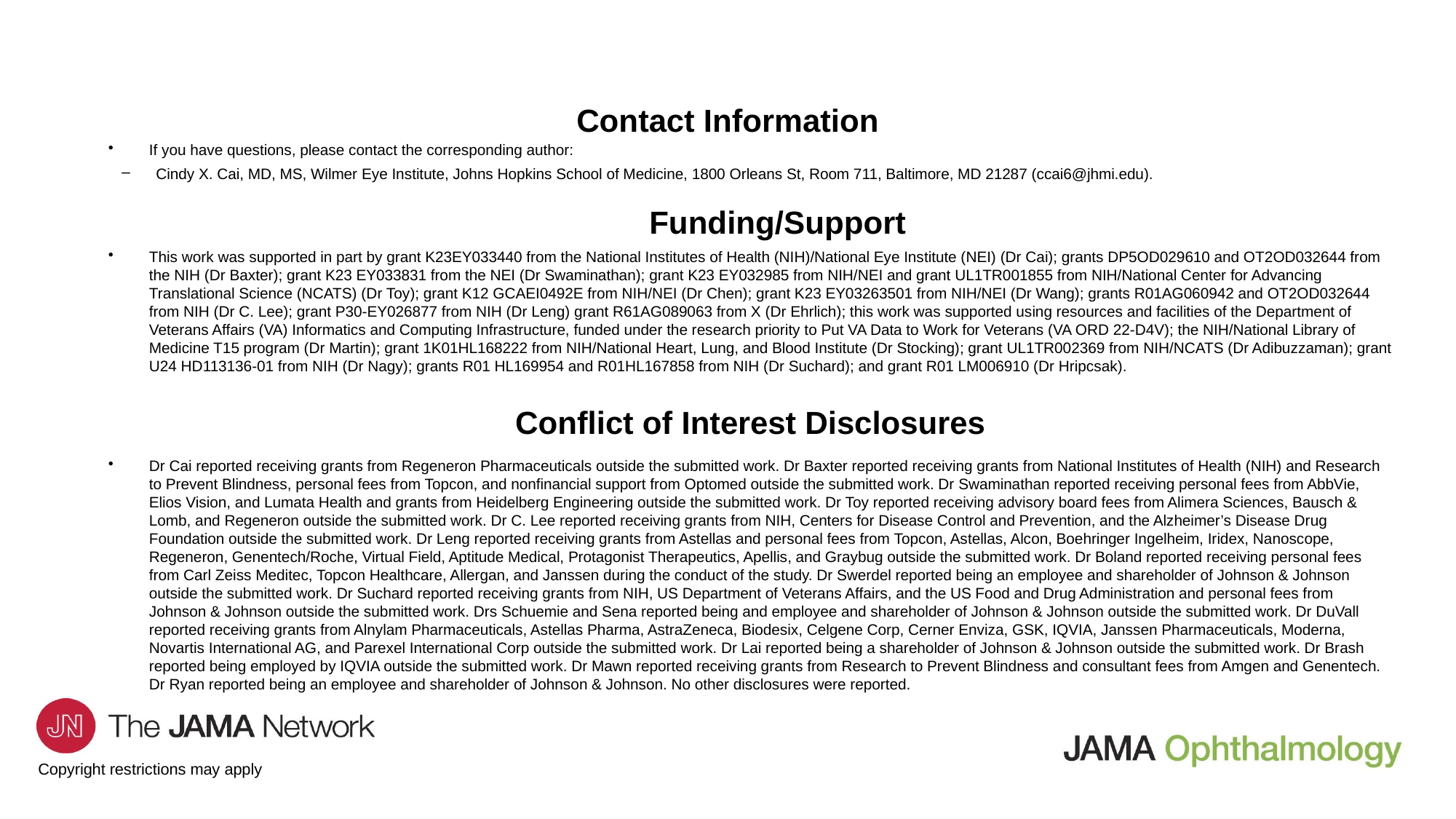

# Contact Information
If you have questions, please contact the corresponding author:
Cindy X. Cai, MD, MS, Wilmer Eye Institute, Johns Hopkins School of Medicine, 1800 Orleans St, Room 711, Baltimore, MD 21287 (ccai6@jhmi.edu).
Funding/Support
This work was supported in part by grant K23EY033440 from the National Institutes of Health (NIH)/National Eye Institute (NEI) (Dr Cai); grants DP5OD029610 and OT2OD032644 from the NIH (Dr Baxter); grant K23 EY033831 from the NEI (Dr Swaminathan); grant K23 EY032985 from NIH/NEI and grant UL1TR001855 from NIH/National Center for Advancing Translational Science (NCATS) (Dr Toy); grant K12 GCAEI0492E from NIH/NEI (Dr Chen); grant K23 EY03263501 from NIH/NEI (Dr Wang); grants R01AG060942 and OT2OD032644 from NIH (Dr C. Lee); grant P30-EY026877 from NIH (Dr Leng) grant R61AG089063 from X (Dr Ehrlich); this work was supported using resources and facilities of the Department of Veterans Affairs (VA) Informatics and Computing Infrastructure, funded under the research priority to Put VA Data to Work for Veterans (VA ORD 22-D4V); the NIH/National Library of Medicine T15 program (Dr Martin); grant 1K01HL168222 from NIH/National Heart, Lung, and Blood Institute (Dr Stocking); grant UL1TR002369 from NIH/NCATS (Dr Adibuzzaman); grant U24 HD113136-01 from NIH (Dr Nagy); grants R01 HL169954 and R01HL167858 from NIH (Dr Suchard); and grant R01 LM006910 (Dr Hripcsak).
Conflict of Interest Disclosures
Dr Cai reported receiving grants from Regeneron Pharmaceuticals outside the submitted work. Dr Baxter reported receiving grants from National Institutes of Health (NIH) and Research to Prevent Blindness, personal fees from Topcon, and nonfinancial support from Optomed outside the submitted work. Dr Swaminathan reported receiving personal fees from AbbVie, Elios Vision, and Lumata Health and grants from Heidelberg Engineering outside the submitted work. Dr Toy reported receiving advisory board fees from Alimera Sciences, Bausch & Lomb, and Regeneron outside the submitted work. Dr C. Lee reported receiving grants from NIH, Centers for Disease Control and Prevention, and the Alzheimer’s Disease Drug Foundation outside the submitted work. Dr Leng reported receiving grants from Astellas and personal fees from Topcon, Astellas, Alcon, Boehringer Ingelheim, Iridex, Nanoscope, Regeneron, Genentech/Roche, Virtual Field, Aptitude Medical, Protagonist Therapeutics, Apellis, and Graybug outside the submitted work. Dr Boland reported receiving personal fees from Carl Zeiss Meditec, Topcon Healthcare, Allergan, and Janssen during the conduct of the study. Dr Swerdel reported being an employee and shareholder of Johnson & Johnson outside the submitted work. Dr Suchard reported receiving grants from NIH, US Department of Veterans Affairs, and the US Food and Drug Administration and personal fees from Johnson & Johnson outside the submitted work. Drs Schuemie and Sena reported being and employee and shareholder of Johnson & Johnson outside the submitted work. Dr DuVall reported receiving grants from Alnylam Pharmaceuticals, Astellas Pharma, AstraZeneca, Biodesix, Celgene Corp, Cerner Enviza, GSK, IQVIA, Janssen Pharmaceuticals, Moderna, Novartis International AG, and Parexel International Corp outside the submitted work. Dr Lai reported being a shareholder of Johnson & Johnson outside the submitted work. Dr Brash reported being employed by IQVIA outside the submitted work. Dr Mawn reported receiving grants from Research to Prevent Blindness and consultant fees from Amgen and Genentech. Dr Ryan reported being an employee and shareholder of Johnson & Johnson. No other disclosures were reported.
